# Nucleotide metabolism in cancer cells fuels a UDP-driven macrophage cross-talk, promoting immunosuppression and immunotherapy resistance

**DOI:** 10.1038/s43018-024-00771-8

**Published:** 2024-06-06

**Authors:** Tommaso Scolaro, Marta Manco, Mathieu Pecqueux, Ricardo Amorim, Rosa Trotta, Heleen H. Van Acker, Matthias Van Haele, Niranjan Shirgaonkar, Stefan Naulaerts, Jan Daniluk, Fran Prenen, Chiara Varamo, Donatella Ponti, Ginevra Doglioni, Ana Margarida Ferreira Campos, Juan Fernandez Garcia, Silvia Radenkovic, Pegah Rouhi, Aleksandar Beatovic, Liwei Wang, Yu Wang, Amalia Tzoumpa, Asier Antoranz, Ara Sargsian, Mario Di Matteo, Emanuele Berardi, Jermaine Goveia, Bart Ghesquière, Tania Roskams, Stefaan Soenen, Thomas Voets, Bella Manshian, Sarah-Maria Fendt, Peter Carmeliet, Abhishek D. Garg, Ramanuj DasGupta, Baki Topal, Massimiliano Mazzone

**Affiliations:** 1 Laboratory of Tumor Inflammation and Angiogenesis, Center for Cancer Biology, VIB, Leuven, Belgium; 2 Laboratory of Tumor Inflammation and Angiogenesis, Center for Cancer Biology, Department of Oncology, KU Leuven, Leuven, Belgium; 3 Department of Visceral, Thoracic and Vascular Surgery, University Hospital Carl Gustav Carus, Technische Universität Dresden, Dresden, Germany; 4 Life and Health Sciences Research Institute, School of Medicine, University of Minho, Campus de Gualtar, Braga, Portugal; 5 ICVS/3B’s-PT Government Associate Laboratory, Braga/Guimarães, Portugal; 6 Department of Imaging and Pathology, Translational Cell and Tissue Research, KU Leuven and University Hospitals Leuven, Leuven, Belgium; 7 Laboratory of Precision Oncology and Cancer Evolution, Genome Institute of Singapore, A*STAR, Singapore, Singapore; 8 Laboratory for Cell Stress & Immunity (CSI), Department of Cellular & Molecular Medicine, KU Leuven, Leuven, Belgium; 9 Laboratory of Ion Channel Research (LICR), VIB-KU Leuven Centre for Brain & Disease Research, Leuven, Belgium; 10 Department of Cellular and Molecular Medicine, KU Leuven, Leuven, Belgium; 11 Department of Medical-Surgical Sciences and Biotechnologies, University of Rome Sapienza, Latina, Italy; 12 Laboratory of Cellular Metabolism and Metabolic Regulation, Center for Cancer Biology, VIB, Leuven, Belgium; 13 Laboratory of Cellular Metabolism and Metabolic Regulation, Department of Oncology, KU Leuven and Leuven Cancer Institute (LKI), Leuven, Belgium; 14 Metabolomics Core Facility, Center for Cancer Biology, VIB, Leuven, Belgium; 15 Metabolomics Core Facility, Center for Cancer Biology, Department of Oncology, KU Leuven, Leuven, Belgium; 16 Unicle Biomedical Data Science, Leuven, Belgium; 17 State Key Laboratory of Oncogenes and Related Genes, Shanghai Cancer Institute, Department of Oncology, Renji Hospital, School of Medicine, Shanghai Jiao Tong University, Shanghai, China; 18 Translation Cell and Tissue Research Unit, Department of Imaging and Pathology, KU Leuven, Leuven, Belgium; 19 Laboratory of Angiogenesis and Vascular Metabolism, Center for Cancer Biology, VIB, Leuven, Belgium; 20 Laboratory of Angiogenesis and Vascular Metabolism, Center for Cancer Biology, Department of Oncology, KU Leuven, Leuven, Belgium; 21 NanoHealth and Optical Imaging Group, Department of Imaging and Pathology, KU Leuven, Leuven, Belgium; 22 Department of Visceral Surgery, KU Leuven and University Hospitals Leuven, Leuven, Belgium

**Keywords:** Cancer, Immunotherapy, Cancer metabolism

## Abstract

Many individuals with cancer are resistant to immunotherapies. Here, we identify the gene encoding the pyrimidine salvage pathway enzyme cytidine deaminase (CDA) among the top upregulated metabolic genes in several immunotherapy-resistant tumors. We show that CDA in cancer cells contributes to the uridine diphosphate (UDP) pool. Extracellular UDP hijacks immunosuppressive tumor-associated macrophages (TAMs) through its receptor P2Y_6_. Pharmacologic or genetic inhibition of CDA in cancer cells (or P2Y_6_ in TAMs) disrupts TAM-mediated immunosuppression, promoting cytotoxic T cell entry and susceptibility to anti-programmed cell death protein 1 (anti-PD-1) treatment in resistant pancreatic ductal adenocarcinoma (PDAC) and melanoma models. Conversely, CDA overexpression in CDA-depleted PDACs or anti-PD-1-responsive colorectal tumors or systemic UDP administration (re)establishes resistance. In individuals with PDAC, high CDA levels in cancer cells correlate with increased TAMs, lower cytotoxic T cells and possibly anti-PD-1 resistance. In a pan-cancer single-cell atlas, *CDA*^high^ cancer cells match with T cell cytotoxicity dysfunction and *P2RY6*^high^ TAMs. Overall, we suggest CDA and P2Y_6_ as potential targets for cancer immunotherapy.

## Abstract (web summary)

Scolaro et al. identify the enzyme cytidine deaminase (CDA) as upregulated in immunotherapy-resistant tumors and find it contributes to the UDP pool, which in turn modulates tumor-associated macrophages to instruct an immune-evasive TME.

## Main

Immunotherapy, including adoptive T cell transfer, cancer vaccines and immune checkpoint blockade (ICB), represents a promising treatment option for individuals with cancer^1^. For instance, programmed cell death protein 1 receptor (PD-1) is an immune checkpoint protein expressed on the cell surface of T cells. By binding its cognate ligand (PD-L1), PD-1 turns down an uncontrolled T cell response by modulating T cell antigen receptor (TCR) signaling. In tumors, cancer cells hijack this pathway by overexpressing PD-L1. Hence, the therapeutic potential of antibodies to PD-1 has been intensely investigated^2^.

Despite the high response rates with prolonged survival in subsets of individuals with melanoma^3^, lung^4^ and renal cancer^5^, ICB failed to show clinical benefit in several other tumors, such as the majority of mismatch repair-proficient colorectal cancers^6^ and pancreatic ductal adenocarcinoma (PDAC)^7^.

PDAC is one of the most aggressive and lethal cancers, with an overall 5-year survival rate of 9%. The projected doubling of PDAC incidence by 2030 would make it the second leading cause of cancer-related death after lung cancer. The majority of individuals present at advanced stages with distant organ metastases and/or locoregional extension, resulting in less than 20% of individuals being eligible for resection at the time of diagnosis^8^. Most therapies, including ICB, are not effective^7^, and the majority of individuals who undergo surgery ultimately relapse^9^. Thus, there is urgent need for treatments that are applicable to most individuals with unresectable tumors or that prevent relapse after surgery. Several approaches to synergize ICB with pharmacological strategies targeting immunosuppressive fibroblasts, myeloid cells or regulatory T cells or cancer vaccines (for example, GVAX) genetically engineered to release immunostimulatory cytokines have been proposed in mouse models or have been tested in the clinic^10–14^. In this context, tumor metabolism can compromise the function and fate of tumor-infiltrating immune cells and favor immunological tolerance^15^.

Cytidine deaminase (CDA) is an evolutionarily conserved enzyme of the pyrimidine salvage pathway responsible for the hydrolytic deamination of free cytidine and deoxycytidine to uridine and deoxyuridine, respectively. In some cancer cell lines, CDA protects newly synthesized DNA from incorporating epigenetically modified forms of cytidine^16^. Although CDA deaminates and inactivates cytidine analogs used as chemotherapeutic agents in cancer treatment (that is, gemcitabine, cytosine arabinoside and 5-azacytidine), thus playing a role in chemoresistance^17–19^, the possible contribution of CDA to the extracellular nucleotide pool and immunotherapy resistance has never been studied.

## Results

### CDA in cancer cells is associated with ICB resistance

**Fig. 1:**
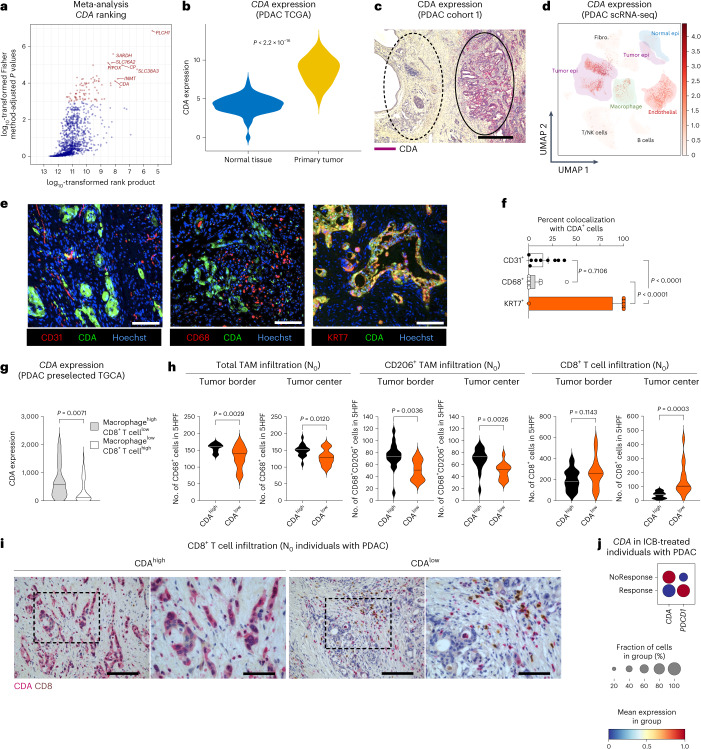
CDA is associated with resistance to immunotherapy. **a**, Meta-analysis on four datasets. Red indicates genes with adjusted *P* values of <1 × 10^–3^; CDA: 9 of 1,321; adjusted *P* value of <1 × 10^–4^. **b**, *CDA* expression in pancreatic cancer (*n* = 183) and normal pancreatic tissues (*n* = 167). **c**, Representative image of CDA staining (purple) in tumor (right ellipse) and adjacent tissue (left ellipse) of an individual with PDAC (cohort 1, *n* = 63); scale bar, 100 µm. **d**, UMAP representing *CDA* expression in different cell populations of pancreatic tissue from treatment-naive individuals with PDAC (*n* = 24). The number of cells analyzed is 83,960; epi, epithelial cells; Fibro., fibroblasts. **e**,**f**, Representative micrographs (**e**) and related quantification (percentage of colocalization; **f**) of CDA (green) with cancer cells; *n* = 9 out of cohort 1; scale bar, 50 µm. **g**, Violin plot of *CDA* expression in macrophage^high^CD8^+^ T cell^low^ (*n* = 64) versus macrophage^low^CD8^+^ T cell^high^ (*n* = 31) individuals with PDAC in TCGA. **h**, Histological analysis of treatment-naive, resectable PDAC tumors (out of cohort 1; stage I–IIa; N_0_). Violin plots showing quantification of total CD68^+^ (left), immunosuppressive CD206^+^ TAMs (middle) and cytotoxic T cells (CD8^+^; right) at the tumor border or center (CDA^high^ *n* = 18 individuals with PDAC; CDA^low^ *n* = 14 individuals with PDAC); 5HPF, five high-power fields. **i**, Representative images of cytotoxic T cell (CD8; brown) infiltration in the tumor core (low magnification on the left and a zoom on the right) of an individual with PDAC (cohort 1, *n* = 63); scale bars, 10 µm (left) and 5 µm (right). **j**, Dot plot of *CDA* and *PDCD1* expression in PDAC tumors from individuals before three cycles of ICB (combined with radiation on the second cycle). Mean expression is shown as color and is standard scaled (binarized), whereas dot size represents the fraction of samples with expression (Response *n* = 2; NoResponse *n* = 5). In **b**–**d** and **f**–**j**, *n* represents the number of individuals. Data were analyzed by unpaired, two-tailed Student’s *t*-tests (**b**, **g** and **h**), one-way analysis of variance (ANOVA) with Tukey’s multiple comparison test (**f**) and Fisher’s combined probability test (one sided; **a**). Data are shown as mean ± s.e.m. Source data

**Extended Data Fig. 1:**
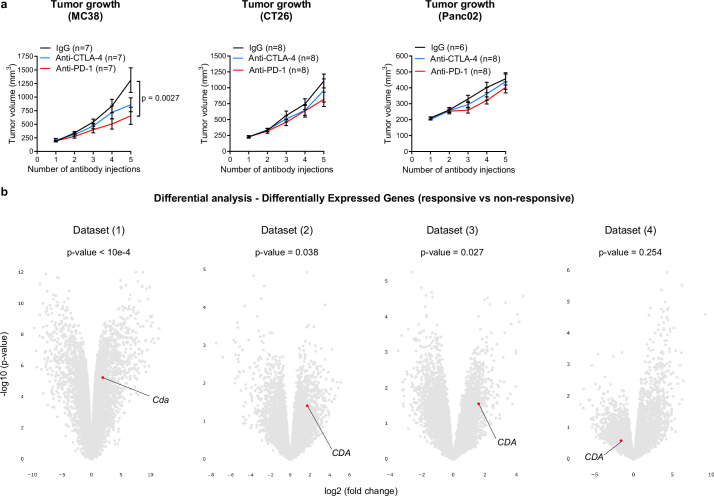
Identification of CDA in cancer cells as metabolic player in immunotherapy resistance. (**a**) Volume of MC38, CT26 and Panc02 tumors, treated with anti-CTLA-4 (blue), anti-PD-1 (red) or control (CTRL) IgG (black line). n=6-8. n represents biological replicates. (**b**) Volcano plots depicting log2 fold change and log10 (p-value) of differentially expressed genes (*Cda* / *CDA* indicated in red) between responsive and non-responsive tumors, for each of the datasets. From left to right, (1; GSE196790) MC38 vs Panc02; murine; anti-CTLA-4 and anti-PD-1 treatment; log2 fold change = 1.93, p-value < 1x10^-4^; (2; GSE78220) metastatic melanoma; human; anti-PD-1 treatment; log2 fold change = 1.73, p-value = 0.038; (3; GSE67501) renal cancer; human; anti-PD-1 treatment; log2 fold change = 1.60, p-value = 0.027; (4; phs000452.v2.p) metastatic melanoma; human; anti-CTLA-4 treatment; log2 fold change = -1.59, p-value = 0.254. Statistical analysis: p value was assessed by two-way RM ANOVA (a), two-sided limma package (b). Graphs show mean ± SEM.

To identify metabolic genes involved in immunotherapy resistance, we performed a meta-analysis on in-house-generated mouse bulk RNA-sequencing (RNA-seq) datasets and three publicly available pretreatment transcriptomic datasets of tumors responsive and resistant to ICB, such as anti-CTLA-4 and anti-PD-1 (refs. ^3,5,20^; Extended Data Fig. 1a). Specifically, we first performed differential analysis between responsive and nonresponsive tumors on each dataset separately. We then ranked the genes by fold change and combined the rank numbers by calculating their rank product (Extended Data Fig. 1b). Finally, we filtered the ranked list for metabolic genes and calculated statistics by combining one-tailed *P* values across studies using Fisher’s method^20^. From the top ten ranked candidates, we focused on *CDA* because nothing is known on pyrimidine metabolism in cancer immunotherapy (Fig. 1a and Extended Data Fig. 1b).

**Extended Data Fig. 2:**
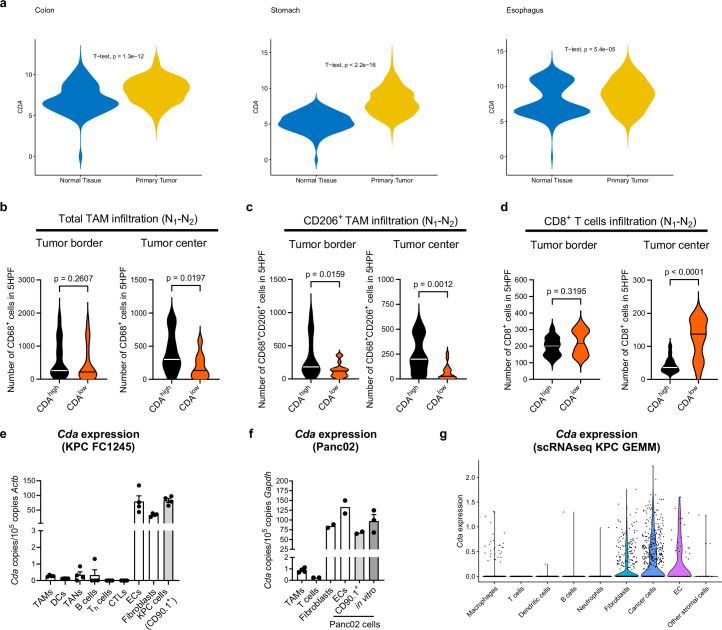
CDA in cancer cells correlates with immunosuppression. (**a**) Violin plot from Xena PanCAN-GTex platform (and selecting for TCGA bulk RNA sequencing datasets only) representing *CDA* expression in colon (Normal Tissue n=308, Primary Tumor n=288), stomach (Normal Tissue n=210, Primary Tumor n=414) and esophagus (Normal Tissue n=653, Primary Tumor n=195) cancer. (**b–d**) Histological analysis of treatment-naïve, resectable PDAC tumors (out of cohort #1; stage IIb-III; N1-N2). (b) Violin plots of histological quantification of total CD68^+^ at tumor border or center. (c) Violin plots of histological quantification of immunosuppressive CD206^+^ tumor-associated macrophages (TAMs) at tumor border or center. (d) Violin plots of histological quantification of cytotoxic T cells (CD8^+^) at tumor border or center. CDA^high^ n=14 and CDA^low^ n=17 PDAC patients. (**e**) *Cda* expression in different cell populations sorted from murine orthotopic KPC FC1245 tumors. n=4. n represents biological replicates. (**f**) *Cda* expression in different cell populations sorted from murine s.c. Panc02 tumors (sorted Panc02 CD90.1+ cells and in vitro Panc02 cells in grey as reference). TAMs n=4; in vitro Panc02 n=3; all the others n=2. n represents biological replicates. (**g**) *Cda* expression in different cell populations in mouse PDAC (KPC GEMM) from a publicly available single-cell RNA-sequencing (scRNAseq) dataset. n=4. n represents biological replicates. Statistical analysis: p value was assessed by unpaired, two-tailed Student’s t-test (a, b-d). Graphs show mean ± SEM.

Of all the tumor types included in the meta-analysis, we focused on PDAC for the medical needs related to this aggressive and refractory tumor. Using the Xena PanCAN-GTex platform (and selecting for The Cancer Genome Atlas (TCGA) bulk RNA-seq datasets only), *CDA* expression was strongly upregulated in pancreatic tumor versus normal tissue (Fig. 1b). This finding was corroborated by immunohistochemistry for CDA in an independent cohort of 63 human treatment-naive PDAC samples (referred to as cohort 1, stage I–III; N_0_–N_2_; Supplementary Table 1), revealing variable but selective expression of CDA in cancer cells but not in adjacent nontumor tissue (Fig. 1c). Furthermore, *CDA* expression was also significantly upregulated in colon, gastric and esophageal cancers compared to in their normal counterparts (Extended Data Fig. 2a).

Closer examination of the tumor microenvironment (TME) using a human PDAC publicly available single-cell RNA-seq (scRNA-seq) dataset^21^ revealed *CDA* expression mainly in cancer cells and endothelial cells (ECs) with low expression in macrophages. The normal pancreatic epithelium (both acinar and ductal cells), fibroblasts and other immune cells, such as T, B and natural killer (NK) cells, showed no detectable *CDA* expression (Fig. 1d). However, data validation by coimmunostainings of nine PDAC sections randomly selected from the previous cohort 1 confirmed the histopathological observation that, at the protein level, CDA was expressed in neoplastic ducts, but not in macrophages or ECs (Fig. 1e,f).

Together, CDA induction in cancer cells and its correlation with ICB resistance suggest its possible role in hampering antitumor responses and immunotherapy efficacy.

### CDA in individuals with PDAC correlates with immunosuppression

By preselecting PDAC tumors from the TCGA PDAC cohort according to their highly immunosuppressive/low immunogenic landscape profile (that is, PDAC tumors showing high enrichment of a macrophage signature but decreased expression of a CD8^+^ T cell signature), we observed that *CDA* expression was higher in this group than in the less immunosuppressive groups (that is, low enrichment of a macrophage signature but higher expression of a CD8^+^ T cell signature; Fig. 1g).

To validate these findings, we selected two homogeneous subsets of individuals with PDAC out of the above-mentioned cohort 1. In the first subset, we included 32 individuals with early-stage PDAC (stage I–IIa; N_0_), whereas in the second subset, we included 31 individuals with late-stage PDAC (stage IIb–III; N_1_–N_2_). To reduce the effect of intratumoral variability, the pathologist used macroblocks with cross-sections of the whole surgical specimen to evaluate areas of interest and identify margin and center areas. Individuals were dichotomized into CDA^low^ and CDA^high^ cancers (Supplementary Table 1). We found a significant correlation between CDA expression (set 1: CDA^low^ *n* = 14, CDA^high^ *n* = 18; set 2: CDA^low^ *n* = 17, CDA^high^ *n* = 14) in malignant ductal cells with intratumoral CD68^+^ tumor-associated macrophage (TAM) infiltration or the CD206^+^ immunosuppressive fraction, both at the tumor border and center (Fig. 1h and Extended Data Fig. 2b,c). Accordingly, CD8^+^ T cell infiltration in the tumor center was significantly lower in CDA^high^ PDAC, but it did not change at the tumor rim (Fig. 1h,i and Extended Data Fig. 2d). Vice versa, low CDA expression matched with reduced total and CD206^+^ TAMs but with increased CD8^+^ T cell infiltration (Fig. 1h,i and Extended Data Fig. 2b–d).

Moreover, the analysis of pretreatment bulk RNA-seq data from an independent set of seven PDAC tumors^22^ revealed an association between high *CDA* expression and resistance to ICB (in combination with radiotherapy). Conversely, low *CDA* expression was associated with (partial) response (Fig. 1j).

Together, these data reinforce the idea that CDA upregulation may play an important role in shaping the immunosuppressive landscape of human PDAC and other tumors, possibly mounting immunotherapy resistance.

### Targeting CDA in PDAC cancer cells promotes anti-PD-1 efficacy

To assess the possible link between CDA expression in cancer cells and ICB resistance in PDAC, we used two mouse PDAC tumor engraftment models: orthotopic KPC tumors and subcutaneous (s.c.) Panc02 tumors. The Panc02 model is not reflective of human disease because it does not carry KRAS activation (occurring in 90% of human PDAC) and it presents more mutations and antigens than in human PDAC, but it is still resistant to ICB^23^. Instead, KPC cells, isolated from *LSL*-*Kras*^G12D/+^; *LSL-Trp53*^R172H/+^; *Pdx1:cre*^Tg/+^ mice, carry the most frequent oncogenic features of human PDAC, namely KRAS activation and mutant p53 with loss of the wild-type function^24^.

First, we examined *Cda* expression in sorted cells from these two tumor models. In-house quantitative PCR with reverse transcription (RT–qPCR) data showed *Cda* expression in cancer cells and ECs, but not in macrophages or T cells (Extended Data Fig. 2e,f). In contrast to humans, some *Cda* expression was found in cancer-associated fibroblasts (Extended Data Fig. 2e,f). Publicly available scRNA-seq data from autochthonous tumors in genetically engineered KPC mice^11^ showed the same pattern as observed in KPC tumor allografts (Extended Data Fig. 2g). Taken together, CDA is enriched in the cancer epithelial compartment in both humans and mice.

**Extended Data Fig. 3:**
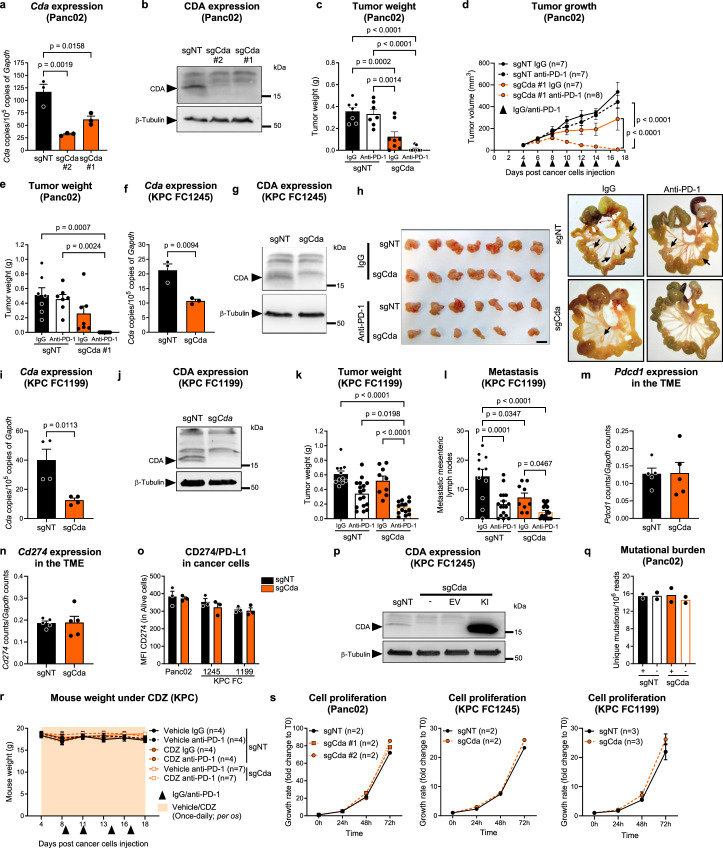
Tools’ validation for the study of CDA’s role in immunotherapy resistance. (**a-b**) (a) *Cda* expression in control (sgNT) and sgCda #1 or sgCda #2 (otherwise called sgCda) ^Panc02^ cells. n=3. n represents independently collected cell seedings. (b) CDA expression in sgNT and sgCda #1 or sgCda #2 Panc02 cells. Representative out of 3 independent experiments. Arrowhead shows CDA specific band. (**c-e**) (c) Weight of sgNT and sgCda s.c. Panc02 tumors, treated with anti-PD-1 or control IgG. sgNT (IgG) n=7, sgNT (anti-PD-1) n=7, sgCda (IgG) n=8, sgCda (anti-PD-1) n=9. (d-e) Growth and weight of sgNT and sgCda #1 s.c. Panc02 tumors, treated with anti-PD-1 or IgG. Treatment regimen is indicated in (d) by the black arrowheads. sgNT (IgG) n=7, sgNT (anti-PD-1) n=7, sgCda #1 (IgG) n=7, sgCda #1 (anti-PD-1) n=8. n represents biological replicates. (**f-g**) (f) *Cda* expression in sgNT and sgCda KPC FC1245 cells. n=3. n represents independently collected cell seedings. (g) CDA expression in sgNT and sgCda KPC FC1245 cells. Representative out of 3 independent experiments. Arrowhead shows CDA specific band. (**h**) Representative images of tumors (left) and metastatic mesenteric lymph nodes (right; arrow) of sgNT and sgCda orthotopic KPC FC1245 tumor-bearing mice, treated with anti-PD-1 or IgG. Scale bar, 1 cm. (**i-j**) (i) *Cda* expression in sgNT and sgCda KPC FC1199 cells. n=4. n represents independently collected cell seedings. (j) CDA expression in sgNT and sgCda KPC FC1199 cells. Representative out of 3 independent experiments. Arrowhead shows CDA specific band. (**k-l**) (k) Weight of sgNT and sgCda orthotopic KPC FC1199 tumors, treated with anti-PD-1 or IgG. (l) Quantification of metastatic mesenteric lymph nodes in sgNT and sgCda KPC FC1199 tumor-bearing mice, treated with anti-PD-1 or IgG. (k, l) sgNT (IgG) n=11, sgNT (anti-PD-1) n=17, sgCda (IgG) n=9, sgCda (anti-PD-1) n=14. n represents biological replicates. Data are representative of a pool of two independent experiments. (**m-n**) *Pdcd1* and *Cd274* expression in bulk RNAseq from Panc02 sgNT and sgCda tumors. n=5. n represents biological replicates. (**o**) CD274 expression (MFI in alive cells) in sgNT or sgCda Panc02, KPC FC1245 and KPC FC1199 cells. n=3. n represents independently collected cell seedings. (**p**) CDA expression in sgNT KPC FC1245, non-transduced (referred to as “-“ in the panel), as well as sgCda KPC FC1245 cells transduced with an empty vector (EV) or with a vector overexpressing *Cda* (CDA KI) by Western Blot. Representative out of 3 independent experiments. Arrowhead shows CDA specific band. (**q**) Mutational burden of Panc02 sgNT and sgCda cells. Cas9 transcription was induced (+) or not (-) with doxycycline (GSE196790). n=2. n represents independently collected cell seedings. (**r**) Body weight of sgNT or sgCda KPC FC1245 tumor bearing-mice, treated with CDZ/control vehicle, in combination with α-PD-1 or IgG. Vehicle-sgNT (IgG) n=4, vehicle-sgNT (anti-PD-1) n=4, CDZ-sgNT (IgG) n=4, CDZ-sgNT (anti-PD-1) n=4, vehicle-sgCda (anti-PD-1) n=7 and CDZ-sgCda (anti-PD-1) n=7. Treatment regimen is indicated by the black arrowheads. n represents independently collected cell seedings. Data are representative of a pool of two independent experiments. (**s**) Cell proliferation of control and *Cda*-depleted Panc02, KPC FC1245 and KPC FC1199 cells. n=2-3. n represents independently collected cell seedings. Statistical analysis: p value was assessed by one-way ANOVA with Tukey’s multiple comparison test (a), two-way ANOVA with Tukey’s multiple comparison test (c, e, k, l,), two-way RM ANOVA (d and s), unpaired, two-tailed Student’s t-test (f, i, m, n), multiple unpaired, two-tailed Student’s t-test (o). Graphs show mean ± SEM.

At this point, we genetically targeted *Cda* in mouse pancreatic cancer cell lines via CRISPR–Cas9. *Cda* targeting in Panc02 cells was achieved by testing two different single guide RNAs (sgCda 1 or sgCda 2, the latter referred to as sgCda) and a nontargeting sgRNA (sgNT) as a control (Extended Data Fig. 3a,b). KPC cells (that is, FC1245 and FC1199) were engineered with one of the two guide RNAs only (namely, sgCda; Extended Data Fig. 3f,g,i,j). CDA targeting did not alter in vitro proliferation (Extended Data Fig. 3s).

**Fig. 2:**
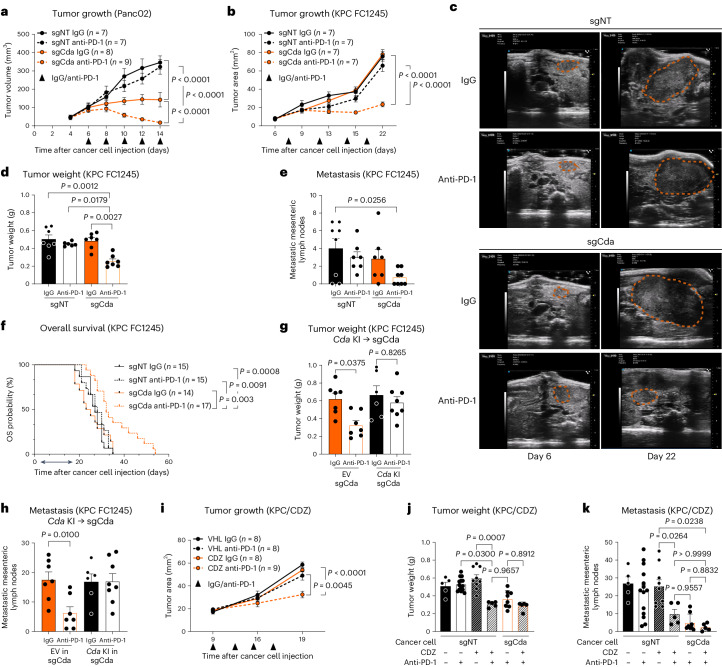
Targeting CDA improves anti-PD-1 therapy efficacy. **a**, Growth of sgNT (control) and sgCda s.c. Panc02 tumors treated with anti-PD-1 or control IgG (sgNT (IgG) *n* = 7, sgNT (anti-PD-1) *n* = 7, sgCda (IgG) *n* = 8, sgCda (anti-PD-1) *n* = 9). The treatment regimen is indicated by the black arrowheads. **b**, Ultrasound-guided longitudinal measurements of sgNT and sgCda orthotopic KPC FC1245 tumors treated with anti-PD-1 or control IgG (sgNT (IgG) *n* = 7, sgNT (anti-PD-1) *n* = 7, sgCda (IgG) *n* = 7, sgCda (anti-PD-1) *n* = 7). **c**, Representative images (ultrasound) at day 6 and day 22 after cancer cell injection of sgNT and sgCda KPC FC1245 tumors. **d**, Weight of KPC FC1245 sgNT and sgCda tumors at end stage treated with anti-PD-1 or control IgG (sgNT (IgG) *n* = 7, sgNT (anti-PD-1) *n* = 6, sgCda (IgG) *n* = 7, sgCda (anti-PD-1) *n* = 7). **e**, Quantification of metastatic mesenteric lymph nodes in sgNT and sgCda KPC FC1245 tumor-bearing mice treated with anti-PD-1 or control IgG (sgNT (IgG) *n* = 8, sgNT (anti-PD-1) *n* = 7, sgCda (IgG) *n* = 7, sgCda (anti-PD-1) *n* = 8). Treatment regimen is indicated in **b** by the black arrowheads. **f**, Kaplan–Meier curves of mice bearing tumors derived from sgNT and sgCda KPC FC1245 clones treated with anti-PD-1 or control IgG (sgNT (IgG) *n* = 15, sgNT (anti-PD-1) *n* = 15, sgCda clones (IgG) *n* = 14, sgCda clones (anti-PD-1) *n* = 17). Data are representative of a pool of three independent experiments; OS, overall survival. **g**, Weight of control (EV in sgCda) and *Cda* knock-in (*Cda* KI in sgCda) tumors treated with anti-PD-1 or control IgG. **h**, Quantification of metastatic mesenteric lymph nodes in control and *Cda* knock-in tumor-bearing mice treated with anti-PD-1 or control IgG (in **g**–**h**, EV (IgG) *n* = 7, EV (anti-PD-1) *n* = 7, *Cda* KI (IgG) *n* = 6, *Cda* KI (anti-PD-1) *n* = 8). Data are representative of a pool of two independent experiments. **i**, Ultrasound-guided longitudinal measurements of orthotopic KPC FC1245 tumors treated with CDZ or vehicle (VHL) and anti-PD-1 or control IgG (vehicle (IgG) *n* = 8, VHL (anti-PD-1) *n* = 8, CDZ (IgG) *n* = 8, CDZ (anti-PD-1) *n* = 9). Treatment regimen is indicated by the black arrowheads. **j**, Weight of sgNT and sgCda KPC FC1245 tumors treated with CDZ or vehicle and anti-PD-1 or IgG. **k**, Quantification of metastatic mesenteric lymph nodes in CDZ- or vehicle-treated sgNT and sgCda KPC FC1245 tumor-bearing mice in combination with anti-PD-1 or IgG (in **j**–**k**, vehicle sgNT (IgG) *n* = 5, vehicle sgNT (anti-PD-1) *n* = 14, CDZ sgNT (IgG) *n* = 9, CDZ sgNT (anti-PD-1) *n* = 5, vehicle sgCda (IgG) *n* = 8, CDZ sgCda (anti-PD-1) *n* = 5). In **a**, **b** and **d**–**k**, *n* represents biological replicates. Data were analyzed by by two-way repeated measures ANOVA (**a**, **b** and **i**), two-way ANOVA with Tukey’s multiple comparison test (**d**, **e**, **g**, **h**, **j** and **k**) or log-rank hypothesis test (Mantel–Cox test; **f**). Data are shown as mean ± s.e.m. Source data

Subsequently, sgCda or sgNT Panc02 cells were injected s.c., while KPC FC1245 and FC1199 cells were inoculated orthotopically in C57BL/6 mice and treated with IgG or anti-PD-1. CDA targeting in Panc02 cancer cells resulted in decreased tumor growth and weight and complete regression following anti-PD-1 treatment compared to resistant control (sgNT) tumors (Fig. 2a and Extended Data Fig. 3c–e). By contrast, longitudinal kinetics via ultrasound or end-stage analysis of orthotopically engrafted KPC FC1245 tumors showed that sgCda did not achieve any tumor growth inhibition (Fig. 2b–d and Extended Data Fig. 3h). However, although control (sgNT) tumors displayed no to very little response to anti-PD-1 treatment, mice engrafted with sgCda cancer cells displayed a tumor reduction of 40% to 70% compared to IgG-treated sgNT controls (Fig. 2b–d and Extended Data Fig. 3h). Furthermore, mesenteric lymph node metastases (evaluated macroscopically as in Mazzone et al. ^25^ and Casazza et al.^26^) were very few in the sgCda plus anti-PD-1 condition (Fig. 2e and Extended Data Fig. 3h). Consistently, genetic inhibition of *Cda* significantly improved the survival rate of KPC FC1245 tumor-bearing mice after anti-PD-1 treatment, whereas the three other conditions were unchanged (Fig. 2f), supporting the idea that CDA targeting overcomes anti-PD-1 resistance. Similar findings were observed by using the KPC FC1199 clone (Extended Data Fig. 3k,l). Of note, the in vivo levels of *Pdcd1* (encoding PD-1) or *Cd274* (encoding the PD-1 ligand PD-L1), both in vivo and in cultured cancer cells, were not altered by CDA depletion (Extended Data Fig. 3m–o).

We then reintroduced a cDNA encoding CDA in sgCda KPC FC1245 cells and injected them orthotopically (Extended Data Fig. 3p). Re-expression of CDA re-established anti-PD-1 resistance, as suggested by the increase in tumor weight and mesenteric lymph node metastases to the levels observed in IgG-treated groups (Fig. 2g,h). This experiment excludes off-target effects and proves that tumor regression is not linked to neoantigen formation. Mutational burden in sgNT and sgCda cancer cells corroborated this conclusion (Extended Data Fig. 3q).

To determine whether pharmacological blockade of CDA could be exploited therapeutically, we used the well-known CDA inhibitor cedazuridine (CDZ), which is clinically used in combination with decitabine in myeloid malignancies^27,28^. When administered orally to KPC FC1245 tumor-bearing mice in combination with IgG or anti-PD-1, CDZ did not cause overt toxicity (Extended Data Fig. 3r). Combined CDZ and anti-PD-1 therapy decreased tumor growth (as assessed longitudinally by ultrasound), end-stage tumor weight and mesenteric lymph node metastases (Fig. 2i–k). We did not observe any additional effect of CDZ when treating sgCda tumors, suggesting that the phenotype is due to CDA inhibition in cancer cells only (Fig. 2j,k).

Thus, both genetic and pharmacological CDA inhibition in PDAC models overcome immunotherapy resistance.

### Targeting CDA in PDAC cancer cells alters the TME

**Fig. 3:**
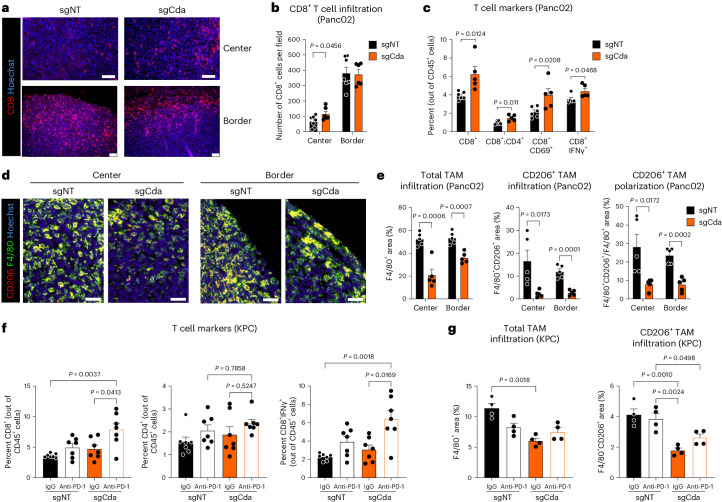
Targeting CDA skews TAMs toward a T cell stimulatory phenotype. **a**–**c**, T cell immune cell landscape in sgNT and sgCda (IgG-treated) s.c. Panc02 tumors. Representative micrographs of cytotoxic CD8^+^ T cells of sgNT and sgCda Panc02 tumors (center, top; border, bottom; **a**) and related histological quantification are shown (**b**; sgNT (center) *n* = 7, sgNT (border) *n* = 7, sgCda (center) *n* = 6, sgCda (border) *n* = 6). **c**, Flow cytometric quantification of cytotoxic T cells (CD8^+^), CD8^+^:CD4^+^ T cell ratio and activated cytotoxic T cells (CD8^+^CD69^+^ and CD8^+^IFNγ^+^) in sgNT and sgCda (IgG-treated) Panc02 tumors (CD8^+^IFNγ^+^, sgNT *n* = 5 and sgCda *n* = 5; all others, sgNT *n* = 6 and sgCda *n* = 5); scale bars, 50 µm (top) and 10 µm (bottom). **d**,**e**, Macrophage immune landscape in sgNT and sgCda (IgG-treated) s.c. Panc02 tumors. Representative micrographs (**d**) and related histological quantification (**e**) of total TAM infiltration (left; percentage of F4/80^+^ cells out of total area), CD206^+^ TAM infiltration (middle; percentage of F4/80^+^CD206^+^ cells out of total area) and CD206^+^ TAM polarization (right; F4/80^+^CD206^+^ cells out of F4/80^+^ area) in sgNT and sgCda (IgG-treated) Panc02 tumors (center and border) are shown (sgNT (center) *n* = 5, sgNT (border) *n* = 5, sgCda (center) *n* = 5, sgCda (border) *n* = 5); scale bar, 50 µm. **f**, T cell immune cell landscape in sgNT and sgCda orthotopic KPC FC1245 tumors. Flow cytometric quantification of cytotoxic T cells (left; CD8^+^), helper T cells (middle; CD4^+^) and activated T cells (right; CD8^+^IFNγ^+^) in sgNT and sgCda orthotopic KPC tumors treated with anti-PD-1 or control IgG (sgNT (IgG) *n* = 7, sgNT (anti-PD-1) *n* = 7, sgCda (IgG) *n* = 7, sgCda (anti-PD-1) *n* = 7). **g**, Macrophage immune landscape in sgNT and sgCda orthotopic KPC FC1245 tumors. Histological quantification of total TAM infiltration (left; percentage of F4/80^+^ cells out of total area) and CD206^+^ TAM infiltration (right; percentage of F4/80^+^CD206^+^ cells out of total area) in sgNT and sgCda orthotopic KPC tumors treated with anti-PD-1 or control IgG (sgNT (IgG) *n* = 4, sgNT (anti-PD-1) *n* = 4, sgCda (IgG) *n* = 4, sgCda (anti-PD-1) *n* = 4). In **b** and **c** and **e**–**g**, *n* represents biological replicates. Data were analyzed by multiple unpaired, two-tailed Student’s *t*-tests (**b**, **c** and **e**), two-way ANOVA with Tukey’s multiple comparison test (**f** and **g**) or two-way repeated measures ANOVA (**h**). Data are shown as mean ± s.e.m. Source data

**Extended Data Fig. 4:**
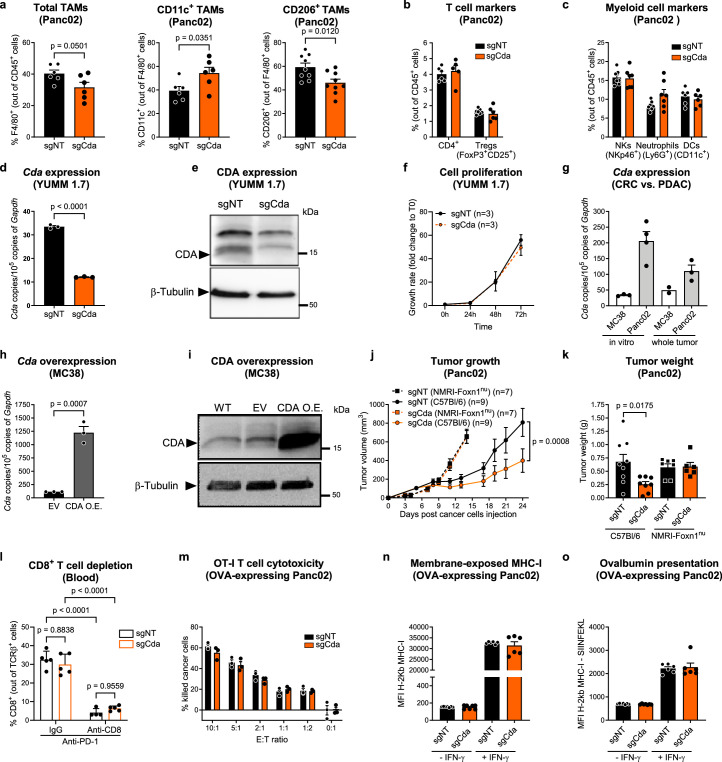
CDA inhibition re-shapes the tumor immune microenvironment. (**a**) Flow cytometric quantification of (left) total (F4/80^+^), (middle) immunostimulatory (CD11c^+^F4/80^+^), and (right) immunosuppressive (CD206^+^F4/80^+^) tumor-associated macrophages (TAMs) in IgG-treated sgNT and sgCda Panc02 tumors. (right) sgNT (n=9) and sgCda (n=9); (left and middle) sgNT (n=6) and sgCda (n=6). n represents biological replicates. (**b-c**) (b) Flow cytometric quantification of intratumoral helper (CD4^+^) and regulatory (CD4^+^Foxp3^+^CD25^+^) T cells or (c) total natural killer cells (NKp46^+^), neutrophils (Ly6G^+^), dendritic cells (CD11c^+^) in IgG-treated sgNT and sgCda Panc02 tumors. (b) CD4^+^, sgNT (n=7) and sgCda (n=6); Tregs, sgNT (n=6) and sgCda (n=5); (c) DCs, sgNT (n=6) and sgCda (n=6); all the others, sgNT (n=7) and sgCda (n=7). n represents biological replicates. (**d-e**) (d) *Cda* expression in sgNT and sgCda YUMM 1.7 cells. n=3. n represents independently collected cell seedings. (**e**) CDA expression in sgNT and sgCda YUMM 1.7 cells. n represents independently collected cell seedings. Arrowhead shows CDA specific band. (**f**) Cell proliferation of sgNT and sgCda YUMM 1.7 cells. n=3. n represents independently collected cell seedings. (g) *Cda* expression in (left) MC38 and Panc02 cells and (right) whole tumors. MC38 cells n=3; MC38 tumor n=2; Panc02 cells n=4; Panc02 tumor n=3. n represents (in vitro) independently collected cell seedings or (tumor) biological replicates. (**h-i**) (h) *Cda* expression in MC38 cells transduced with empty vector (EV) or a vector overexpressing *Cda* (CDA O.E). n=3. n represents independently collected cell seedings. (i) CDA expression in non-transduced (referred to as WT), as wells as EV or CDA O.E MC38 cells. n represents independently collected cell seedings. Arrow shows CDA specific band. (**j-k**) (j) Growth and (k) weight of sgNT and sgCda s.c. Panc02 tumors in nude (NMRI-Fox1nu) or immunocompetent (C57BL/6) mice. (j) sgNT-C57BL/6 n=9, sgCda-C57BL/6 n=9, sgNT-NMRI-Fox1nu n=7, sgCda-NMRI-Fox1nu n=7; (k) sgNT-C57BL/6 n=9, sgCda-C57BL/6 n=8, sgNT-NMRI-Fox1nu n=7, sgCda-NMRI-Fox1nu n=6. n represents biological replicates. Treatment regimen is indicated in (j) by the black arrowheads. (**l**) Flow cytometric quantification (at end-stage) of cytotoxic T cells (% of CD8^+^ out of TCRβ^+^ cells) in blood of KPC FC1245 tumor-bearing mice, treated with anti-PD-1 and IgG or anti-CD8 depleting antibody (anti-CD8). sgNT (IgG) n=5, sgCda (IgG) n=5, sgNT (anti-CD8) n=4, sgCda (anti-CD8) n=5. n represents biological replicates. (**m**) OT-I CD8^+^ T-cell killing capacity of sgNT and sgCda OVA-expressing Panc02 cells. n=3. n represents independently collected cell seedings. (**n-o**) Flow cytometric quantification of (n) total H-2Kb MHC class I and (o) H-2Kb MHC class I bound to the OVA-derived peptide SIINFEKL in OVA-expressing sgNT and sgCda Panc02 cells, at baseline or after IFN-y stimulation. sgNT (- IFN-y) n=7, sgCda (- IFN-y) n=7, sgNT (+ IFN-y) n=6, sgCda (+ IFN-y) n=6. n represents independently collected cell seedings. Statistical analysis: p value was assessed by multiple unpaired, two-tailed Student’s t-test (a, b, n-o), unpaired, two-tailed Student’s t-test (c, d, h), two-way RM ANOVA (f and j), two-way ANOVA with Tukey’s multiple comparison test (k), two-way ANOVA with Sidak’s multiple comparison test (l, m). Graphs show mean ± SEM.

Histological analysis revealed that in control (sgNT) s.c. Panc02 tumors, cytotoxic CD8^+^ T cells grouped at the tumor border, whereas CD8^+^ T cells were missing in the tumor center, disclosing the inability of CD8^+^ T cells to invade the tumor. However, intratumoral CD8^+^ T cell infiltration was significantly increased in CDA-targeted tumors (Fig. 3a,b). Flow cytometry analysis confirmed the increased infiltration of total and activated CD8^+^ T cells in IgG-treated sgCda Panc02 tumors (Fig. 3c). Conversely, both total and immunosuppressive CD206^+^ macrophages were diminished in sgCda Panc02 tumors (Fig. 3d,e and Extended Data Fig. 4a). No changes were observed in CD4^+^ or regulatory T cells, neutrophils, NK cells or dendritic cells (DCs; Extended Data Fig. 4b,c). The effect of combined CDA targeting and anti-PD-1 could not be analyzed due to tumor regression.

In the orthotopic KPC model, CDA targeting alone (IgG group) did not alter the numbers of total or activated CD8^+^ T cells (Fig. 3f), which were, however, higher after anti-PD-1 treatment than in anti-PD-1-treated sgNT tumors. CD4^+^ T cells were, in general, unaffected (Fig. 3f). By contrast, total and CD206^+^ TAM infiltration was reduced by CDA depletion, and anti-PD-1 treatment did not change this effect (Fig. 3g).

Thus, CDA inhibition in cancer cells breaks immunosuppression and enables T cell response to anti-PD-1.

### CDA contribution to anti-PD-1 resistance in other tumor types

**Fig. 4:**
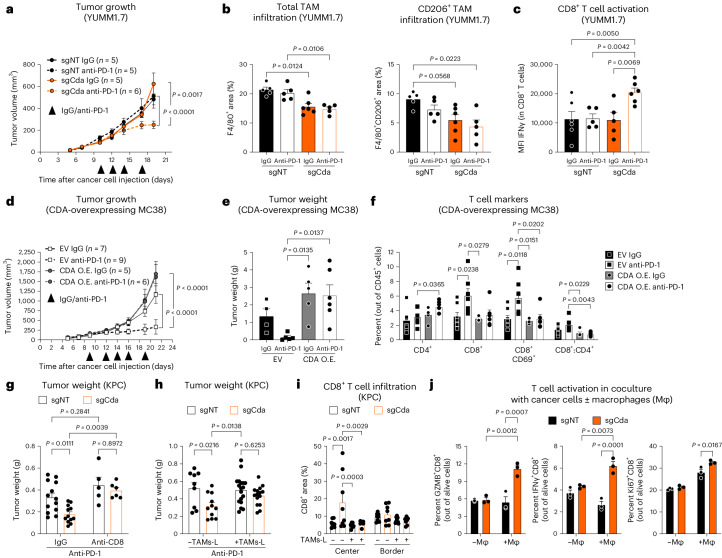
Targeting CDA overcomes anti-PD-1 resistance by shaping the TME. **a**–**c**, Tumor volume (**a**), histological quantification of total TAM infiltration (percentage of F4/80^+^ cells out of total area) and CD206^+^ TAM infiltration (percentage of F4/80^+^CD206^+^ cells out of total area; **b**) and flow cytometric quantification of activated cytotoxic CD8^+^ T cells (mean fluorescence intensity (MFI) IFNγ) in sgNT and sgCda orthotopic YUMM1.7 tumors treated with anti-PD-1 or control IgG (**c**; sgNT (IgG) *n* = 5, sgNT (anti-PD-1) *n* = 5, sgCda (IgG) *n* = 5, sgCda (anti-PD-1) *n* = 6 (**a**); sgNT (IgG) *n* = 5, sgNT (anti-PD-1) *n* = 5, sgCda (IgG) *n* = 6, sgCda (anti-PD-1) *n* = 5 (**b**); sgNT (IgG) *n* = 6, sgNT (anti-PD-1) *n* = 5, sgCda (IgG) *n* = 5, sgCda (anti-PD-1) *n* = 6 (**c**)). Treatment regimen is indicated in **a** by the black arrowheads. **d**,**e**, Volume (**d**) and weight of MC38 tumors overexpressing CDA (CDA O.E.) or their control (EV) treated with anti-PD-1 or control IgG (**e**). **f**, Flow cytometric quantification of intratumoral helper T cells (CD4^+^), cytotoxic T cells (CD8^+^), early activated T cells (CD8^+^CD69^+^) and CD8^+^:CD4^+^ T cell ratio (IgG *n* = 5, CDA O.E. (anti-PD-1) *n* = 6, EV (IgG) *n* = 4–7, EV (anti-PD-1) *n* = 5–9). Treatment regimen is indicated in **d** by the black arrowheads. **g**, Weight of sgNT and sgCda orthotopic KPC FC1245 tumors in mice treated with IgG or CD8-depleting antibody. All mice were treated with anti-PD-1 (sgNT (IgG) *n* = 13, sgNT (anti-CD8) *n* = 5, sgCda (IgG) *n* = 12, sgCda (anti-CD8) *n* = 6). Data are representative of a pool of two independent experiments. **h**, Weight of sgNT and sgCda orthotopic KPC FC1245 tumors resulting from cancer cells implanted alone or with TAMs-L (sgNT (–TAMs-L) *n* = 9, sgNT (+TAMs-L) *n* = 18, sgCda (–TAMs-L) *n* = 11, sgCda (+TAMs-L) *n* = 15). Data are representative of a pool of two independent experiments. **i**, Histological quantification of cytotoxic CD8^+^ T cells (center and border; sgNT (–TAMs-L) *n* = 8, sgNT (+TAMs-L) *n* = 9, sgCda (–TAMs-L) *n* = 8, sgCda (+TAMs-L) *n* = 7). All mice were treated with anti-PD-1. **j**, Flow cytometric quantification of activated (GZMB^+^ and INFγ^+^) and proliferating (Ki67^+^) OT-I CD8^+^ T cells in coculture with OVA-expressing sgNT or sgCda Panc02 cells with or without BMDMs (Mφ; *n* = 3). In **a**–**i**, *n* represents biological replicates, whereas in **j**, *n* represents independently collected cell seedings. Data were analyzed by two-way ANOVA with Tukey’s multiple comparison test (**b**, **c**, **g**, **h** and **j**), two-way repeated measures ANOVA (**a** and **d**), one-way ANOVA with Tukey’s multiple comparison test (**e** and **f**) or two-way ANOVA with a Sidak’s multiple comparison test (**i**). Data are shown as mean ± s.e.m. Source data

We then extended our findings to other tumor types included in the initial meta-analysis. We chose the orthotopic YUMM1.7 melanoma cell line because it is anti-PD-1 resistant and presents genetic alterations seen in a large subset of human melanomas (*Braf*^V600E/+^; *Pten*^−/−^; *Cdkn2*^−/−^)^29^. CDA depletion in combination with anti-PD-1 treatment reduced tumor growth (Fig. 4a and Extended Data Fig. 4d,e). Moreover, CDA targeting was sufficient to reduce both total and CD206^+^ TAM infiltration (Fig. 4b), whereas its combination with anti-PD-1 treatment only enhanced CD8^+^ T cell activation (Fig. 4c). In vitro proliferation of sgNT and sgCda YUMM1.7 cells did not differ (Extended Data Fig. 4f).

We also assessed whether CDA overexpression could establish ICB resistance. We picked the anti-PD-1-responsive colorectal cancer model (MC38) included in our meta-analysis that expresses low levels of *Cda* (Extended Data Fig. 4g). *Cda* overexpression in MC38 cells (Extended Data Fig. 4h,i) rendered the tumor more aggressive and resistant to anti-PD-1 therapy (Fig. 4d,e) as a result of impaired induction of a T cell response following anti-PD-1 administration. Although empty vector (EV) control MC38 tumors showed increased levels of total and early activated (CD69^+^) CD8^+^ T cells following anti-PD-1 treatment, CDA overexpression completely abrogated this CD8^+^ T cell response to anti-PD-1 therapy (Fig. 4f).

Overall, CDA induction in cancer cells mediates anti-PD-1 resistance in different tumor types, such as PDAC, melanoma and colorectal cancer.

### CDA targeting engages the immune system against the tumor

Based on the modified immune landscape after tweaking CDA expression in cancer cells and comparable tumor growth of sgNT and sgCda Panc02 cancer cells in immunodeficient (nude) mice (Extended Data Fig. 4j,k), we tested the contribution of CD8^+^ T cells and macrophages after CDA targeting. To this end, we performed CD8^+^ T cell depletion and macrophage adoptive transfer experiments, respectively.

First, we depleted CD8^+^ T cells in sgNT and sgCda KPC FC1245 tumor-bearing mice all treated with anti-PD-1 therapy (Extended Data Fig. 4l). CD8^+^ T cell depletion rescued the growth of sgCda tumors to the level of control (sgNT) tumors; in mice treated with depleting anti-CD8, sgNT tumors were slightly bigger than their counterparts in nondepleted mice (Fig. 4g).

We then co-injected TAM-like macrophages (herein TAMs-L, which are bone marrow-derived macrophages (BMDMs) conditioned for 18 h with KPC FC1245 tumor-conditioned medium^30^) with sgNT or sgCda KPC FC1245 cells, as described previously^31^. Nine days after orthotopic injections, mice were treated with anti-PD-1. Adoptive transfer of TAMs-L in sgCda tumors was sufficient to abolish their growth defect, whereas no changes were observed after TAMs-L co-injection in sgNT tumors (Fig. 4h). Again, CDA depletion increased cytotoxic CD8^+^ T cell infiltration at the core without affecting their abundance at the tumor rim. TAMs-L co-injection completely abrogated this effect, leading to a reduction of cytotoxic CD8^+^ T cells in the tumor core (Fig. 4i). These data suggest that CDA-depleted cancer cells somehow lose their capacity to recruit macrophages and sustain their immunosuppressive phenotype.

Killing of ovalbumin (OVA)-expressing cancer cells by antigen-specific CD8^+^ T (OT-I) cells, major histocompatibility complex class I (MHC class I) exposed to the cell membrane and antigen presentation in OVA-expressing cancer cells (both at baseline and after interferon-γ (IFNγ) stimulation) were all not affected by *Cda* deletion (Extended Data Fig. 4m–o). However, in the presence of BMDMs and OVA^+^ sgCda cancer cells, OT-I T cells were activated more efficiently than when added to cocultures of BMDMs and OVA^+^ sgNT cancer cells. By contrast, coculture of sgNT or sgCda cancer cells only with OT-I T cells did not change their activation (Fig. 4j).

These data argue that CDA depletion in cancer cells sensitizes tumors to immunotherapy, possibly by defeating immunosuppressive TAMs and imposing their switch toward an immunostimulatory phenotype.

### CDA targeting limits the release of uracil nucleotides

**Fig. 5:**
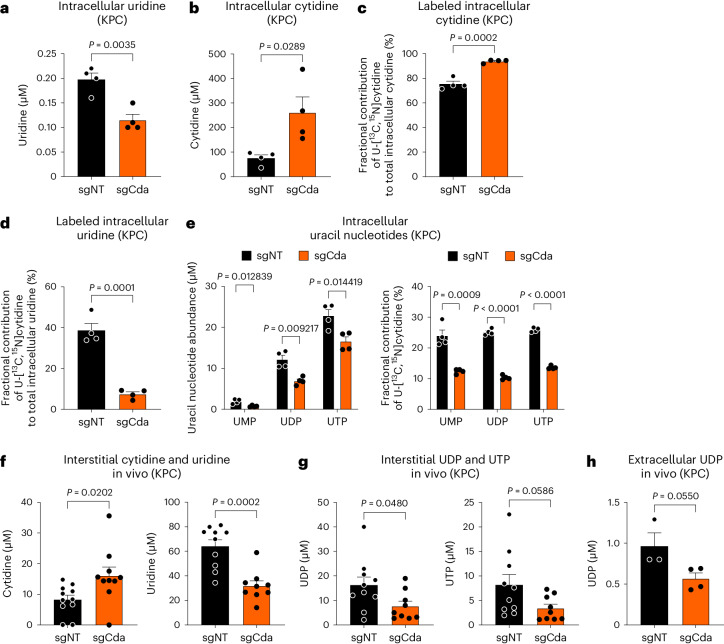
CDA regulates uracil nucleotide production by cancer cells. **a**,**b**, Intracellular abundance of total uridine (**a**) and cytidine (**b**) in sgNT and sgCda KPC FC1245 cells (sgNT *n* = 4 and sgCda *n* = 4). **c**,**d**, Fractional contribution (percentage of labeled metabolite out of total amount) of supplemented 0.1 mM [^13^C_9_,^15^N_3_]cytidine to the intracellular cytidine (**c**) and uridine (**d**) pools in sgNT and sgCda KPC FC1245 cells (sgNT *n* = 4 and sgCda *n* = 4). **e**, Left, intracellular levels of uracil nucleotides (UMP, UDP and UTP) in sgNT and sgCda KPC FC1245 cells. Right, fractional contribution of supplemented 0.1 mM [^13^C_9_,^15^N_3_]cytidine to the intracellular uracil nucleotide pools (sgNT *n* = 4 and sgCda *n* = 4). **f**, Extracellular levels of cytidine (left) and uridine (right) in the interstitial fluid of orthotopic sgNT and sgCda KPC FC1245 tumors (sgNT *n* = 11 and sgCda *n* = 10 (left); sgNT *n* = 10 and sgCda *n* = 9 (right)). **g**, Extracellular levels of UDP (left) and UTP (right) in the interstitial fluid of orthotopic sgNT and sgCda KPC FC1245 tumors (sgNT *n* = 10 and sgCda *n* = 9). **h**, Extracellular levels of UDP in the culture medium of sgNT and sgCda KPC FC1245 cells (sgNT *n* = 3 and sgCda *n* = 4). In **a**–**e** and **h**, *n* represents independently collected cell seedings. In **f** and **g**, *n* represents biological replicates. In **a**–**g**, cytidine, uridine and uracil-containing nucleotides were measured by using LC–MS. In **h**, UDP in the culture medium was measured by using enzyme-linked immunosorbent assays. In **a**–**e**, KPC FC1245 cells were cultured in DMEM supplemented with 10% dialyzed FBS (to remove the naturally present cytidine) and 0.1 mM [^13^C_9_,^15^N_3_]cytidine. In **h**, KPC FC1245 cells were cultured in DMEM supplemented with 10% dialyzed FBS (to remove the naturally present cytidine) and 0.1 mM unlabeled cytidine. Data were analyzed by unpaired, two-tailed Student’s *t*-tests (**a**–**d** and **f**–**h**) or multiple unpaired, two-tailed Student’s *t*-tests (**e**). Data are shown as mean ± s.e.m. Source data

**Extended Data Fig. 5:**
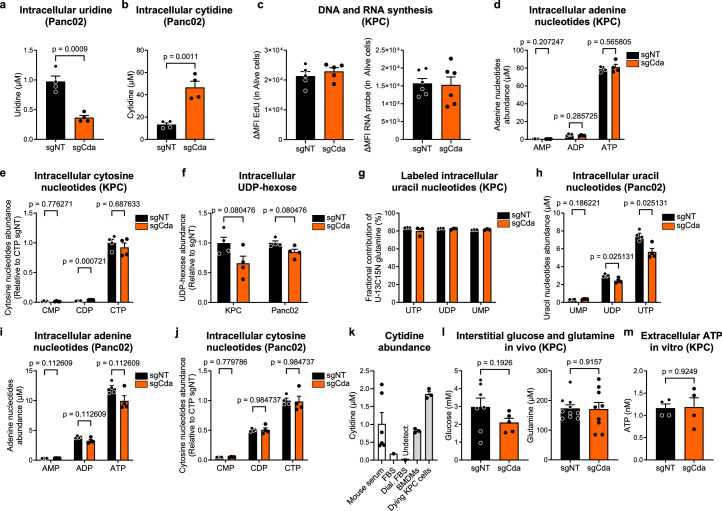
Production of various nucleotides and carbon tracing in cancer cells upon CDA depletion. (**a-b**) Intracellular abundance of total (a) uridine and (b) cytidine in sgNT and sgCda Panc02 cells. sgNT n=4 and sgCda n=4. (c) DNA and RNA synthesis of sgNT and sgCda KPC FC1245 cells. DNA, n=5; RNA n=6. (**d-e**) (d) Intracellular levels of adenine (AMP, ADP and ATP) and (e) cytosine (CMP, CDP and CTP) nucleotides in sgNT and sgCda KPC FC1245 cells. sgNT n=4 and sgCda n=4. (**f**) UDP-hexose species (UDP-glucose and UDP-galactose) in sgNT and sgCda KPC FC1245 and Panc02 cells. sgNT n=4 and sgCda n=4. (**g**) Fractional contribution (percentage of labelled metabolite out of total amount) of supplemented ^13^C_5_,^15^N_2_ glutamine to the intracellular uracil nucleotide pools (UMP, UDP and UTP) in sgNT and sgCda KPC FC1245 cells. sgNT n=3 and sgCda n=3. (**h-j**) (h) Intracellular levels of uracil nucleotides, (i) adenine nucleotides and (j) cytosine nucleotides in sgNT and sgCda Panc02. sgNT n=4 and sgCda n=4. (**k**) Extracellular levels of cytidine in mouse serum (tumor-free), standard or dialyzed fetal bovine serum (FBS and Dial. FBS), culture medium of bone marrow-derived macrophages (BMDMs) and (staurosporine-treated) dying KPC FC1245 cancer cells. Mouse serum n=6 (n represents biological replicates), FBS n=1, Dial. FBS n=1, BMDMs n=3, dying KPC cells n=3. (**l**) Extracellular levels of (left) glucose and (right) glutamine in the interstitial fluid of orthotopic sgNT and sgCda KPC FC1245 tumors. (left) sgNT n=7 and sgCda n=5; (right) sgNT n=10 and sgCda n=9. (**m**) Extracellular levels of ATP in the culture medium of sgNT and sgCda KPC FC1245 cells. sgNT n=4 and sgCda n=4. (4a-j, l, m) n represents independently collected cell seedings. (a and b, d-l) cytidine, uridine, UDP-hexose, uracil-, adenine- and cytosine-containing nucleotides, glucose, and glutamine were measured by employing liquid chromatography-mass spectrometry. (m) ATP in the culture medium was measured by employing ELISA assay. Cancer cells were cultured in DMEM medium supplemented with 10% dialyzed FBS (to remove the naturally present cytidine) and (a, b, f, h-j) 0.1 mM unlabeled cytidine or (d-f) 0.1 mM ^13^C_9_,^15^N_3_ cytidine. (g) KPC FC1245 were cultured in DMEM medium supplemented with 10% dialyzed FBS, 0.1 mM unlabeled cytidine and 2 mM ^13^C_5_,^15^N_2_ glutamine. Statistical analysis: p value was assessed by unpaired, two-tailed Student’s t-test (a-c, l and m), multiple unpaired Student’s t-test (d-j). Graphs show mean ± SEM.

By using a supraphysiological concentration of [^13^C_9_,^15^N_3_]-labeled cytidine (that is, 100 µM), we observed a decrease in intracellular uridine levels, which was mirrored by the accumulation of intracellular cytidine in sgCda versus sgNT cells (both KPC and Panc02; Fig. 5a,b and Extended Data Fig. 5a,b). In line with a reduced deamination of cytidine into uridine, intracellular abundance of [^13^C_9_,^15^N_3_]cytidine remained higher in sgCda KPC cells than in CDA-proficient control cells (Fig. 5c), with a concomitant reduction of uridine production (Fig. 5d). Consistent with the decrease in intracellular uridine, sgCda KPC FC1245 cells showed reduced intracellular levels of uracil nucleotides (UMP, UDP and UTP) compared to sgNT cells (Fig. 5e). The fraction of cytidine contributing to the uracil nucleotide pool is likely not reflecting physiology because here a supraphysiological cytidine concentration has been added to the culture medium. However, although the relative contribution of extracellular cytidine to the uracil nucleotide pool will depend on the concentration of cytidine used and the amount of extracellular uridine present, our labeling experiment highlights how CDA could take part in the generation of this pool. This difference in uracil nucleotides in sgCda versus sgNT KPC FC1245 cells did not affect DNA or RNA synthesis (Extended Data Fig. 5c). No major changes in adenine and cytosine nucleotides (that is, AMP, ADP and ATP and CMP, CDP and CTP, respectively) or in UDP-hexose (Extended Data Fig. 5d–f) were observed. Also, no changes were observed in [^13^C_5_,^15^N_2_]glutamine contribution to uracil-containing nucleotides after CDA depletion, suggesting that the de novo pyrimidine nucleotide synthesis rate was not affected (Extended Data Fig. 5g). We also confirmed a reduction in intracellular uracil nucleotide levels in sgCda Panc02 cells, without any differences in adenine and cytosine nucleotides or in UDP-hexose (Extended Data Fig. 5h–j). In general, cytidine (that is, the substrate of CDA) was found in mouse serum (that is, ~1 µM; Extended Data Fig. 5k) and in the tumor interstitial fluid (TIF; that is, ~10 µM; Fig. 5f; as previously reported^32^) and also in vitro in absolute fetal bovine serum (FBS) and in the culture medium of both macrophages and dying KPC FC1245 cells (Extended Data Fig. 5k). Liquid chromatography–mass spectrometry (LC–MS) analysis of TIFs showed that more cytidine and less uridine was found in sgCda tumors than in sgNT tumors (Fig. 5f). Glucose and glutamine levels were the same (Extended Data Fig. 5l).

Because uracil nucleotides are important signaling molecules that activate G-protein-coupled membrane receptors of the P2Y family^33^, we hypothesized that the release of UTP and UDP by cancer cells is a determinant factor in CDA-dependent resistance to anti-PD-1 therapy. First, we measured UDP and UTP levels in the extracellular milieu of sgCda and sgNT KPC FC1245 tumors. CDA depletion resulted in reduced UDP (and UTP) in the TIF (Fig. 5g). By using a protocol to assess extracellular nucleotide release in response to stress conditions^34^, we found that UDP (but not ATP) was also decreased in the culture medium of sgCda versus sgNT cancer cells (Fig. 5h and Extended Data Fig. 5m).

These results suggest that, in cancer cells, CDA is engaged in a pathway leading to the synthesis and release of uracil nucleotides.

### CDA-expressing cancer cells recruit P2Y_6_^+^ macrophages

**Fig. 6:**
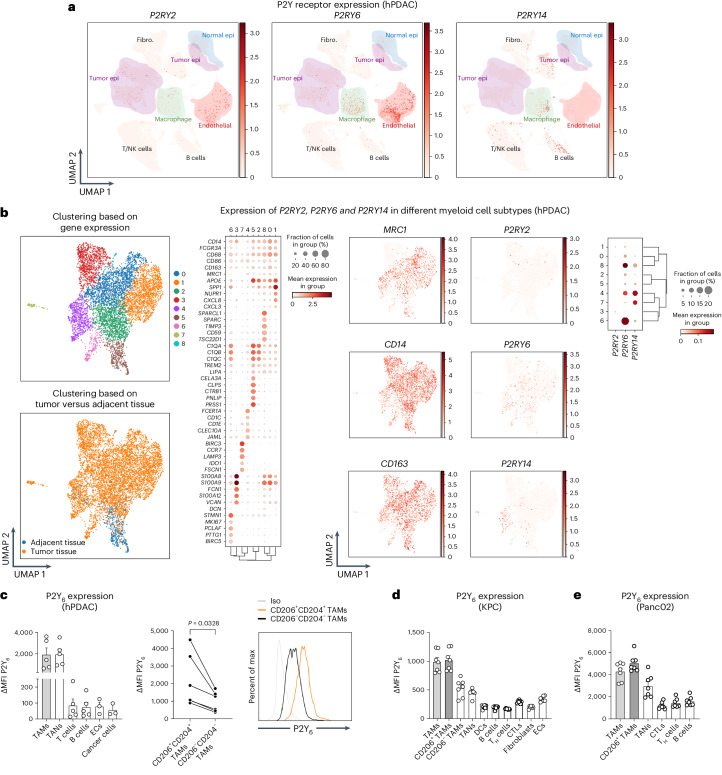
Expression profile of the UDP cognate receptor P2Y_6_. **a**, UMAP of *P2RY2*, *P2RY6* and *P2RY14* expression in different cell populations of pancreatic tissue from treatment-naive individuals with PDAC (*n* = 24). The number of cells analyzed is 83,960. **b**, Subclustering of myeloid cells/macrophages in single-cell data of human PDAC. Left, cluster analysis based on gene expression (top) or primary tumor versus adjacent tissue (bottom). The dot plot on the right shows the expression of some myeloid genes in the different clusters. Middle, UMAP of the anti-inflammatory gene *MRC1* (CD206) or the pan-myeloid markers *CD14* and *CD163* (left column) and UMAP of *P2RY2*, *P2RY6* and *P2RY14* (right column). Right, dot plot showing *P2RY2*, *P2RY6* and *P2RY14* expression across the different myeloid cell subclusters. *P2RY4* was undetectable. The number of cells analyzed is 6,482. **c**, Left, flow cytometric quantification of P2Y_6_ expression (ΔMFI, that is, MFI of P2Y_6_ minus MFI of the fluorescence minus one (FMO)) in different cell populations of human PDAC samples. Middle, flow cytometric quantification of P2Y_6_ expression (ΔMFI P2Y_6_) in paired M1-like (CD206^–^CD204^–^) and M2-like (CD206^+^CD204^+^) TAMs in human PDAC tumors. Right, representative histogram of P2Y_6_ expression (ΔMFI P2Y_6_) in TAM subsets; Iso, isotype control; % max, each curve was scaled to mode = 100% (ECs and cancer cells, *n* = 3 (left); all others *n* = 5; *n* = 6 (right)). **d**,**e**, Flow cytometric quantification of P2Y_6_ expression (ΔMFI P2Y_6_) in different cell populations from orthotopic KPC FC1245 (**d**) and s.c. Panc02 (**e**) tumors (*n* = 7). In **c**, *n* represents the number of individuals. In **d** and **e**, *n* represents the number of biological replicates. Data were analyzed by paired, two-tailed Student’s *t*-tests (**c**, middle) and are shown as mean ± s.e.m. Source data

**Extended Data Fig. 6:**
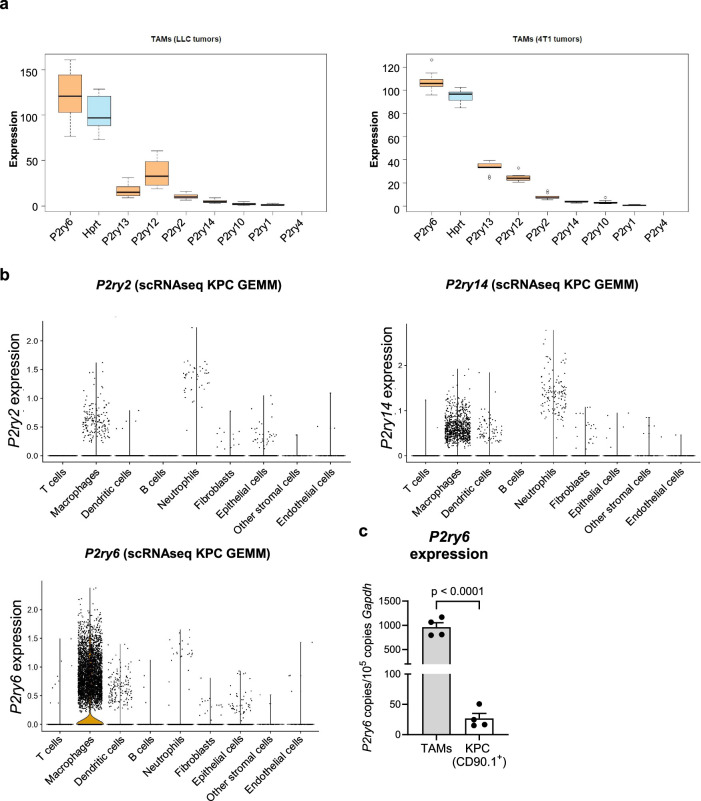
P2Y receptors in tumor-associated macrophages (TAMs). (**a**) Expression of P2Y receptor family members in TAMs of murine (left, E-MTAB-5032) LLC and (right, GSE126722) 4T1 tumors. In box plot, boxes correspond to the 25^th^ and 75^th^ quartiles, horizontal lines to the median, and whiskers extend to 1.5 times the interquartile range, with outliers shown by dots. LLC n= 16, 4T1 n= 10. (**b**) (left) *P2ry2*, (middle) *P2ry6* and (right) *P2ry14* expression in different cell populations in mouse PDAC (KPC GEMM) from a publicly available scRNA-seq dataset (GSE129455). n=4. n represents biological replicates. (**c**) *P2ry6* expression in TAMs and CD90.1^+^ cancer cells sorted from orthotopic KPC FC1245 tumors. n=4. n represents biological replicates. Statistical analysis: p value was assessed by unpaired, two-tailed Student’s t-test (c). Graphs show mean ± SEM.

P2Y_2_, P2Y_4_, P2Y_6_ and P2Y_14_ of the P2Y receptor family are pyrimidine-selective receptors that can be activated by UDP, UTP or, in the case of P2Y_14_, UDP-glucose^35–37^. Analysis of publicly available human PDAC scRNA-seq data^21^ revealed that *P2RY2* was weakly expressed in tumor epithelial cells and ECs, *P2RY4* was not detectable, *P2RY6* was strongly expressed in macrophages and ECs, and *P2YR14* expression was restricted to fibroblasts, T and B cells and a small subset of macrophages (Fig. 6a). Uniform manifold approximation and projection (UMAP) of different myeloid cell/macrophage subclusters showed that *P2RY6* expression is the most widespread among the different macrophage clusters and is completely absent in MRC1^–^ (CD206^–^) proinflammatory macrophages (cluster 3; Fig. 6b). Conversely, *P2RY14* expression was mainly detected in monocyte-derived DC1 (cluster 7) and DC3 (cluster 4) only (Fig. 6b). Dot plots of *P2RY2*, *P2RY6* and *P2RY14* expression in subclustered myeloid cells/macrophages revealed the strongest expression for *P2RY6* (Fig. 6b). Comparable results were found in TAMs from different mouse tumor types^30,38^ and in publicly available scRNA-seq data from autochthonous tumors in KPC mice^11^, showing a strong expression of *P2ry6* and weak expression of *P2ry14* and *P2ry2* in macrophages (Extended Data Fig. 6a,b).

Furthermore, flow cytometric analysis of tumor-infiltrating CD45^+^ immune cells in samples from individuals with PDAC and in both KPC FC1245 and Panc02 tumors displayed P2Y_6_ expression in TAMs (mostly in the CD206^+^ subset) and tumor-infiltrating neutrophils (TANs; Fig. 6c–e). Nonmyeloid cells and cancer cells in both human and mouse PDACs had little to no P2Y_6_ expression (Fig. 6c–e and Extended Data Fig. 6c).

**Extended Data Fig. 7:**
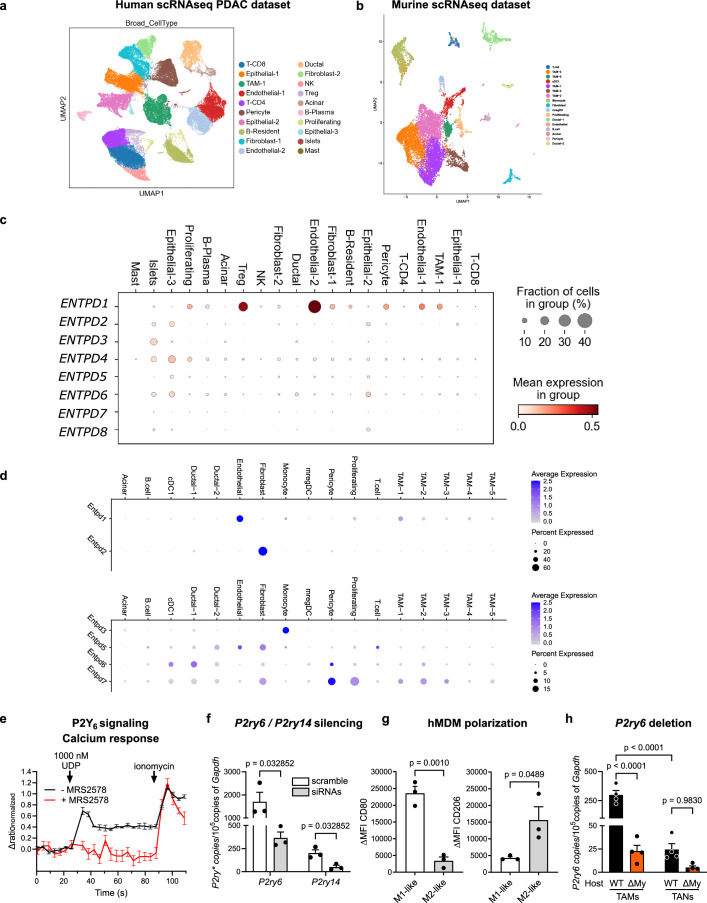
Expression of ectonucleotidases and P2Y6 in PDAC patients and mouse model. (**a-d**) Uniform manifold approximation and projection (UMAP) representing the different cell clusters of PDAC (a) patients or (b) mice, extracted from publicly available scRNAseq datasets (GSA:CRA001160 and GSE129455). Number of cells analyzed is (a) 83960 and (b) 11236. (c-d) Differential expression gene analysis (DEGs) of different ectonucleotidases across the different cell clusters in both PDAC (c) patients and (d) mice. (**e**) Time course of the changes in the fluorescence ratio (F340/F380) of the calcium indicator Fura-2 in P2ry6^WT^ bone marrow-derived macrophages (BMDMs) in response to 1000 nM of UDP with or without MRS2578 (10 µM). Responses were normalized to the response to the calcium ionophore ionomycin (2 µM). n=3. n represents independently collected cell seedings. (**f**) *P2ry6* and *P2ry14* expression in BMDMs after siRNA-mediated silencing by siP2ry6 or siP2ry14 and scramble controls. n=3. n represents independently collected cell seedings. (**g**) Flow cytometric quantification of CD80 and CD206 expression (ΔMFI) in human M1-like (IFN-y + LPS) and M2-like (IL-4) in vitro polarized monocyte-derived macrophages (hMDMs). n=3. n represents independently collected cell seedings. (**h**) *P2ry6* expression in TAMs and tumor-associated neutrophils (TANs) sorted from orthotopic KPC FC1245 tumors in P2ry6^WT^ and P2ry6^ΔMy^ mice. n=4. n represents biological replicates. Statistical analysis: p value was assessed by unpaired, two-tailed Student’s t-test (c and f), multiple unpaired, two-tailed Student’s t-test (d), two-way ANOVA with Sidak’s multiple comparison test (g). Graphs show mean ± SEM.

P2Y_6_ is a high-affinity receptor for UDP, and it is weakly responsive to UTP^33,39^. Once released or leaked in the extracellular milieu^40^, UTP is converted to UDP by ectonucleotidases. In both human and mouse PDAC scRNA-seq datasets, ectonucleotidases (for example, *ENTPD1*, *ENTPD2* and *ENTPD3*) were found to be abundantly expressed in ECs, fibroblasts and immune cells (Extended Data Fig. 7a–c). Therefore, released UDP, or UTP-derived UDP, can activate P2Y_6_.

**Fig. 7:**
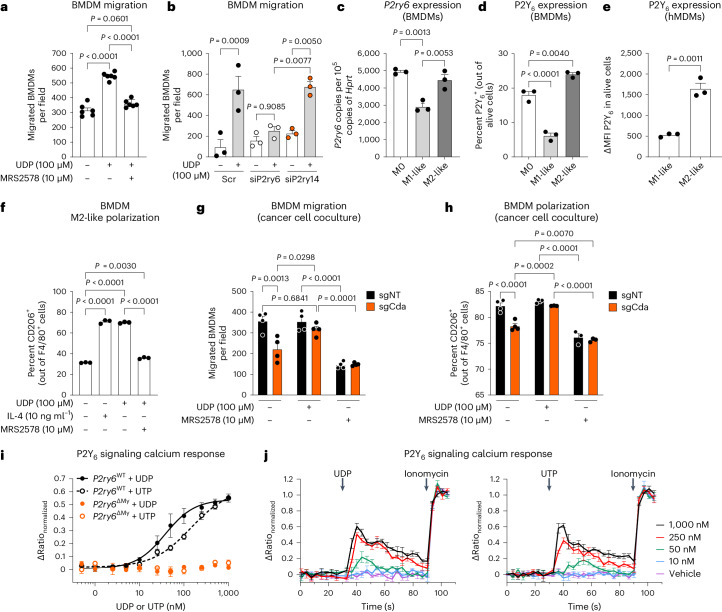
CDA–UDP–P2Y_6_ axis shapes macrophage phenotype. **a**, BMDM migration in response to UDP (100 µM), MRS2578 (10 µM), UDP + MRS2578 or nothing (basal; *n* = 6). **b**, Migration of P2Y_6_- or P2Y_14_-silenced BMDMs in the presence or absence of UDP (*n* = 3); Scr, scrambled. **c**, *P2ry6* expression in M0 (unstimulated), M1-like (IFNγ + LPS) and M2-like (IL-4) polarized BMDMs (*n* = 3). **d**, Flow cytometric quantification of P2Y_6_ expression (percentage of P2Y_6_^+^ cells out of alive) in M0, M1-like and M2-like polarized BMDMs (*n* = 3). **e**, Flow cytometric quantification of P2Y_6_ expression (ΔMFI P2Y_6_ in alive cells) in M1-like (IFNγ + LPS) and M2-like (IL-4) polarized hMDMs (*n* = 3). **f**, Flow cytometric quantification of M2-like BMDMs (percentage of CD206^+^ out of F4/80^+^ cells) after stimulation with IL-4, UDP, MRS2578 or nothing (basal; *n* = 3). **g**, BMDM migration in the presence of sgNT or sgCda Panc02 cells supplemented with UDP (bottom chamber), MRS2578 (top chamber) or nothing (basal; *n* = 4). **h**, Flow cytometric quantification of M2-like BMDMs (percentage of CD206^+^ out of F4/80^+^ cells) cocultured with sgNT or sgCda Panc02 cells in the presence of UDP or MRS2578 (*n* = 3–4). **i**, Concentration–response curves for the peak increase in fluorescence ratio in response to UDP or UTP. Solid lines represent best fit with the Hill function. Half-maximal effective concentration (EC_50_) values were 42 ± 5 nM for UDP and 103 ± 12 nM for UTP in wild-type P2Y_6_ (*P2y6*^WT^) BMDMs (UDP *n* = 3; UTP *n* = 6). **j**, Time course of the changes in the fluorescence ratio (*F*_340_/*F*_380_) of the calcium indicator Fura-2 in *P2y6*^WT^ BMDMs in response to the indicated concentrations of UDP (left) or UTP (right). Responses were normalized to the response to the calcium ionophore ionomycin (2 µM; UDP *n* = 3; UTP *n* = 6). In **a**–**j**, *n* represents independently collected cell seedings. Data were analyzed by one-way ANOVA with Tukey’s multiple comparison test (**a**–**d** and **f**), unpaired, two-tailed Student’s *t*-tests (**e**) or two-way ANOVA with a Tukey’s multiple comparison test (**g** and **h**). Data are shown as mean ± s.e.m. Source data

We then treated BMDMs with 100 µM UDP, based on previous studies^40,41^, and proved the chemotactic potential of UDP; this effect was inhibited by the P2Y_6_ antagonist MRS2578 (ref. ^42^; Fig. 7a). MRS2578 also fully abrogated the UDP-evoked intracellular calcium response (Extended Data Fig. 7d). As TAMs (Extended Data Fig. 6a,b), BMDMs expressed tenfold more *P2ry6* than *P2ry14*, and *P2ry6* silencing only completely prevented macrophage migration toward UDP (Fig. 7b and Extended Data Fig. 7e). We then observed that at both RNA and protein levels, P2Y_6_ expression was the highest in M0 or M2-like (that is, stimulated with interleukin-4 (IL-4)) BMDMs and was decreased in M1-like (that is, stimulated with lipopolysaccharide (LPS) and IFNγ) BMDMs (Fig. 7c,d). The same was true in human monocyte-derived macrophages (hMDMs), where M2-like polarization (that is, stimulation with IL-4) resulted in higher P2Y_6_ expression than M1-like hMDMs (stimulated with LPS and IFNγ; Fig. 7e), correlating with reduced expression of immunostimulatory CD80 and increased CD206 levels (Extended Data Fig. 7f). Like IL-4, UDP induced CD206 expression in BMDMs that was inhibited by MRS2578 (Fig. 7f).

Third, BMDM migration toward sgCda cells was lower than toward sgNT cells, and UDP supplementation rescued this defect while MRS2578 reduced macrophage migration toward sgNT cancer cells (Fig. 7g). Similarly, coculture of sgCda cells with BMDMs resulted in a reduced percentage of CD206^+^ macrophages, which was rescued by adding UDP to the medium, whereas P2Y_6_ inhibition reduced the percentage of CD206^+^ macrophages in cocultures with sgNT cells (Fig. 7h). These data argue that cancer cells recruit P2Y_6_^+^ macrophages and sustain their immunosuppression activity via UDP (and UTP) release.

Finally, we deleted P2Y_6_ in the myeloid cell-specific lineage by intercrossing *P2ry6*^*loxP*/*loxP*^ mice with *LysM-cre* mice (herein *P2ry6*^ΔMy^ and the related wild-type control *P2ry6*^WT^). *P2ry6* levels were again found to be much higher in TAMs than in TANs, and deletion of *P2ry6* was almost complete in sorted TAMs from tumor-bearing *P2ry6*^ΔMy^ mice and less efficient in TANs (Extended Data Fig. 7g). When measuring intracellular calcium release^42^ at different concentrations of UDP or UTP, we observed concentration-dependent calcium responses to both UDP and UTP in *P2ry6*^WT^ but not *P2ry6*^ΔMy^ BMDMs (Fig. 7i,j).

**Fig. 8:**
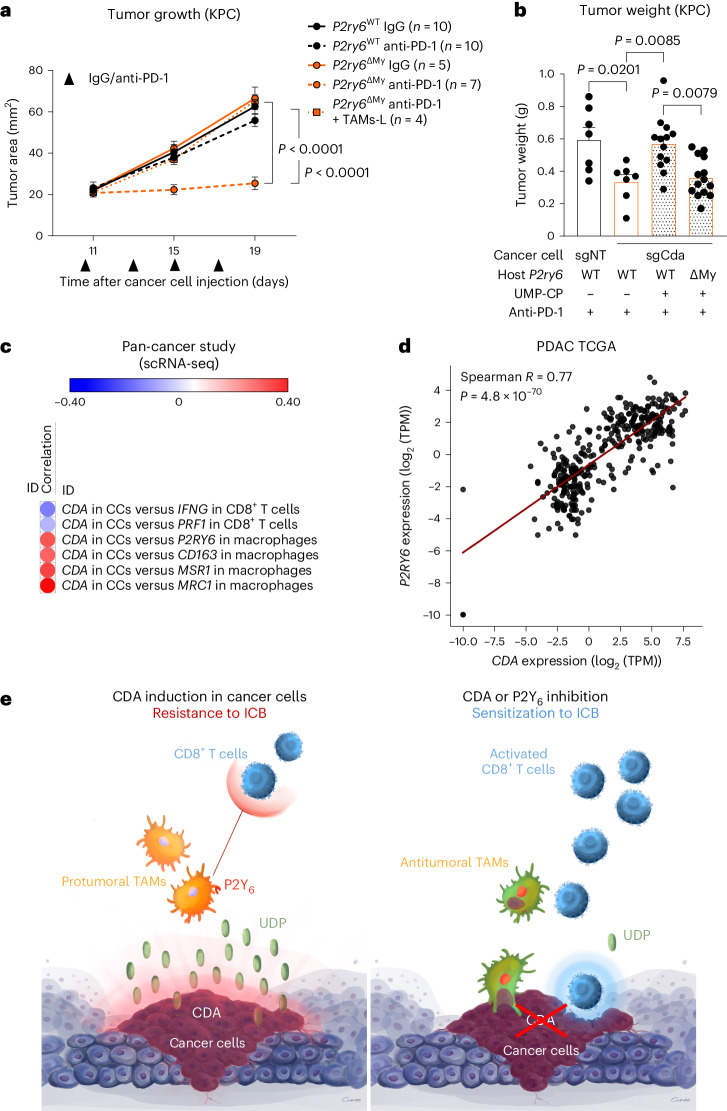
CDA- and UDP-dependent anti-PD-1 resistance is executed by macrophages via the P2Y_6_ receptor. **a**, Longitudinal measurements of tumor area by ultrasound imaging in KPC FC1245 tumor-bearing *P2y6*^WT^ and *P2y6*^ΔMy^ mice treated with anti-PD-1 or control IgG (*P2y6*^WT^ (IgG) *n* = 10, *P2y6*^WT^ (anti-PD-1) *n* = 10, *P2y6*^ΔMy^ (IgG) *n* = 5, *P2y6*^ΔMy^ (anti-PD-1) *n* = 7, *P2y6*^ΔMy^ + TAMs-L (anti-PD-1) *n* = 4). Treatment regimen is indicated by the black arrowheads. **b**, Weight of sgNT and sgCda KPC FC1245 tumors in *P2y6*^WT^ and *P2y6*^ΔMy^ mice treated i.p. with UMP-CP (10 mg per kg (body weight)) or PBS and cotreated with anti-PD-1 (sgNT *P2y6*^WT^ (PBS) *n* = 7, sgCda *P2y6*^WT^ (PBS) *n* = 7, sgCda *P2y6*^WT^ (UMP-CP) *n* = 13, sgCda *P2y6*^ΔMy^ (UMP-CP) *n* = 14). Data are representative of a pool of two independent experiments. **c**, Correlation between *CDA* expression in cancer cells (CCs) versus *IFNG* or *PRF1* expression in CD8^+^ T cells or *P2RY6*, *CD163*, *MSR1* or *MRC1* expression in macrophages at single-cell resolution in 11 diverse cancer types. **d**, Spearman correlation analysis between *P2RY6* and *CDA* expression in individuals with PDAC from TCGA (*n* = 177); TPM, transcripts per million. **e**, Scheme of the contribution of CDA and P2Y_6_^+^ macrophages to immunotherapy resistance. Induction of CDA expression in pancreatic cancer cells contributes to the production and release of uracil nucleotides. Released UDP, as well as UTP-derived UDP, binds with high affinity to the cognate receptor P2Y_6_ expressed by TAMs, therefore fostering their recruitment and immunosuppressive features. This ultimately shields the tumor from the entry and activation of cytotoxic T cells, a condition that renders the tumor refractory to anti-PD-1 treatment. Inhibition of CDA or P2Y_6_ breaks this cross-talk between cancer cells and TAMs by decreasing the amount of UDP in the TME. It follows that tumors are less infiltrated by immunosuppressive TAMs, and their phenotype is now more immunostimulatory, altogether favoring (instead of preventing) the recruitment and activation of cytotoxic T cells in response to anti-PD-1 treatment. Under this condition, resistant tumors are sensitized to anti-PD-1 therapy, displaying reduced primary growth and metastatic dissemination. In **a** and **b**, *n* represents biological replicates. Data were analyzed by two-way repeated measures ANOVA (**a**), two-way ANOVA with a Tukey’s multiple comparison test (**b**) and two-sided Spearman’s test (**c**). Data are shown as mean ± s.e.m. Source data

We then performed in vivo experiments where anti-PD-1 was able to decrease orthotopic KPC FC1245 tumor area in *P2ry6*^ΔMy^ mice only (Fig. 8a). Transfer of wild-type macrophages to tumor-bearing *P2ry6*^ΔMy^ mice was sufficient to abrogate the inhibitory effect of myeloid cell-specific *P2y6* deletion on tumor growth (Fig. 8a), highlighting P2Y_6_ in macrophages as a key regulator of ICB resistance.

We then assessed in vivo whether supplementation with exogenous UDP could re-establish resistance to anti-PD-1 therapy in sgCda tumors. Indeed, systemic (intraperitoneal (i.p.)) injection of uridine-5′-*O*-α,β-methylene-diphosphate (UMP-CP; a hydrolytically stable analog of UDP) in anti-PD-1-treated sgCda KPC FC1245 tumor-bearing mice restored tumor weight to the same level as observed in anti-PD-1-treated sgNT tumor-bearing mice (Fig. 8b). *P2ry6* deletion in myeloid cells completely abrogated this effect (Fig. 8b).

To corroborate the mechanistic findings in human datasets, we aimed to reveal the link between *CDA* expression and the immune landscape in PDAC and many other cancers. A pan-cancer study^43^ with 16 scRNA-seq datasets (including 11 different tumor types) highlighted an association between high *CDA* levels in cancer cells and a ‘cold’ immune microenvironment (that is, negative correlation with average expression of *PRF1* or *IFNG* in CD8^+^ T cells versus a positive correlation with average expression of protumoral *P2RY6*, *CD204*, *CD163* or *MRC1* in TAMs with respect to average expression of *CDA* in cancer cells across these 16 datasets; Fig. 8c). Likewise, in the PDAC TCGA dataset, *CDA* and *P2RY6* were positively correlated (Fig. 8d).

Therefore, CDA induction in cancer cells promotes immunosuppression through activation of P2Y_6_ in TAMs.

## Discussion

Here, we show that the pyrimidine salvage pathway enzyme CDA is a limiting factor to replenish the extracellular milieu with uracil nucleotides, supporting the recruitment of immunosuppressive TAMs through the activation of the UDP receptor P2Y_6_. This prevents cytotoxic T cell infiltration, proliferation and function, which leads to the failure of anti-PD-1 treatment.

Although previous approaches have successfully re-educated TAMs toward antitumoral functions^44^, here, we observed that reducing UDP levels by CDA inhibition mitigates TAM migration and their protumoral phenotype but in most cases is insufficient to achieve tumor inhibition. When breaking immune suppression, tumors rely on immune checkpoints to prevent T cell proliferation and activation^45^. As a result, targeting CDA in cancer cells or inhibiting P2Y_6_ in macrophages renders T cells susceptible to anti-PD-1 therapy. We have carefully excluded the possibility that these effects are due to neoantigens while, consistent with previous research^46^, we show that TAMs, and in particular P2Y_6_-expressing TAMs, play a crucial role in establishing a T cell-excluded tumor phenotype and contribute to immunotherapy resistance (Fig. 8e).

Prior studies have explored CDA’s ability to deactivate nucleoside analogs^17^, and CDA inhibitors have been tested to extend the half-life of the deoxycytidine analog and chemotherapeutic drug, namely gemcitabine^47–50^. Our work presents a mechanism by which cancer cells use nucleotide metabolism to mount immunosuppression and ICB resistance. Combining CDA inhibitors with both gemcitabine and anti-PD-1 therapy may have synergic effects, potentiating the cytotoxic impact of gemcitabine on cancer cells and tumor immunogenicity^51,52^ and enabling the immune system to act against the tumor in response to anti-PD-1. However, only a few P2Y_6_ antagonists have been developed and evaluated, primarily in vitro or in mice, on medical conditions such as obesity, type 2 diabetes or cardiac fibrosis^53–56^. Therefore, our study suggests the repurposing of both CDA and P2Y_6_ inhibitors in immuno-oncology.

Targeting the pyrimidine salvage pathway reduces the extracellular abundance of uracil-containing nucleotides without disrupting nucleic acid synthesis. UTP, a product of this pathway, directly serves as a substrate of the RNA polymerase and is indirectly linked to DNA synthesis through the production of deoxythymidine triphosphate. Moreover, UTP plays a crucial role as an energy carrier, participates in carbohydrate metabolism and contributes to the formation of glycoproteins and proteoglycans. In addition, a recent study has shown that PDAC relies on extracellular uridine uptake in glucose-restricted conditions, fueling carbon metabolism (through the liberation of its ribose) and contributing also to the pool of uracil nucleotides^57^. Our findings suggest that the pyrimidine salvage pathway complements de novo pyrimidine synthesis and extracellular uridine utilization, offering an additional source for uridine and uracil nucleotide production. Perturbing CDA activity leads to a decrease in the pools of uridine and uracil-containing nucleotides, which forces the cell to preserve them for its own metabolism.

Extracellularly, nucleotides can trigger immunoregulatory mechanisms that affect chemotaxis, differentiation, immune recognition and effector functions of innate and adaptive immune cells^58^. Although the role of purinergic receptors (for example, adenosine receptors) in cancers has been largely studied^58,59^, less is known on pyrimidinergic signaling. In particular, the UDP-activated metabotropic receptor P2Y_6_ has been implicated in the regulation of myeloid cells, promoting type 2 functions, hyper-reactivity and immunosuppression in contexts such as experimental asthma and dust mite allergy in mice or autoimmune Graves’ disease in humans^60–62^. In cancer, there is limited research on the UDP–P2Y_6_ axis. Only one publication to date has suggested its prometastatic role by mediating neutrophil propagation in the premetastatic niche of melanomas^63^. In our study, P2Y_6_ in macrophages establishes communication with cancer cells that sustains immunosuppression and hinders T cell recruitment. The release of nucleotides (that is, UDP and UTP) can occur actively through regulated mechanisms, such as exocytosis, channels and transporters, but greatly via nonregulated mechanisms, such as cytolysis of damaged or dying cells in response to stressors such as hypoxia or cytotoxic agents^64,65^. Our findings exclude the possibility of futile cycles where de novo pyrimidine synthesis would contribute to cytidine in the salvage pathway, suggesting that exogenous cytidine (derived from the circulation, excreted by macrophages or released by dying cells) is the primary CDA substrate^66^. Our findings argue that when cytidine is in the extracellular environment, some of it can be used for uridine synthesis and, eventually, contribute to uracil-containing nucleotides. Once outside the cancer cell, UDP (and UTP) binds to P2Y_6_, which we predominantly identified in TAMs within PDAC tumors. Disrupting this communication through CDA or P2Y_6_ inhibition leads to the infiltration of effector T cells into the tumor, transforming it into a T cell-inflamed or ‘hot’ state, a condition that enables the effectiveness of anti-PD-1 treatment. Based on the existing research on UDP–P2Y_6_ and its role in neutrophils and metastasis^63^, as well as the expression of P2Y_6_ in myeloid cells in both human and mouse PDAC, we cannot rule out that interfering with this pathway also disrupts the immunosuppressive capacity of P2Y_6_-expressing TANs.

The similarity between the cellular and molecular pathways hijacked by a tumor and those involved in tissue repair raises the possibility of a physiological function of this CDA–UDP–P2Y_6_ axis. Tissue damage might trigger the release of UDP and UTP into the extracellular environment^64,65^, potentially activating an anti-inflammatory program mediated by the recruitment of P2Y_6_-expressing macrophages. This, in turn, may halt T cell recruitment and their cytolytic activity while facilitating cell debris clearance and tissue repair, functions associated with M2-like anti-inflammatory macrophages.

From a clinical perspective, our research argues that combining CDA or P2Y_6_ inhibition with immunotherapy in PDAC could be a promising approach for treating nonresectable or borderline resectable tumors, aiming to reduce the neoplastic mass before surgery. Our results in the YUMM1.7 melanoma model also suggest a therapeutic option for individuals facing initial or successive resistance to ICB, which occurs in about 60% of treated individuals. Moreover, our bioinformatic analyses in individuals with cancer demonstrate that *CDA* levels are highest in 11 different tumor types with immunosuppressive features (that is, high in *P2RY6*, *MRC1* and *MSR1* levels in macrophages and low in *IFNG* and *PRF* levels in CD8^+^ T cells). These observations align strongly with our mechanistic findings in mice. It remains to be explored whether CDA or P2Y_6_ blockade in combination with ICB might work in all these tumor types. Because most of our experimental design relies on the use of anti-PD-1, more data are warranted to assess if these conclusions can be extended to anti-CTLA-4 and other ICB-based therapies at large.

Immunotherapy has been recommended as a second-line treatment for mismatch repair-deficient advanced PDAC^67^. However, there have been sporadic cases of partial response to immunotherapy in mismatch repair-proficient PDAC^68^. Our retrospective analysis in a published dataset of individuals with PDAC^22^, together with the initial meta-analysis in individuals with metastatic melanoma and renal cancer^3,5,69^, suggests that nearly all tumors with high CDA expression before treatment will not respond to ICB (anti-PD-1 or anti-CTLA-4 in melanoma, anti-PD-1 in renal cancer and three cycles of anti-PD-1/anti-CTLA-4 combined with radiation on cycle two in individuals with PDAC). Conversely, low CDA expression in cancer cells identifies a subgroup of individuals with PDAC with fewer immunosuppressive TAMs and more T cells who might benefit from ICB. Tailored prospective studies will provide further insights into whether the CDA status of the tumor should be considered when selecting individuals with PDAC for cancer immunotherapy.

Overall, our research uncovers how cancer cells exploit the CDA-mediated pyrimidine salvage pathway to create a TME rich in UDP (and UTP). This environment supports the infiltration and immunosuppressive features of P2Y_6_-expressing TAMs, hampering CD8^+^ T cell recruitment and activation. We provide compelling evidence that inhibiting this axis in PDAC, and other cancer types, has the potential to enhance immunotherapy.

## Methods

### Ethics statement

All experimental animal procedures were approved by the Institutional Animal Care and Research Advisory Committee of KU Leuven (ECD P226/2017 and P060/2021). All human data contained in this study were approved by the Ethical Committee of the University Hospitals KU Leuven with reference number ML3452 (related to histology and flow cytometric analyses). All participants provided informed consent. Clinical information is provided in Supplementary Table 1.

### Animals

Female mice, 8 to 10 weeks old, were maintained under pathogen-free and temperature- and humidity-controled conditions on a 12-h light/12-h dark cycle and received normal chow (ssniff R/M-H). Animals with symptoms of illness, that lost 20% of their initial body weight or with s.c./intradermal tumors that were ulcerated or bigger than 2,000 mm^3^ were killed. Therefore, the maximum permitted tumor burden in mouse experiments was not exceeded. C57BL/6 and NMRI-*Foxn1*^*nu*^ mice were purchased from Envigo. OT-I mice were purchased from Taconic. The *P2ry6* floxed mouse line (*P2ry6*^tm1Jabo^; MGI:5304911 (ref. ^62^)) in the C57BL/6 background was kindly provided by J. A. Boyce (Harvard Medical School, Boston). *P2ry6*^ΔMy^ mice were generated by intercrossing *P2ry6* floxed mice with a *LysM-cre* deleter (B6.129P2-*Lyz2*^tm1(cre)Ifo^/J, Jackson Laboratory).

### Cell lines

Panc02 cells were provided by B. Wiedenmann (Charité, Berlin) and were cultured in DMEM (Gibco) supplemented with 10% FBS (Gibco) and 1% penicillin/streptomycin (Pen/Strep; Gibco). KPC FC1245 and FC1199 cells, a gift from D. Tuveson (Cold Spring Harbor), were generated from *Kras*^LSL.G12D/+^; *Trp53*^R172H/+^; *Pdx1-cre*^tg/+^ mice and were cultured in DMEM supplemented with 10% FBS, 1 mM sodium pyruvate (Gibco) and 1% Pen/Strep (Gibco). MC38 cells were obtained from Kerafast and were cultured in DMEM supplemented with 10% FBS, 2 mM glutamine (Gibco), 0.1 mM nonessential amino acids (Gibco), 1 mM sodium pyruvate, 10 mM HEPES (Gibco) and 1% Pen/Strep. CT26 cells were purchased from ATCC and were cultured in RPMI (Gibco) supplemented with 10% FBS and 1% Pen/Strep. YUMM1.7 cells were a gift from R. Marais (Cancer Research UK, Manchester) (Sigma-Aldrich) and were cultured in DMEM/F-12 medium supplemented with 10% FBS and 1% Pen/Strep.

Cells were incubated at 37 °C in a 5% CO_2_ humidified atmosphere. All cell lines were authenticated based on morphological criteria only.

### Lentiviral knockdown and overexpression strategies

Panc02, KPC1245, KPC1199 and YUMM1.7 cells were transduced with a doxycycline-inducible Cas9 nuclease (Edit-R Inducible Lentiviral Cas9, Dharmacom), selected with blasticidin (Bio-Connect) and transduced with a vector containing an sgRNA targeting *Cda* or a control nontargeting guide RNA (Supplementary Table 2). A multiplicity of infection reaching approximately 30% of transduction was used. Transduced cells were then selected with puromycin (2 µg ml^−1^; Sigma-Aldrich), treated for 7 days with doxycycline (2.5 µg ml^−1^; Sigma-Aldrich) to induce Cas9 expression and grown for 7 more days in doxycycline-free medium before any functional assays. Single-cell clone isolation and expansion were performed by using limiting dilution cloning. A pool of six clones was used exclusively for survival analysis in mice, being a long-term in vivo experiment.

OVA expression in Panc02 cells and CDA overexpression in sgCda KPC FC1245 and MC38 cells were achieved by using a lentiviral vector with the open reading frame under the control of a cytomegalovirus promoter. Control cells were transduced with EVs. Transduced cells were then selected with puromycin (2 µg ml^−1^; Sigma-Aldrich).

Silencing and overexpression efficiency was checked by both RT–qPCR and western blotting.

### RNA extraction, cDNA synthesis and RT–qPCR

RNA was extracted with an RNeasy Minikit (Qiagen), according to the manufacturer’s instructions, quantified with a Nanodrop 2000 (Thermo Scientific) and retrotranscribed into cDNA with a QuantiTect Reverse Transcription kit (Qiagen) or SuperScript III First-Strand Synthesis System (Invitrogen), according to manufacturer’s protocol. Primer mix and PowerUp SYBR Green mix (Applied Biosystems) or TaqMan Fast Universal PCR master mix were prepared according to manufacturer’s instructions (Applied Biosystems). Analyses were performed using a QuantStudio 12K Flex Real-Time PCR System (Applied Biosystems, v1.4). The primers used are listed in Supplementary Table 2. Of note, for *Cda*, we designed an in-house protocol using primers probing the targeting region shared by both gRNAs against *Cda*.

### Protein extraction and immunoblotting

Immunoblotting on whole-cell lysates was performed as previously described^70^. The following antibodies were used: rabbit anti-mouse CDA and horseradish peroxidase (HRP)-conjugated anti-β-tubulin and appropriate HRP-conjugated secondary antibody. The signal was visualized with Enhanced Chemiluminescent reagents (Invitrogen) or SuperSignal West Femto Chemiluminescent Substrate (Thermo Scientific) with a digital imager (ImageQuant LAS 4000, GE Health Care Life Science Technologies).

### Cancer cell conditioned medium

In total, 200,000 sgNT or sgCda cells were seeded in 500 µl of complete DMEM supplemented with 10% FBS, 1% Pen/Strep and 1 mM sodium pyruvate (Gibco) in 24-well plates for 36 h, after which the supernatant was collected and filtered using a 0.22-µm filter.

### Cell growth analysis

sgNT and sgCda Panc02, KPC1245, KPC1199 and YUMM1.7 cells were seeded in six-well plates (5 × 10^4^ cells per 2 ml per well) and incubated at 37 °C in a 5% CO_2_ humidified atmosphere until cells attached (*t*_0_) or for 24 (*t*_24_), 48 (*t*_48_) or 72 h (*t*_72_). At different time points, cells were counted using a hemocytometer. The cell growth rate was defined as the number of cell divisions normalized to *t*_0_.

### RNA and DNA assays

KPC FC1245 sgNT and sgCda cells were seeded in 48-well plates (0.5 × 10^5^ cells per 250 µl per well) the day before the experiments, which was performed by using an RNA synthesis assay kit (ab228561) or a Click-iT EdU Alexa Fluor 647 Flow Cytometry Assay kit (C10634), following the manufacturer’s instructions.

Samples were then analyzed using an LSRFortessa (BD Biosciences) flow cytometer. Negative controls were used to ensure proper gating of positive cells. Data were collected and analyzed with BD FACSDiva (v9.0) and FlowJo software (v10.8.1), respectively.

### LC–MS

Cancer cells were seeded at a density of 30,000 cells per well in complete DMEM in six-well plates. The day after, the cells and empty wells (for background control) were washed with PBS and replenished with DMEM containing 10% dialyzed FBS (to remove the naturally present cytidine), 1% Pen/Strep, 5.5 mM glucose, 2 mM glutamine, 1% Pen/Strep and 0.1 mM labeled (^13^C_9_ = 98%, ^15^N_3_ = 96–98%, Eurisotop) or unlabeled cytidine (Sigma-Aldrich) for 48 h. Cells were then washed once in ice-cold saline solution (9 g l^−1^ NaCl), covered with 250 μl of precooled 80% methanol for 2 min, scraped, transferred to fresh vials and stored at −80 °C overnight. Samples were then centrifuged at 20,000*g* for 15 min at 4 °C, and the supernatant was used for analysis. The cell pellet was dissolved in 100 μl of 200 mM NaOH for 20 min at 95 °C, and the protein concentration was determined. Samples that were used to assess the de novo pyrimidine nucleotide synthesis rate were prepared by incubating cells with labeled glutamine (^13^C_5_ = 99%, ^15^N_2_ = 99%, Eurisotop) for 24 h and by following the protocol described above.

For LC–MS analysis, 10 μl of sample was loaded, and the metabolites were resolved on a Q Exactive Hybrid Quadrupole-Orbitrap mass spectrometer (Thermo Scientific).

Absolute concentrations of uridine, cytidine and both uracil and adenine nucleotides were calculated by spiking the internal standard into the sample. Absolute concentrations were normalized for protein content. All raw data are available at Metabolomics Workbench^71^ (DATATRACK ID 4162, Study ID ST002791).

### Extracellular UDP quantification

Extracellular UDP was measured following an adapted protocol from the literature^34^. Briefly, confluent monolayers of sgNT and sgCda KPC FC1245 cells were cultured in DMEM (Gibco, A1443001) supplemented with 5.5 mM glucose, 2 mM glutamine, 1% dialyzed FBS (Sigma-Aldrich), 1% Pen/Strep (Gibco) and 0.1 mM unlabeled cytidine (Sigma-Aldrich) for 6 h at 37 °C. Extracellular UDP was detected by using a MicroMolar UDP Assay kit (ProFoldin), in accordance with the manufacturer’s protocol. UDP levels were measured by detecting fluorescence using a microplate reader (Tecan), and a standard curve of UDP was used to quantify the concentration.

### Extracellular ATP quantification

sgNT and sgCda KPC FC1245 cells were seeded (120,000 cells per well) in 1.4 ml of complete DMEM for 48 h at 37 °C. The medium was collected and centrifuged at 16,000*g* at 4 °C for 15 min. The supernatant was collected and stored at −80 °C. ATP was then measured with a luminescent ATP Detection Assay kit (Abcam, 113849), in accordance with the manufacturer’s protocol. Luminescence was detected using a microplate reader (Tecan). An ATP standard curve was used to quantify the concentration.

### Tumor models

Panc02 (4 × 10^6^), MC38 (1 × 10^6^) and CT26 (2 × 10^6^) cells were injected s.c. into the right flank of mice in 200 μl of PBS. YUMM1.7 cells (1 × 10^6^) were injected intradermally in 50 μl of PBS. Tumor volumes were measured at least three times per week. FC1245 (0.4 × 10^5^) or FC1199 cells (0.15 × 10^6^; referred to as KPC cells) were injected orthotopically into the pancreas (head) in 20 μl of PBS. Alternatively, in the adoptive transfer experiment, 0.4 × 10^5^ KPC FC1245 cells were resuspended together with 0.8 × 10^5^ TAMs-L^30^ and injected orthotopically into the pancreas (head) in 20 μl of PBS.

At the indicated time points, mice were randomized and treated i.p. with 10 mg per kg (body weight) Ultra-LEAF Purified anti-PD-1 (BioLegend, 96167, RMP1-14), 5 mg per kg (body weight) anti-CD8 (BioXcell) or control IgG from rat serum (Sigma-Aldrich). For the UMP-CP experiment, mice were injected i.p. with 10 mg per kg (body weight) UMP-CP (BIOLOG, U009-05) daily or control vehicle (PBS) twice daily. Treatment started at day 5 after cancer cell injection and continued until day 11 after injection. In CDZ experiments, mice were treated by oral gavage with 30 mg per kg (body weight) CDZ (DC Chemicals, DC20978) or vehicle (drinking water) daily starting at day 4 after cancer cell injection until the end of the experiment.

Mice were monitored continuously during the experiments. At the indicated time points, tumor area was assessed via ultrasound imaging. At end stage (20 days after cancer cell injection), tumor weight was registered, and samples were collected. Moreover, in the orthotopic KPC FC1245 and FC1199 models, metastatic mesenteric lymph nodes were assessed.

### Ultrasound

An ultrasound was performed using a Vevo3100 (Vevo Lab 5.7.1) from VisualSonics. A transducer with central frequency at 40 MHz, gain at 30 dB and 13-mm depth was used for imaging the tumors using B Mode at 100% transmit power. Mice were anesthetized using 2% isofluorane at approximately 2 l min^−1^, and hair was removed over the abdomen. Body temperature was monitored and kept within 37 °C ± 1 °C using a heat lamp. Ultrasound gel was used. Acquired images at the indicated time points were analyzed using the VisualSonics imaging software package. Two measurements of the largest diameters of each tumor were recorded. To maintain consistency and reliability of the experimental data, measurements of mice with prominent scar tissue (growing at the surgical site and connected to the pancreatic tumor) were not included.

### Tumor-conditioned medium

KPC FC1245 sgNT tumors explanted from wild-type mice were minced in 12 ml of DMEM supplemented with 1% Pen/Strep (FBS free) and incubated at 37 °C for 72 h. The medium was then filtered, and the cell-free supernatant was supplemented with 20 mM HEPES and 2 mM l-glutamine and stored at −20 °C.

### TIF

Tumor-bearing mice were killed with 75 µl of a 60 mg ml^−1^ Dolethal solution (pentobarbital sodium, Vetoquinol). Subsequently, sgNT and sgCda primary tumors were collected, washed with saline and dried from liquid excess. Tissues were then placed in a homemade filtered centrifugation tube supplemented with a 20-µm nylon mesh filter (Repligen) and centrifuged at 400*g* at 4 °C for 10 min. Between 1 and 14 µl of TIF was collected and stored on dry ice. TIF volume was used to determine the metabolite concentration measured by MS.

### MS of TIF

Metabolites were extracted by the addition of 800 μl of MS-grade methanol–water buffer (methanol:water 5:3 (vol/vol)) containing the internal standards glutaric acid (5 μg ml^–1^; Sigma-Aldrich, G3407) and [^13^C_6_]glucose (30 μg ml^–1^; Cambridge Isotope Laboratories, CLM-1396), followed by 500 μl of chloroform. Samples were then vortexed and centrifuged at 4 °C for 10 min each. The polar (top) phases were collected, divided into two equal parts for gas chromatography–MS (GC–MS) and LC–MS analysis and dried using a vacuum concentrator. The dried metabolite extracts were stored at −80 °C until analysis.

Glucose was analyzed by GC–MS, whereas UDP, UTP, cytidine, uridine and glutamine were analyzed by LC–MS. For GC measurements, a standard curve of glucose (Sigma-Aldrich, G7021) was used to calculate the concentration of the metabolite in the samples. The standard curve was extracted in parallel with the samples.

UDP, UTP, cytidine, uridine and glutamine measurements were acquired by LC–MS using a Dionex UltiMate 3000 LC System (Thermo Scientific) with a thermal autosampler set at 4 °C coupled to a Q Exactive Orbitrap mass spectrometer (Thermo Scientific). Standard curves for UDP (Sigma-Aldrich, 94330), UTP (Jena Bioscience, NU-1024S), cytidine (Sigma-Aldrich, C4654), uridine (Sigma-Aldrich, U3003) and glutamine (Gibco, 25030-34) were used to calculate the concentration of these metabolites in the samples. All raw data are available at Metabolomics Workbench^71^ (DATATRACK ID 4718, Study ID ST003154).

### BMDM isolation and polarization

Mouse BMDMs were derived as described previously^26,30^. For polarization assays, 2 × 10^5^ Panc02 cancer cells (sgNT or sgCda) were seeded 48 h before the addition of 4 × 10^5^ BMDMs. At this point, the medium was replaced with DMEM, DMEM + 10 nM IL-4, DMEM + 100 µM UDP or DMEM + 100 µM UDP + 10 µM MRS2578 (Selleck Chemicals, S2855). After 36 h of coculture, polarization was assessed by flow cytometry.

To differentiate BMDMs toward TAMs-L, 7 × 10^6^ BMDMs were seeded in treated Petri dishes (Corning 60-mm TC-treated culture dish) in DMEM supplemented with 10% FBS, 1% Pen/Strep and 20% tumor-conditioned medium for 18 h at 37 °C in a 5% CO_2_ humidified atmosphere.

### BMDM electroporation

Silencing of *P2ry6* or *P2ry14* was achieved by electroporation with specific short interfering RNAs (siRNAs). Briefly, 8 × 10^6^ BMDMs were resuspended in 500 μl of Opti-MEM and electroporated (250 V, 950 mF, ∞Ω) with 100 pmol of each of three siRNAs in combination. Following 24 h of incubation in DMEM supplemented with 10% FBS, 1% Pen/Strep and 2 mM glutamine (Gibco) at 37 °C in a 5% CO_2_ humidified atmosphere, a migration assay was performed.

Commercially available siRNAs were purchased from ID Technology or Invitrogen (scrambled control), and their assay IDs are listed in Supplementary Table 2.

### BMDM migration assay

For migration assays, 1 × 10^5^ mouse BMDMs were seeded on 8-μm polycarbonate membranes (Transwell, Costar) with or without 10 μM MRS2578 (Selleck Chemicals, S2855). When indicated, 2 × 10^5^ sgNT and sgCda cells were seeded in the bottom chambers 36 h before macrophage migration in DMEM supplemented with 2% FBS and 1% Pen/Strep. After incubation, 100 μM UDP or 10 μM MRS2578 (Selleck Chemicals, S2855) was added to the chamber. After 6 h of incubation, the cells were removed from the top of each membrane. The migrated cells were fixed in 4% paraformaldehyde and stained with crystal violet (2.5 g l^−1^). Images were acquired with an Olympus BX41 microscope and CellSense imaging software (v.1.18).

### Fluorimetric intracellular calcium measurements in BMDMs

Mouse BMDMs from *P2ry6*^WT^ and *P2ry6*^ΔMy^ animals were seeded in 96-well plates with a clear film bottom (Greiner, 655090) at 1 × 10^5^ cells per well and cultured overnight at 37 °C in DMEM supplemented with 10% FBS and 1% Pen/Strep. Cells were then incubated with the ratiometric calcium-sensitive dye Fura-2 AM (1 µM; Biotium, 50033) and 0.06% Pluronic F-127 (Invitrogen, P3000MP) for 30 min, after which the medium was aspirated and replaced by assay buffer containing 150 mM NaCl, 6 mM KCl, 2 mM CaCl_2_, 1.5 mM MgCl_2_ and 10 mM HEPES (pH 7.4 with NaOH). Plates were then transferred to a fluorescence plate reader (Molecular Devices, FlexStation 3). Changes in intracellular calcium after stimulation with different concentrations of UDP or UTP were quantified as Δratio_normalized_, which was calculated as the increase in the ratio of the Fura-2 fluorescence signal after excitation at 340 and 380 nm (*F*_340_/*F*_380_) normalized to the response to the ionophore ionomycin (2 µM; Thermo Scientific, I24222). Where indicated, cells were preincubated for 30 min with MRS2578 (10 µM; Selleck Chemicals, S2855) before the Fura-2 assay.

### PBMC isolation, MDM differentiation and polarization

Human buffy coats were obtained from healthy anonymized donors at the Biobank Rode Kruis-Vlaanderen (institutional approval RKOV_19015). PBMCs were isolated by Ficoll density gradient centrifugation (Axis-Shield, 1114545) and washed in PBS containing 1 mM EDTA (dilution 1:4). Monocytes were then isolated using magnetic CD14-conjugated microbeads (Miltenyi Biotec, 130-050-201) according to the manufacturer’s instructions. To obtain MDMs, monocytes were cultured in six-well plates (1 × 10^6^ cells per well) in RPMI supplemented with 10% FBS, 2 mM l-glutamine, 1% Pen/Strep and 25 ng ml^−1^ recombinant human macrophage colony-stimulating factor (PeproTech, 300-25) for 6 days. On the third day, the original medium was combined with 50% fresh medium with macrophage colony-stimulating factor. On day 6, MDMs were polarized toward an M1-like (10 ng ml^−1^ INFγ (PeproTech, 300-02) + 100 ng ml^−1^ LPS (Sigma-Aldrich, L2630)) or M2-like (20 ng ml^−1^ IL-4; PeproTech, 300-04) phenotype for 48 h. Macrophages were then collected, resuspended in fluorescence-activated cell sorting (FACS) buffer and stained (30 min at 4 °C) with fixable viability dye (Thermo Fisher, 65-0865-18), Fc receptor binding inhibitor (eBioscience, 14-9161-71) and antibodies to human CD14, P2RY6, CD80, CD115, HLA-DR, CD163 and CD206. Samples were analyzed using an LSRFortessa (BD Biosciences) flow cytometer. FMO or IgG isotype controls were used to ensure proper gating of positive populations. Data were collected and analyzed with BD FACSDiva (v.9.0) and FlowJo software (v.10.8.1), respectively.

### OT-I T cell preparation and preactivation

Total splenocytes from OT-I mice were isolated from spleens by filtering the cells through a 40-μm-pore cell strainer in sterile PBS and centrifuging at 350*g* for 7 min. Red blood cells were lysed using Hybri-Max (Sigma-Aldrich) buffer. Total splenocytes were cultured in 1 ml of T cell medium (RPMI supplemented with 10% FBS, 1% Pen/Strep, 1% nonessential amino acids, 1% sodium pyruvate and 25 μM β-mercaptoethanol) at 37 °C in a humidified 5% CO_2_ incubator. As detailed below, OT-I T cells were either preactivated for 3 days with 1 μg ml^−1^ soluble anti-mouse CD28 (BD Biosciences) or 1 μg ml^−1^ ‘SIINFEKL’ peptide (IBA Lifesciences) and 10 ng ml^−1^ recombinant human IL-2 (PeproTech). After 72 h, activated OT-I T cells were transferred into fresh medium containing IL-2 and allowed to expand for 5–7 days.

### FACS analysis of OT-I T cell cytotoxicity, activation and proliferation

sgNT and sgCda OVA-expressing Panc02 cells were labeled with 1 μM carboxyfluorescein succinimidyl ester (CFSE; Thermo Fisher Scientific) for 10 min at room temperature. sgNT and sgCda non-OVA Panc02 cells were labeled with 3.5 μM Violet cell tracer (Thermo Fisher Scientific) at 37 °C for 20 min. Mixed populations of OVA-expressing CFSE-labeled sgNT or sgCda Panc02 cells and non-OVA-expressing Violet-labeled sgNT or sgCda Panc02 cells were seeded at a 1:1 ratio and cocultured with preactivated OT-I CD8^+^ T cells for 24 h at the indicated effector:target ratios. Cells were stained with Zombie NIRTM Fixable Viability Dye, washed and analyzed by flow cytometry for changes in the ratio of CFSE^+^:Violet^+^ cells.

OVA-expressing sgNT or sgCda cells were cocultured with or without BMDMs at a 1:4 ratio for 24 h, after which, total splenocytes from OT-I mice were added at a 1:15 (cancer cell:splenocyte) ratio for 36 h in T cell medium with 10 ng ml^−1^ recombinant human IL-2. Cells were then stained with Fixable viability dye (eBioscience, 65-0866-14) and the following cocktail of antibodies for 30 min at 4 °C: anti-mouse TCR-β chain, anti-CD4, anti-CD8, anti-IFNγ, anti-GZMB and anti-Ki-67. Data were collected and analyzed with BD FACSDiva (v.9.0) and FlowJo software (v.10.8.1), respectively.

### FACS analysis of SIINFEKL-bound MHC class I and CD274 expression

OVA-expressing sgNT and sgCda Panc02 cells were seeded at a density of 500,000 cells per well in 12-well plates with or without 1,000 U ml^−1^ IFNγ (Peprotech). On day 3, 50,000 cells were seeded in 96-well round-bottom plates. After 24 h, the cells were stained for 30 min at 4 °C with the viability dye (eBioscience, 65-0863-18), anti-H-2K^b^ MHC class I and 25-D1.16 and were analyzed by flow cytometry.

To assess CD274 expression, 0.1 × 10^6^ Panc02, KPC FC1245 or FC1199 cells were seeded in complete DMEM for 6 h. Medium was then replaced with complete DMEM supplemented with 100 ng ml^−1^ IFNγ (Thermo Fisher Scientific, BMS326). After 24 h, cancer cells were collected, and CD274 expression was assessed by flow cytometry. Samples were analyzed using an LSRFortessa (BD Biosciences) flow cytometer. An FMO control was used to ensure proper gating of positive populations. Data were collected and analyzed with BD FACSDiva (v.9.0) and FlowJo software (v.10.8.1), respectively.

### Histology and immunostaining

For mouse tumor tissue staining, deparaffinization and antigen retrieval (Dako) were performed, followed by blocking with preimmune donkey serum (PID; Sigma-Aldrich) diluted 1:10 in Tris-NaCl blocking buffer (TNB). Tissue sections were then incubated with primary antibodies (rabbit anti-mouse CD8, rat anti-mouse F4/80 or goat anti-mouse MMR/CD206) + 10% PID in TNB overnight at room temperature. Sections were then incubated with the appropriate biotin-conjugated secondary antibody in TNB for 45 min. F4/80, CD8 and MMR/CD206 immune complexes were then amplified with streptavidin-HRP conjugate and cyanine 3 (PerkinElmer) or a TSA Fluorescein kit (PerkinElmer) according to the manufacturer’s instructions. Hoechst solution (Life Technologies; 1:1,000) was used to visualize nuclei, and slides were mounted with ProLong Gold mounting medium without DAPI (Invitrogen). Imaging and microscopic analyses were performed with an Olympus BX41 microscope and CellSense imaging software (v.1.18).

For human tumor sections from cohort 1 (Supplementary Table 1), immunohistochemical stains were performed on a Bond-III Fully Automated IHC and ISH Stainer (Leica Biosystems). Primary antibodies to CDA and CD8 were used in combination with the EnVision + Dual Link System-HRP (Dako). Bond Polymer Refine Red Detection and Bond Polymer Refine Detection kits (Leica Biosystems) were used following the manufacturer’s instructions. For CD68 and CD206 stainings, the secondary antibodies were Alexa Fluor 488 anti-mouse IgG3 and Alexa Fluor 647 anti-mouse IgG2B, respectively. Slides were scanned with a Zeiss Axio Scan. Digital images were analyzed and processed by an expert pathologist. Autofluorescence was subtracted using a reference image of the same tissue. CD8^+^, CD68^+^ and CD68^+^CD206^+^ cells were counted in ten random high-power fields, five high-power fields in the tumor border and five in the tumor center. High expression of CDA was defined as >10% diffuse strong expression in tumor cells (where diffuse refers to areas of cells within the cross-section, excluding some possible nonspecific staining in the borders or next to necrosis).

For immunofluorescence co-stainings of nine PDAC tumor sections (out of cohort 1) for CD31, CD68, CK7 and CDA, deparaffinization and antigen retrieval (Dako) were performed, followed by blocking with PID (Sigma-Aldrich) diluted 1:10 in TNB. Afterward, the sections were incubated with primary antibodies (rabbit anti-human CDA, mouse anti-human CD68, mouse anti-human CK7 or mouse anti-human CD31) overnight at room temperature. Sections were then incubated with the proper biotin-conjugated secondary antibody. Immune complexes were amplified with streptavidin-HRP conjugate (PerkinElmer) and a TSA Fluorescein kit (PerkinElmer; for CDA) or cyanine 3 (PerkinElmer; for CD31, CD68 and CK7), according to the manufacturer’s instructions. Hoechst solution (Life Technologies; 1:1,000) was used to visualize nuclei, and slides were mounted with ProLong Gold mounting medium without DAPI (Invitrogen). Imaging and microscopic analysis were performed with an Olympus BX41 microscope and CellSense imaging software (v.1.18).

### Transcriptomics (unique mutations)

Total RNA was extracted using TRIzol (Life Technologies), and polyadenylated fragments were isolated, reverse transcribed and converted into indexed sequencing libraries using a KAPA stranded mRNA-seq kit (Sopachem). The first 50 bases of these libraries were sequenced on a HiSeq 2500 system (Illumina). After removal of the sequencing adapters, raw reads were mapped to the reference transcriptome and genome (GRCm38/mm10) using the Bowtie TopHat pipeline^72^. Mapped reads were assigned to Ensembl gene IDs by HTSeq, resulting in, on average, 35,159,030 ± 6,605,340 assigned counts per sample. Variants were identified following GATK’s best practice, and only variants unique in one sample were retained, resulting in, on average, 1,132 ± 266 mutations.

### Meta-analysis

The meta-analysis approach used to identify antiangiogenic target genes was similar to that reported previously^73^. For human transcriptomics datasets, we used RECIST criteria, as provided by the authors, to classify tumors according to treatment response. For in-house mouse data, we used tumor growth curves to classify tumors into responsive, low-responsive and nonresponsive groups.

We performed differential analysis between responsive and nonresponsive tumors on each dataset separately to identify differentially expressed genes and their false discovery rate-corrected *P* values (limma package^74^). We then integrated differential expression results using a product-based meta-analysis^75^. Briefly, we ranked the results of each pairwise comparison by log_2_ (fold change). The most upregulated genes received the lowest rank number (top-ranking genes), and the most downregulated genes received the highest rank number. We combined the rank numbers for all genes in all pairwise comparisons by calculating their product to obtain a final list of ranked genes associated with immunotherapy resistance. To assess statistical significance, we used a recently developed algorithm to determine accurate approximate *P* values for each gene based on the rank product statistic^76^ and obtained Benjamini–Hochberg adjusted *P* values using the R package qvalue^77^. We filtered rank-based meta-analysis results for metabolic genes, as described previously^78^.

### Human RNA-seq

Various scRNA-seq pan-tumor maps were obtained from Tumor Immune Single Cell Hub^43^. We derived the average expression of *CDA* (in cancer cells only per dataset), *IFNG* or *PRF1* (in CD8^+^ T cells only per dataset) or *P2RY6*, *CD163*, *MSR1* or *MRC1* (in macrophages only per dataset) and performed a Pearson’s correlation between them using the 16 datasets as variables.

Individuals with PDAC in TCGA were subdivided into two groups, that is, macrophage^high^CD8^+^ T cell^low^ or macrophage^low^CD8^+^ T cell^high^ (where macrophages or CD8^+^ T cell bifurcations were based on predefined genetic signatures) using established computational workflows, and *CDA* expression in these two subgroups was derived^79,80^. Expression profiles for PDAC samples in log_2_p(TPM + 0.001) were further processed using Spearman’s gene-to-gene correlation with the Python Scipy SpearmanR module^81^.

DESeq2 prenormalized data by the original authors were downloaded from GSE179351. Expression of *PDCD1* and *CDA* in individuals with PDAC before treatment with ICB plus radiotherapy was analyzed and represented as dot plots. Dot sizes represent the proportion of individuals with nonzero expression. The color scale represents standard-scaled mean expression per genetic marker. ‘NoResponse’ includes individuals that exhibited either stable or progressive disease, whereas ‘Response’ includes individuals that achieved either partial or complete response, as defined in Supplementary Table 8 of Parikh et al. ^22^ (NCT03104439).

### FACS analysis on tumors

Mouse tumors were collected and minced in αMEM (Lonza) supplemented with 5% FBS, 1% Pen/Strep, 50 μM β-mercaptoethanol (Gibco), 5 U ml^−1^ DNase I (QIAGEN), 0.85 mg ml^−1^ collagenase V (collagenase from *Clostridium histolyticum*; Sigma-Aldrich), 1.25 mg ml^−1^ collagenase D (collagenase from *C. histolyticum*; Roche) and 1 mg ml^−1^ Dispase II (Gibco) and incubated for 30 min at 37 °C. The digested tissue was filtered using a 70-μm-pore strainer, and cells were centrifuged for 5 min at 300*g*. The samples were resuspended in Red Blood Cell Lysing Buffer Hybri-Max (Sigma-Aldrich) for 30 s, inactivated with FACS buffer (PBS containing 2% FBS and 2 mM EDTA) and centrifuged. The cell pellets were resuspended in FACS buffer and filtered with a 40-μm-pore strainer. Cells were resuspended in FACS buffer and, for intracellular measurement of IFNγ and GZMB, single-cell suspensions were cultured in RPMI supplemented with 10% FBS, 1% glutamine and 1% Pen/Strep and stimulated with phorbol 12-myristate 13-acetate/ionomycin Cell Stimulation Cocktail (eBioscience, 1:500) in the presence of brefeldin A (BioLegend; 1:1,000) or monensin (eBioscience; 1:1,000) for 4 h at 37 °C. Subsequently, cells were incubated for 15 min at 4 °C with mouse BD Fc block-purified monoclonal rat anti-mouse CD16/CD32 (BD Pharmingen, 553142) and stained with Fixable viability dye (eBioscience, 65-0866-14) and the following antibodies for 30 min at 4 °C: anti-mouse CD45, CD11b, TCRβ chain, CD4, CD8, CD69, F4/80, IFNγ, GZMB, MHC class II, CD11c, CD206, Ly6G, CD335 (NKp46), Foxp3, CD25 and P2RY_6_. Cells were washed and analyzed by FACS using an LRSFortessa X-20 (BD Bioscience).

Fresh human PDAC samples were digested with Liberase DL (Sigma-Aldrich, 5401160001), Liberase TL (Sigma-Aldrich, 5401020001) and DNase I (Sigma-Aldrich, D4527) in αMEM supplemented with 2% FBS. The digestion was performed using a MACS dissociator, following the manufacturer’s instructions (Miltenyi Biotec). Tumor samples were then resuspended in FACS buffer and filtered through 70- and 40-μm-pore strainers. Subsequently, samples were incubated for 15 min at 4 °C with human Fc receptor binding inhibitor (eBioscience, 14-9161-71) and stained for 30 min at 4 °C with the following anti-human antibodies: CD14, P2RY_6_, CD204, CD11b, CD115, HLA-DR, CD3, CD163, CD206, CD45, CD15, CD31 and CD326. Cells were then washed and analyzed by FACS using an LRSFortessa X-20 (BD Bioscience). FMO controls, unstained control and single-staining or IgG isotype controls were performed to ensure proper gating strategy. Data were collected and analyzed with a BD FACSDiva (v.9.0) and FlowJo software (v.10.8.1), respectively.

### Cell sorting

Panc02 CD90.1 and KPC FC1245-CD90.1 tumors were processed as previously mentioned. After obtaining single-cell suspensions, CD45 enrichment was performed by following the manufacturer’s instructions (CD45 MicroBeads, mouse, 130-052-301).

Cells (CD45^+^ and CD45^–^) were then incubated for 15 min at 4 °C with mouse BD Fc block-purified monoclonal rat anti-mouse CD16/CD32 (BD Pharmingen, 553142) and stained with Fixable viability dye (eBioscience, 65-0866-14 or 65-0863-18) and the following cocktail of antibodies for 30 min at 4 °C: anti-mouse CD45, CD11b, F4/80, TCRβ chain, CD90.1, CD90.2, CD31, CD11c and Ly6G. Cells were washed and sorted using a FACSAria Fusion (BD Biosciences) flow cytometer. Data were collected and analyzed with BD FACSDiva (v.9.0) and FlowJo software (v.10.8.1), respectively. FMO controls, unstained controls and single-staining controls were performed to ensure proper gating. Postsort purity of the gating strategy is included in Supplementary Figs. 1–4.

### Statistics and reproducibility

All statistical analyses were performed using GraphPad Prism 9.5.0 software. Statistical significance was calculated by two-tailed unpaired *t*-test on two experimental conditions or multiple two-tailed unpaired *t*-tests and two-way ANOVA when repeated measures were compared, with *P* < 0.05 considered statistically significant as indicated in each figure legend. The exact *P* values are reported in each figure, except when *P* < 0.0001.

No statistical methods were used to predetermine sample sizes, but our sample sizes are similar to those reported in previous publications for the same type of experiments and readout^70,82,83^. The exact sample sizes are indicated in the figure legends. Independent experiments were pooled and analyzed together whenever possible, as detailed in the figure legends. Where appropriate, Shapiro–Wilk tests were performed to check the distribution of samples. Detection of mathematical outliers was then performed using the Grubbs’ test in GraphPad. Animals were excluded only if they died, had to be killed according to protocols approved by the animal experimental committees or when the measurement was not reliable for technical issues (specifically for ultrasound). For in vitro experiments, no data were excluded. For in vivo studies, tumor measurement, treatment and analysis were performed blindly by different researchers to ensure that the studies were run in a blinded manner. Animals were randomized, with each group receiving mice with similar tumor size or similar body weight. For in vitro studies, randomization and blinding of cell lines was not possible; however, all cell lines were treated identically without prior designation. All graphs show mean values ± s.e.m.

### Reporting summary

Further information on research design is available in the Nature Portfolio Reporting Summary linked to this article.

### Supplementary information

Supplementary Information Supplementary Figs. 1–6.

Reporting Summary

Supplementary Table 1 Clinical information of individuals (from cohort 1) and list of oligonucleotide sequences.

### Source data

Source Data Fig. 1 Statistical source data.

Source Data Fig. 2 Statistical source data.

Source Data Fig. 3 Statistical source data.

Source Data Fig. 4 Statistical source data.

Source Data Fig. 5 Statistical source data.

Source Data Fig. 6 Statistical source data.

Source Data Fig. 7 Statistical source data.

Source Data Fig. 8 Statistical source data.

Source Data Extended Data Fig. 1 Statistical source data.

Source Data Extended Data Fig. 2 Statistical source data.

Source Data Extended Data Fig. 3 Statistical source data.

Source Data Extended Data Fig. 4 Statistical source data.

Source Data Extended Data Fig. 5 Statistical source data.

Source Data Extended Data Fig. 6 Statistical source data.

Source Data Extended Data Fig. 7 Statistical source data.

Source Data Extended Data Figs. 2 and 3 Unprocessed western blots.

## Data Availability

In-house mouse bulk RNA-seq datasets that support the findings of this study have been deposited in the Gene Expression Omnibus under accession number GSE196790. Publicly available mouse bulk RNA-seq datasets can be found in refs. ^30,38^ under accession numbers GSE126722 and E-MTAB-5032. A publicly available mouse orthotopic KPC scRNA-seq dataset from ref. ^11^ under accession number GSE129455 was used. For the meta-analysis, publicly available human metastatic melanoma and renal cancer datasets can be found in refs. ^3,5,69^ under accession numbers GSE78220 and GSE67501 and in dbGap under accession number phs000452.v2.p1. The bulk RNA-seq human PDAC data were derived from ref. ^22^ under accession number GSE179351 and from the TCGA Research Network. TGCA data were downloaded from the UCSC Xena platform (http://xena.ucsc.edu/). scRNA-seq data of human PDAC samples can be found in ref. ^21^ under accession number GSA CRA001160. Various human (stomach adenocarcinoma, skin cutaneous melanoma, pancreatic adenocarcinoma, ovarian cancer, non-small cell lung cancer, liver hepatocellular carcinoma, head and neck squamous cell carcinoma, glioblastoma/glioma, colorectal cancer, cholangiocarcinoma and basal cell carcinoma) scRNA-seq datasets were derived from Tumor Immune Single Cell Hub under accession numbers GSE134520, GSE72056, GSE111672, CRA001160, GSE118828, GSE143423, GSE127465, GSE117570, E-MTAB-6149, GSE125449, GSE103322, GSE141982, GSE138794, GSE146771, GSE125449 and GSE123813. In-house LC–MS (in vitro and in vivo) data have been deposited in Metabolomics Workbench^71^ under Study IDs ST003154 and ST002791. Source data are provided with this paper. All other data supporting the findings of this study are available from the corresponding author upon reasonable request.

## Code availability

All code used to analyze data in this study is available on GitHub at https://github.com/mazzonelab. All other code supporting the findings of this study are available in the Methods or are available from the corresponding author upon reasonable request.
