# Supplementary material for: Nucleotide metabolism in cancer cells fuels a UDP-driven macrophage cross-talk, promoting immunosuppression and immunotherapy resistance
Source: Nat Cancer. 2024 Jun 6;5(8):1206–26. doi: 10.1038/s43018-024-00771-8 (PMC11358017; doi:10.1038/s43018-024-00771-8)
Supplement: Supplementary file 1 — Supplementary Figs. 1–6. [file 43018_2024_771_MOESM1_ESM.pdf]

nature cancer

Supplementary information https://doi.org/10.1038/s43018-024-00771-8

Nucleotide metabolism in cancer cells fuels a UDP-driven macrophage cross-talk, promoting immunosuppression and immunotherapy resistance

In the format provided by the authors and unedited

Supplementary Figure 1

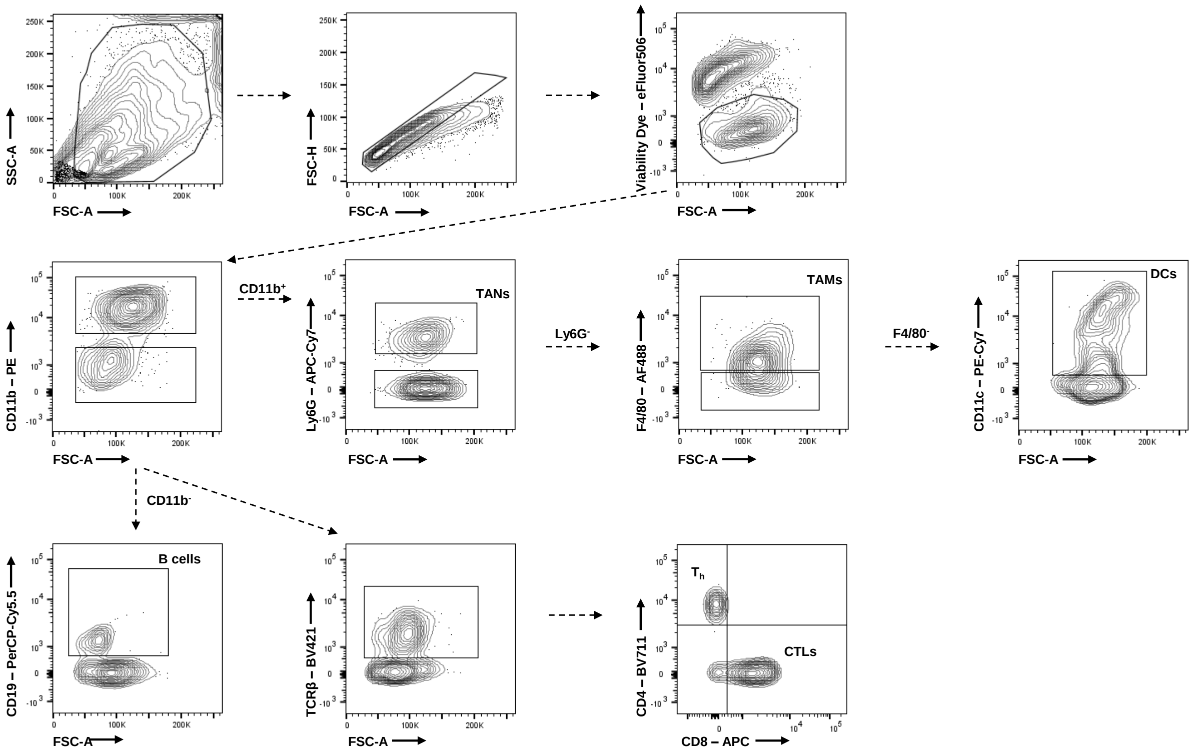

Representative gating strategy for determination of different immune cell populations. Cells were gated starting from: FSC-SSC (cells), FSC-A-FSC-H (singlets), Viability dye-FSC-A (viable cells) to exclude debris and dead cells. Within the viable cell gate, TANs (CD45$^+$, CD11b$^+$, Ly6G$^+$), TAMs (CD45$^+$, CD11b$^+$, F4/80$^+$), DCs (CD45$^+$, CD11b$^+$, F4/80$^-$, CD11c$^+$), B cells (CD45$^+$, CD11b$^-$, CD19$^+$), T_h (CD45$^+$, CD11b$^-$, TCRβ$^+$,CD4$^+$) and CTLs (CD45$^+$, CD11b$^-$, TCRβ$^+$,CD8$^+$) are identified. FMO controls were used to define the gates properly.TANs = Tumor-associated neutrophils; TAMs = Tumor-associated macrophages; DCs = Dendritic cells; T_h = T helper; CTLs = cytotoxic T lymphocytes.

Supplementary Figure 2

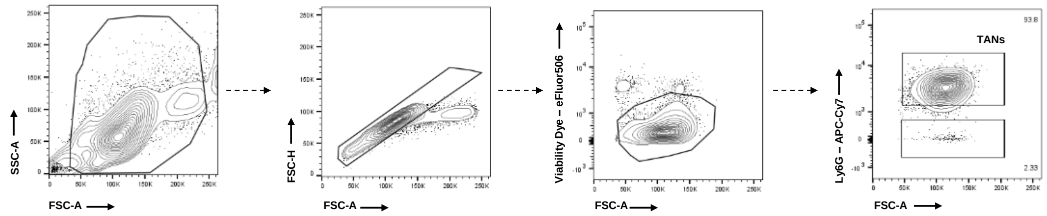

Post sort purity check. Tumor-associated neutrophils (TANs).

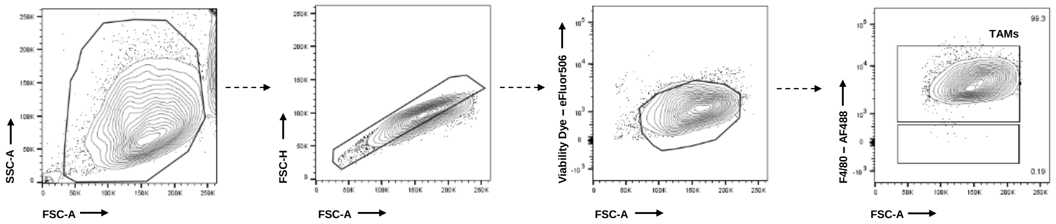

Post sort purity check. Tumor-associated macrophages (TAMs).

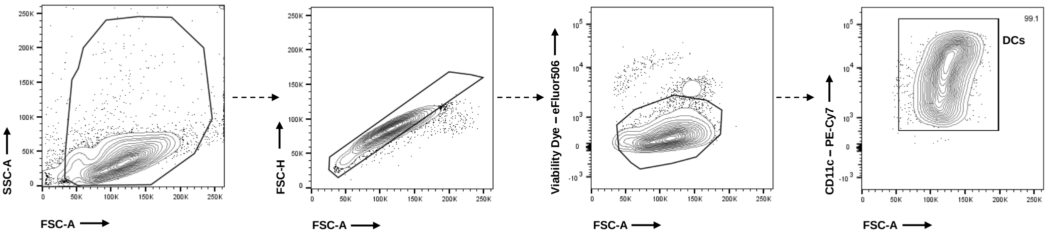

Post sort purity check. Dendritic cells (DCs).

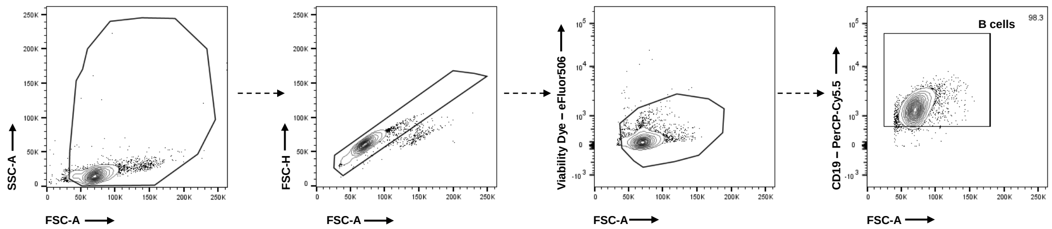

Post sort purity check. B cells.

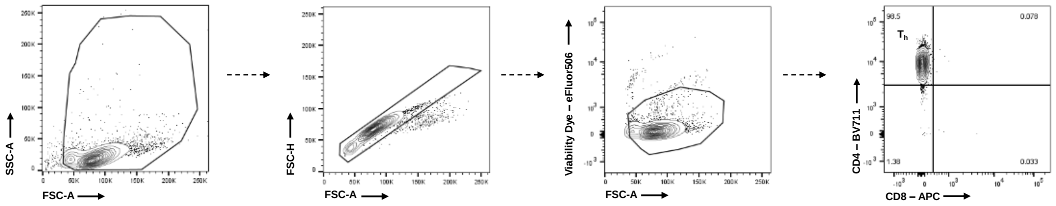

Post sort purity check. T helper (T_h).

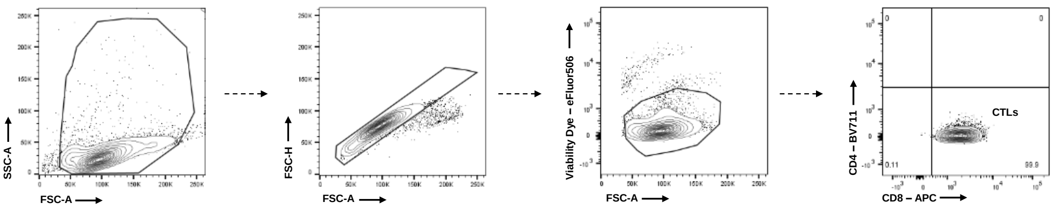

Post sort purity check. Cytotoxic T lymphocytes (CTLs).

Supplementary Figure 3

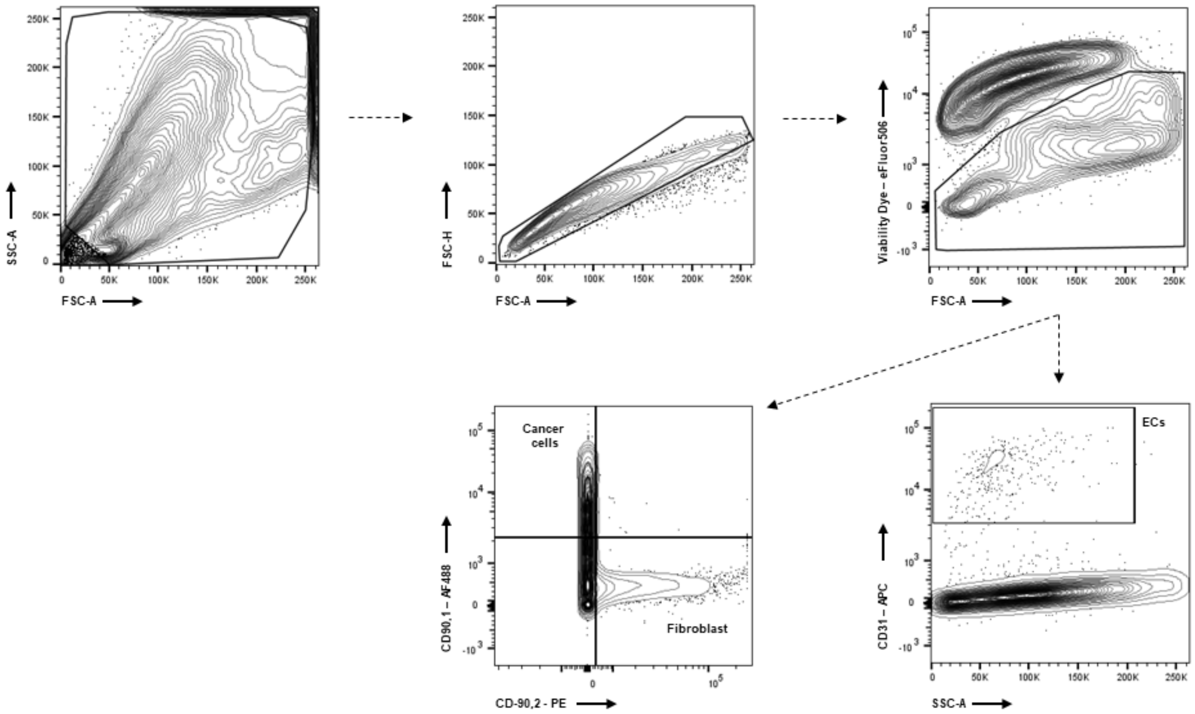

Representative gating strategy for determination of different cell populations. Cells were gated starting from: FSC-SSC (cells), FSC-A-FSC-H (singlets), Viability dye-FSC-A (viable cells) to exclude debris and dead cells. Within the viable cell gate, Cancer cells ($CD90.1^+$, $CD90.2^-$), Fibroblast ($CD90.1^-$, $CD90.2^+$) and ECs ($CD31^+$) are identified. FMO controls were used to define the gates properly. ECs = Endothelial cells.

Supplementary Figure 4

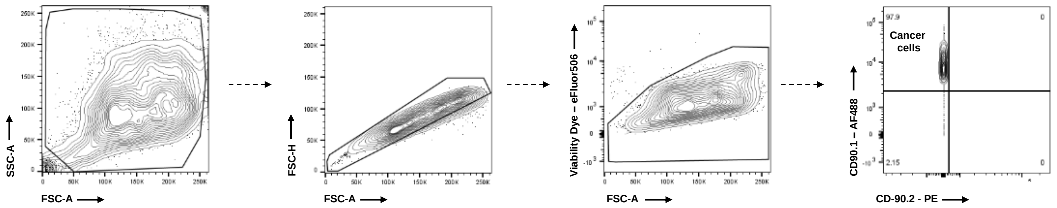

Post sort purity check. Cancer cells.

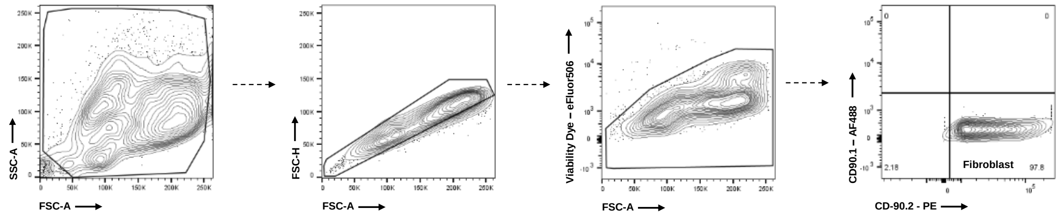

Post sort purity check. Fibroblast.

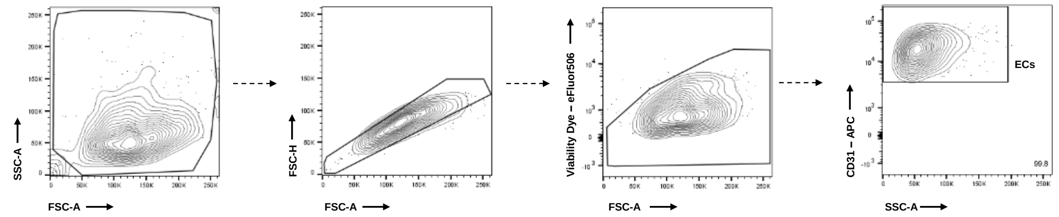

Post sort purity check. Endothelial cells (ECs).

Supplementary Figure 5

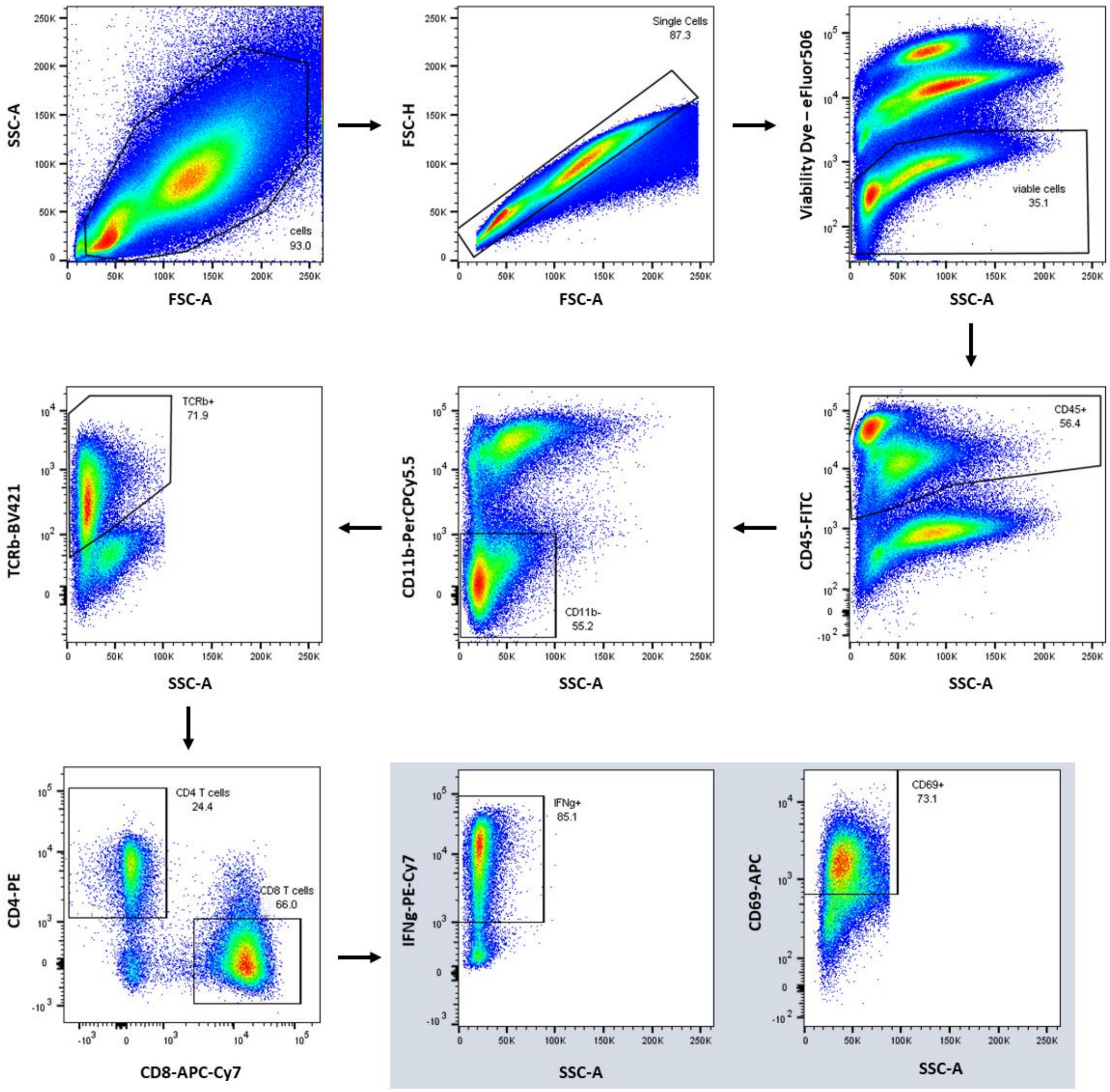

Gating strategy for determination of CD4$^+$ and CD8$^+$ T cells. CD4$^+$ and CD8$^+$ T cells were gated starting from: FSC-SSC (cells), FSC-A-FSC-H (singlets), Viability dye-SSC-A (viable cells) to exclude debris and dead cells. Within the viable cell gate, CD4$^+$ T cells (CD45$^+$, CD11b$^-$, TCRb$^+$, CD4$^+$), CD8$^+$ T cells (CD45$^+$, CD11b$^-$, TCRb$^+$, CD8$^+$) are identified. IFN-γ$^+$CD8$^+$ T cells and CD69$^+$CD8$^+$ T cells were properly gated by using FMO controls.

Supplementary Figure 6

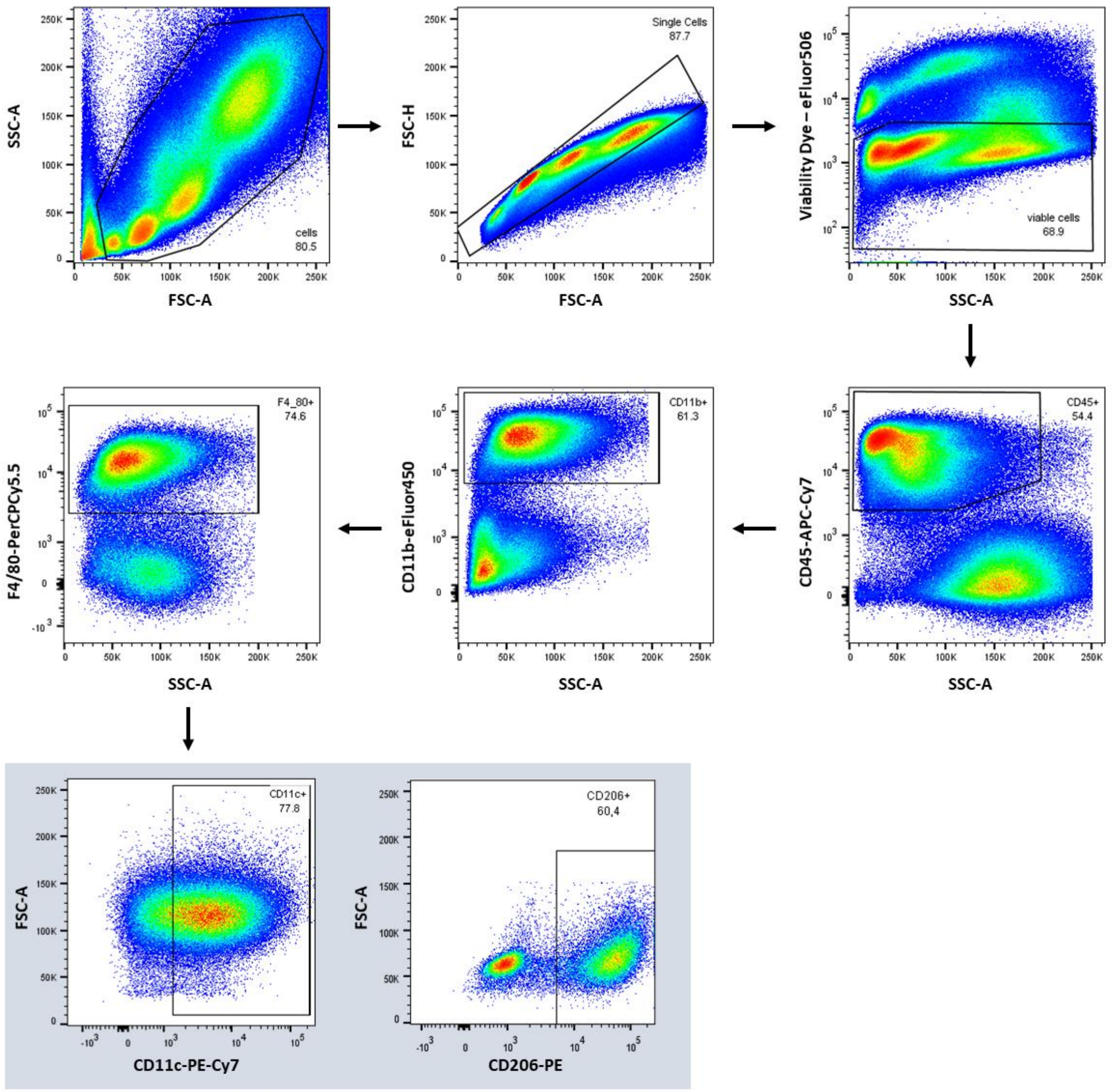

Gating strategy for determination of TAMs. F4/80$^+$ cells were gated starting from: FSC-SSC (cells), FSC-A-FSC-H (singlets), Viability dye-SSC-A (viable cells) to exclude debris and dead cells. Within the viable cell gate, TAMs (CD45$^+$, CD11b$^+$, F4/80$^+$) are identified. CD11c$^+$F4/80$^+$ cells (M1-like, anti-tumoral TAMs) and CD206$^+$F4/80$^+$ cells (M2-like, immunosuppressive TAMs) were properly gated by using FMO controls.
