# Supplementary material for: Nucleotide metabolism in cancer cells fuels a UDP-driven macrophage cross-talk, promoting immunosuppression and immunotherapy resistance
Source: Nat Cancer. 2024 Jun 6;5(8):1206–26. doi: 10.1038/s43018-024-00771-8 (PMC11358017; doi:10.1038/s43018-024-00771-8)
Supplement: Supplementary file 2 — Reporting Summary [file 43018_2024_771_MOESM2_ESM.pdf]

nature portfolio

Corresponding author(s): Massimiliano Mazzone

Last updated by author(s): Apr 20, 2024

Reporting Summary

Nature Portfolio wishes to improve the reproducibility of the work that we publish. This form provides structure for consistency and transparency in reporting. For further information on Nature Portfolio policies, see our Editorial Policies and the Editorial Policy Checklist.

Statistics

For all statistical analyses, confirm that the following items are present in the figure legend, table legend, main text, or Methods section.

n/a	Confirmed	
☐	☒	The exact sample size (*n*) for each experimental group/condition, given as a discrete number and unit of measurement
☐	☒	A statement on whether measurements were taken from distinct samples or whether the same sample was measured repeatedly
☐	☒	The statistical test(s) used AND whether they are one- or two-sided<br>*Only common tests should be described solely by name; describe more complex techniques in the Methods section.*
☐	☒	A description of all covariates tested
☐	☒	A description of any assumptions or corrections, such as tests of normality and adjustment for multiple comparisons
☐	☒	A full description of the statistical parameters including central tendency (e.g. means) or other basic estimates (e.g. regression coefficient) AND variation (e.g. standard deviation) or associated estimates of uncertainty (e.g. confidence intervals)
☐	☒	For null hypothesis testing, the test statistic (e.g. *F*, *t*, *r*) with confidence intervals, effect sizes, degrees of freedom and *P* value noted<br>*Give P values as exact values whenever suitable.*
☒	☐	For Bayesian analysis, information on the choice of priors and Markov chain Monte Carlo settings
☒	☐	For hierarchical and complex designs, identification of the appropriate level for tests and full reporting of outcomes
☐	☒	Estimates of effect sizes (e.g. Cohen's *d*, Pearson's *r*), indicating how they were calculated

Our web collection on statistics for biologists contains articles on many of the points above.

Software and code

Policy information about availability of computer code

Data collection	Microscopy images were acquired by using Olympus BX41 microscope and CellSense imaging software (v1.18) or Zeiss Axio Scan as indicated in Material and Methods. QuantStudio 12K Flex Real-Time PCR System (Applied Biosystems; v1.4) was used for real-time PCR. Western blot imaging acquisition was performed by using ImageQuant LAS 4000, GE Health Care Life Science Technologies. Flow cytometry data collection was done with BD FACS DIVA software (v9.0). Tumor area by Ultrasound imaging was collected by using Vevo3100 (Vevo LAB 5.7.1) from VisualSonics, Inc. LC-MS analysis was performed by using Q Exactive Hybrid Quadrupole-Orbitrap Mass Spectrometer (Thermo Scientific) or Dionex UltiMate 3000 LC System (Thermo Scientific) as stated in Material and Methods. LC-MS data collection was done using Xcalibur software (Thermo Scientific). Ca2+ measurement was performed by using Molecular Devices, FlexStation 3.
Data analysis	Microscopy images were analyzed by using CellSense imaging software (v1.18). Flow cytometry analysis was performed with the FlowJo software (v10.8.1). Ultrasound images were analyzed using VisualSonics, Inc. imaging software package. LC-MS data analysis was done using Xcalibur software (Thermo Scientific). Bulk-RNAseq reads were mapped to the transcriptome and the mouse reference genome (GRCm38/mm10) using TopHat 2.0 and Bowtie2.0 (Langmead and Salzberg, 2012). Mapped reads were assigned to ensemble gene IDs with the HTSeq software package. Analyses on publicly available murine and human datasets were done using codes mentioned in 'Code availability' section of the manuscript. Statistical analyses on Figure 1b, 1j, 8d and Extended Data Figure 1f were performed in R studio (see 'Code availability'). All other statistical analyses were performed using GraphPad Prism software (v9.5.0).

For manuscripts utilizing custom algorithms or software that are central to the research but not yet described in published literature, software must be made available to editors and reviewers. We strongly encourage code deposition in a community repository (e.g. GitHub). See the Nature Portfolio guidelines for submitting code & software for further information.

Data

Policy information about availability of data

All manuscripts must include a data availability statement. This statement should provide the following information, where applicable:

- Accession codes, unique identifiers, or web links for publicly available datasets
- A description of any restrictions on data availability
- For clinical datasets or third party data, please ensure that the statement adheres to our policy

In-house murine bulk RNA-seq datasets that support the findings of this study have been deposited in the Gene Expression Omnibus (GEO) under accession no. GSE196790. Publicly available murine bulk RNA-seq datasets can be found in ref.31,39 with accession no. GSE126722 and E-MTAB-5032. Publicly available murine orthotopic KPC scRNA-seq dataset from ref.11 with accession no. GSE129455 was used. For the meta-analysis, human metastatic melanoma and renal cancer publicly available datasets can be found in ref.3,5,20 with accession no. GSE78220, GSE67501 and dbGap, phs000452.v2.p1. The bulk RNA-seq human PDAC data were derived from ref.23 with accession no. GSE179351, and from the TCGA Research Network. TGCA data were downloaded from the UCSC Xena platform (http://xena.ucsc.edu/). Single-cell RNA-seq data of human PDAC samples can be found in ref.22 with accession no. GSA CRA001160. Various human (STAD, SKCM, PAAD, OV, NSCLC, LIHC, HNSC, GBM/Glioma, CRC, CHOL, BCC) scRNA-seq datasets were derived from Tumor Immune Single Cell Hub with accession no. GSE134520, GSE72056, GSE111672, CRA001160, GSE118828, GSE143423, GSE127465, GSE117570, EMTAB6149, GSE125449, GSE103322, GSE141982, GSE138794, GSE146771, GSE125449, GSE123813. In-house LC-MS (in vitro and in vivo) data have been deposited in Metabolomics Workbench (ref.71), DATATRACK_ID:4162 and 4718.

Human research participants

Policy information about studies involving human research participants and Sex and Gender in Research.

Reporting on sex and gender	As outlined in the method, tumor samples were collected from 63 treatment-naive PDAC patients (31 males and 32 females, referred as cohort #1; UZ KU Leuven Hospital). No gender related issues are applied to this analysis. All clinical data are extensively described in Supplementary Table 1.<br><br>Sex/gender of participants were determined on self-report; no transgender participants were present in this study population. Both genders were included and we ruled out the possibility that gender could somehow affect our findings.
Population characteristics	Tumor samples were obtained from naive PDAC patients (referred as cohort #1). All clinical data are extensively described in Supplementary Table 1.
Recruitment	Tumor material was collected either from resected primary tumors during surgery or biopsy. The presence of adenocarcinoma was proven on histopathology.
Ethics oversight	The study was approved by the Ethical Committe of the University Hospitals KU Leuven (Leuven, Belgium) with reference number ML3452 (see Supplementary Table 1). All patients were given informed consent.

Note that full information on the approval of the study protocol must also be provided in the manuscript.

Field-specific reporting

Please select the one below that is the best fit for your research. If you are not sure, read the appropriate sections before making your selection.

☒ Life sciences ☐ Behavioural & social sciences ☐ Ecological, evolutionary & environmental sciences

For a reference copy of the document with all sections, see nature.com/documents/nr-reporting-summary-flat.pdf

Life sciences study design

All studies must disclose on these points even when the disclosure is negative.

Sample size	No statistical methods were used to pre-determine sample sizes but our sample sizes are similar to those reported in previous publications for the same type of experiments and readout (Cappellesso et al. Nature Cancer 2022; Celus et al. Cancer Immunol. Res. 2022; Virga et al. Sci. Adv. 2021). The exact sample sizes are indicated in the figure legends.
Data exclusions	Detection of mathematical outliers was performed using the Grubbs' test in GraphPad after checking (with Shapiro-Wilk test) that our data were normally distributed. Animals were excluded only if they died, had to be killed according to protocols approved by the animal experimental committees, or when the measurement was not reliable for technical issues (specifically for ultrasound). For in vitro experiments no data were excluded.
Replication	Independent experiments were performed in a blinded fashion to assure the reproducibility of the experimental findings. In details, for in vitro experiments three biological replicates were performed with similar results; for in vivo studies at least 4 animals were allocated per group.

Randomization

For in vivo experiments, all animals involved were randomized into each experimental groups with similar tumor size or similar body weight. For in vitro studies, randomization of cell lines was not possible; however, all cell lines were treated identically without prior designation.

Blinding

Investigators were blinded to group allocation during data collection and analyses. Indeed, the tumor measurements, treatment and analyses were performed blindly by different researchers to ensure that the studies were run in blinded manner. For in vitro studies, blinding of cell lines was not possible; however, all cell lines were treated identically without prior designation.

Reporting for specific materials, systems and methods

We require information from authors about some types of materials, experimental systems and methods used in many studies. Here, indicate whether each material, system or method listed is relevant to your study. If you are not sure if a list item applies to your research, read the appropriate section before selecting a response.

Materials & experimental systems

n/a	Involved in the study	
☐	☒	Antibodies
☐	☒	Eukaryotic cell lines
☒	☐	Palaeontology and archaeology
☐	☒	Animals and other organisms
☐	☒	Clinical data
☒	☐	Dual use research of concern

Methods

n/a	Involved in the study	
☒	☐	ChIP-seq
☐	☒	Flow cytometry
☒	☐	MRI-based neuroimaging

Antibodies

Antibodies used

For Western Blot. Rabbit Anti-mouse CDA (Sigma-Aldrich, SAB1300716, 1:100 and SAB1300717, 1:500, both polyclonal), HRP-conjugated anti-beta-tubulin (Abcam, ab21058, 1:3000, polyclonal) and HRP-conjugated secondary antibody goat anti-rabbit (Cell Signalling, 7074S, 1:2000 and Santa Cruz biotechnology, sc-2004, 1:2000).

For histology. Rabbit anti-mouse CD8 (Cell Signaling, 98941S, clone D4W2Z, 1:200), rat anti-mouse F4/80 (Serotec, MCA497F, clone CI:A3-1 ,1:100, RRID: AB_872005), goat anti-mouse MMR/CD206 (R&D system, AF2535, 1:100, polyclonal), rabbit anti-human/mouse CDA (Abcam, ab231981, 1:500 IHC or 1:150 IF, polyclonal), mouse anti-human CD8 (Agilent, M7103, clone C8/144B, 1:200), mouse IgG3 anti-human CD68 (Thermofisher, MA5-12407, clone PG-M1, 1:1000, RRID: AB_10979558, or Agilent Dako, M0876, clone PG-M1, 1:100), mouse anti-human CD206 (R&D Systems: MAB25341, clone 685645, 1:1000), mouse anti-human CD31 (Agilent Technologies, JC70A, clone JC/70A, 1:100), mouse anti-human CK7 (Agilent Technologies, M701801, clone OV-TL 12/30, 1:200), biotin-SP-donkey anti-rabbit antibody (Bio-connect, 711-065-152, 1:200, RRID: AB_2340593), biotin-SP-donkey anti-rat (Bio connect, 712-065-153, 1:500, RRID: AB_2315779) and biotin-SP-donkey anti-goat antibody (Bio-connect, 705-065-003, 1:500, RRID: AB_2340396) and biotin-SP-donkey anti-mouse (Bio-connect, 715-065-150, 1:300, RRID: AB_2307438), Alexa Fluor 488 goat anti-mouse IgG3 (Bio-connect, 115-547-189, 1:300, RRID: AB_2632538), Alexa Fluor 647 goat anti-mouse IgG2B (Jackson ImmunoResearch, 115-607-187, 1:300, RRID: AB_2632546).

For flow cytometric analysis and FACS. Antibodies used for murine samples are the following: eFluor506 or eFluor450 Fixable viability dye (eBioscience, 65-0866-14 or 65-0863-18, 1:500), APC-Cy7 or FITC anti-CD45 (BioLegend, 103116 or 103108 , clone 30-FB1, 1:200, RRID: AB_312981 or AB_312973), PerCP-Cy5.5, eFluor450 or PE anti-CD11b (BioLegend, clone M1/70, 101228; eBioscience, clone M1/70, 48-0112-82; BD Biosciences, clone M1/70, 557397; 1:300, RRID: AB_893232, AB_1582236 or AB_396680), BV421 or eFluor450 anti-TCR-β chain (BD Biosciences, 562839 or eBioscience, 48-5961-82, clone H57-597, 1:400, RRID: AB_2737830 or AB_11039532), PE anti-CD4 (BioLegend, 100512, clone RM4-5, 1:500, RRID:AB_312715), PE-Cy7 or APC-Cy7 anti-CD8 (eBioscience, 25-0081-82 or BioLegend; 100714, clone 53-6.7, 1:400, RRID: AB_469584 or AB_312753), APC anti- CD69 (eBioscience, 17-0691-82, clone H1.2F3, 1:200, RRID: AB_1210795), Alexa Fluor 488, PerCP-Cy5.5, BV421 or APC anti-F4/80 (eBioscience, 53-4801-82; BioLegend, 123128 or 123132, Invitrogen, 17-4801-82, clone BM8, 1:200, 1:150 or 1:200, RRID: AB_469915, AB_893484, AB_11203717, AB_2784648), PE-Cy7 anti-IFN-γ (eBioscience, 25-7311-41, clone XMG1.2, 1:100, RRID: AB_1257211), Alexa Fluor 647 anti-GZMB (BioLegend, 515406, clone GB11, 1:100, RRID: AB_2566333), APC or PerCP-eFluor 710 anti-MHC-II (I-A/I-E) (eBioscience, 17-5321-81 or 46-5321-82, clone M5/114.15.2, 1:400, RRID: AB_469454, AB_1834439), PE-Cy7 anti-CD11c (eBioscience, 25-0114-82, clone N418, 1:400, RRID: AB_469590), FITC anti-CD206 (Bio-Rad, MCA2235A647T, clone MR5D3, 1:100, RRID: AB_324622), PE or APC-Cy7 anti-Ly-6G (BDPharmingen, 551461 or BioLegend, 127624, clone 1A8, 1:400, RRID: AB_394208, AB_10640819), APC anti-CD335 (NKp46) (BioLegend, 137608, clone 29A1.4, 1:100, RRID: AB_10612758), APC anti-Foxp3 (eBioscience, 17-5773-82, clone FJK-16s, 1:100, RRID: AB_469457), PE-Cy7 anti-CD25 (eBioscience, 25-0251-82, clone PC61.5, 1:200, RRID: AB_469608), FITC anti-P2RY6 (Alomone Labs, APR-106-F, polyclonal, 1:100), PerCP-Cy5.5 anti-CD274 (BioLegend, 124334, clone 10F.9G2, 1:100, RRID: AB_2629832), Alexa Fluor 488 or PE anti-CD90.1 (BioLegend, 202506 or 202523, clone OX-7, 1:200, RRID: AB_492882, AB_1595635), PE anti-CD90.2 (BD Pharmingen, 553005, clone 53-2.1, 1:100, RRID: AB_394545), APC anti-CD31 (BD Pharmingen, 551262, clone MEC 13.3, 1:100, RRID: AB_398497), BV 421 anti-Ki-67 (BioLegend, 151208, clone 11F6, 1:100, RRID: AB_2629748).
Antibodies used for human samples are the following: BV421 anti-CD14 (Sony Biotech, 2109150, clone M5E2, 1:200), FITC anti-P2RY6 (Alomone Labs, APR-106, polyclonal, 1:100), PE anti-CD80 (BD Pharmingen, 557227, clone L307.4, 1:200, RRID: AB_396606), PE-Cy7 anti-CD115 (Sony Biotech, 2336540, clone 9-4D2-1E4, 1:100), APC anti-HLA-DR (Invitrogen, 17-9956-41, clone LN3, 1:100, RRID: AB_10671395), BV605 anti-CD163 (Sony Biotech, 2268080, clone GHI/61, 1:200), BUV 395 anti-CD206 (BD Pharmingen, 740309, clone 19-2, 1:100, RRID: AB_2740047), PE anti-CD204 (Sony Biotech, 2459520, clone 7C9C20, 1:100), PerCP-Cy5.5 anti-CD11b (Sony Biotech, 2106640, clone ICRF44, 1:300), BV510 anti-CD3 (BD Biosciences, 564713, clone HIT3alpha, 1:200, RRID: AB_2738909), BV 711 anti-CD45 (Sony Biotech, 2120250, clone HI30, 1:200), BUV496 anti-CD15 (BD Biosciences, 741187, clone W6D3, 1:200, RRID: AB_2870752), BV421 anti-CD31 (BioLegend, 303124, clone WM59, 1:100, RRID: AB_2563810), PE and anti-

	CD326 (BD Biosciences, 347198, clone EBA-1, 1:100, RRID: AB_400262), PE-Cy7 anti-MHC Class I (H-2Kb) (Invitrogen, 25-5958-82, clone AF6-88.5.5.3, 1:200, RRID: AB_2573505), PE anti-H-2Kb bound to SIINFEKL (BioLegend, 141603, clone 25-D1.16, 1:100, RRID: AB_10897938).<br><br>For in vivo experiments. Rat serum IgG (Sigma-Aldrich, 14131, 10mg/kg); Ultra-LEAF Purified anti-mouse PD-1 (CD279) (BioLegend, 96167, clone RMP1-14, 10mg/kg); InVivoMAb anti-mouse CD8α (BioCell, BE0004-1, clone 53-6.7, 5 mg/kg).
Validation	The antibodies used are established in the field and have been used by a number of groups. RRID was provided in antibody information listed above.<br>Some antibodies have been additionally validated for use in their respective application.<br>For Western Blot (anti-mouse CDA). KO cells for CDA were used as negative controls. Overexpressing cells for CDA were used as positive controls.<br>For histology and immunostainings. As negative control, one section per slide was stained following the same protocol and omitting the primary antibody.<br>For flow cytometric analysis and FACS. FMO (fluorescence minus one) and isotype controls were evaluated for each antibody to assess specificity in all the stainings.

Eukaryotic cell lines

Policy information about cell lines and Sex and Gender in Research

Cell line source(s)	The murine pancreatic ductal adenocarcinoma Panc02 cell line was kindly provided by Prof. B. Wiedenmann (Charité, Berlin). The murine pancreatic KPC FC1245 and KPC FC1199 cell lines, were generated from the KPC murine model (KrasLSL.G12D/+; p53R172H/+; Pdx1-Cretg/+), and kindly provided by Prof. D. Tuveson (New York, USA).<br>The murine colon carcinoma MC38 cell line was obtained from Kerafast. CT26 murine colon carcinoma cells were purchased from the American Type Culture Collection (ATCC). The melanoma YUMM 1.7 cell line was a kind gift from Prof. R. Marais (Manchester, UK), and was originally obtained from Sigma-Aldrich.
Authentication	An internal golden stock of all cell lines was generated and maintained by the lab manager. One thawed, cells were cultured for no more than ten passages in a humidified incubator in 5% CO2 and 95% air at 37 C.<br>All cell lines were authenticated based on morphological criteria only.
Mycoplasma contamination	Cells lines were tested negative for mycoplasma by PlasmoTest'-Mycoplasm Detection Kit (InvivoGen) within two weeks after thawing.
Commonly misidentified lines<br>(See ICLAC register)	No commonly misidentified cell lines were used.

Animals and other research organisms

Policy information about studies involving animals; ARRIVE guidelines recommended for reporting animal research, and Sex and Gender in Research

Laboratory animals	All mice used were females between 8 and 10 weeks old. Mice were maintained under pathogen-free, temperature- and humidity-controlled conditions under a 12/12-h light/dark cycle and received normal chow (ssniff® R/M-H).<br>C57BL/6 and NMRI-Foxn1nu, mice were purchased from Envigo. OT-I mice were purchased from Taconic. P2Y6 (P2ry6tm1Jabo, MGI:5304911) floxed mouse line in C57BL/6 background was kindly provided by Prof. Joshua A. Boyce (Boston, USA). P2ry6lox/lox;LysM-Cre transgenic mice were generated by intercrossing P2ry6-floxed mice with myeloid specific LysM-Cre deleter mouse line (B6.129P2-Lyz2tm1(cre)Ifo/J, purchased from Jackson Laboratory).
Wild animals	No wild animals were used for this study.
Reporting on sex	All mice used were females between 8 and 10 weeks old.
Field-collected samples	No field-collected samples were used for this study.
Ethics oversight	Housing and all experimental animal procedures were approved by the Institutional Animal Care and Research Advisory Committee of the KU Leuven (ECD P226/2017 and P060/2021). Animals were removed from the experiments and killed if any signs of pain and distress were detected or if the tumor volume went beyond 2000mm3. The maximal tumor size was not exceeded in any reported studies.

Note that full information on the approval of the study protocol must also be provided in the manuscript.

Clinical data

Policy information about clinical studies

All manuscripts should comply with the ICMJE guidelines for publication of clinical research and a completed CONSORT checklist must be included with all submissions.

Clinical trial registration	We run retrospective analyses on patients already enrolled in registered clinical trial (NCT03104439)
Study protocol	All the information related to their study protocol are available at the following link:

Study protocol

https://www.clinicaltrials.gov/study/NCT03104439?id=NCT03104439&rank=1

Data collection

All the information related to the data collection are publicly available in https://www.ClinicalTrials.gov/ (NCT03104439). NCT03104439. Patients enrolled between 07/2017 to 12/2018 in Massachusetts General Hospital (MGH) Cancer Center in Boston, MA as stated in https://www.clinicaltrials.gov/study/NCT03104439?id=NCT03104439&rank=1 and published in PMID: 35122060.

Outcomes

Ethical approval number: NCT03104439. All primary and secondary outcomes are publicly available in https://www.clinicaltrials.gov/study/NCT03104439?id=NCT03104439&rank=1 and published in PMID: 35122060.
DESEq2 pre-normalized data by the original authors was downloaded from GSE179351. Expression of PDCD1 and CDA in PDAC patients prior to treatment with ICB plus radiotherapy in responders and non-responders was analysed and represented as dotplots. Dot sizes represent the proportion of patients with non-zero expression. The color scale represents standard-scaled mean expression per genetic marker. 'NoResponse' includes patients achieving either stable or progressive disease (SD/PD); 'Response' includes patients achieving either partial or complete response (PR/CR) as defined in Supplementary Table 8 of reference23 (NCT03104439).

Flow Cytometry

Plots

Confirm that:

☒ The axis labels state the marker and fluorochrome used (e.g. CD4-FITC).

☒ The axis scales are clearly visible. Include numbers along axes only for bottom left plot of group (a 'group' is an analysis of identical markers).

☒ All plots are contour plots with outliers or pseudocolor plots.

☒ A numerical value for number of cells or percentage (with statistics) is provided.

Methodology

Sample preparation

Tumor-bearing mice were sacrificed by cervical dislocation. Tumors were harvested and minced in alpha-MEM medium (Lonza) supplemented with 5% FBS, 1% Pen/Strep, 50 µM β-mercaptoethanol (Gibco), 5 U/ml DNase I (Qiagen), 0.85 mg/ml Collagenase V (Collagenase from Clostridium histolyticum, Sigma-Aldrich), 1.25 mg/ml Collagenase D (Collagenase from Clostridium histolyticum, Roche) and 1 mg/ml Dispase II (Gibco) and incubated for 30 minutes at 37°C. The digested tissue was filtered using a 70 µm pore sized mesh strainer and cells were centrifuged 5 minutes at 300 x g. The samples were resuspended in Red Blood Cell Lysing Buffer Hybri-MaxTM (Sigma-Aldrich) for 30 seconds, inactivated with FACS buffer (PBS containing 2% FBS and 2 mM EDTA) and centrifuged. The cell pellets were resuspended in FACS buffer and filtered with a 40 µm pore sized mesh strainer. Single cell suspension were centrifuged 5 minutes at 300 x g and cell pellet were resuspended in FACS buffer for antibody staining.

Instrument

FACS LRSFortessa X-20 (BD Bioscience, model number 658226R1); FACSAria Fusion Cell Sorter (BD Bioscience).

Software

Flow cytometry data collection was done with BD FACs DIVA software (v9.0)
Flow cytometry analysis was done with the FlowJo software (v10.8.1).

Cell population abundance

We performed post-sort flow cytometric analyses of each sorted cell population demostrating that the purity exceeded 94% and most importantly reached around 99% in most of them (Supplementary Figure 1-4).

Gating strategy

Cell substes were gated as follow: within the CD45 positive gate, we defined TAMs as CD45 positive, CD11b positive and F4/80 positive. M1-like TAMs were gated as CD11c positive or MHC-II high, and M2-like TAMs as CD206 positive, out of F4/80 positive. T cells were gated for FSC/SSC, CD45 positive, CD11b negative and TCRβ positive. T helper (CD4 positive) and cytotoxic (CD8 positive) T cells were gated out of TCRβ positive. Out of CD4 positive, Tregs were gated for Foxp3 positive, CD25 positive. Out of CD8 positive, activated CD8+ T cells were gated for CD69 positive, IFN-γ positive or GZMB positive, proliferating CD8+ T cells were gated for Ki67 positive. Neutrophils were gated for FSC/SSC, CD45 positive, CD11b positive and Ly6G positive, or alternatively, F4/80 negative. NK cells were gated for FSC/SSC, CD45 positive and NKp46 positive. DCs were gated for FSC/SSC, CD45 positive, CD11b positive, F4/80 negative, CD11c positive. B cells were gated for FSC/SSC, CD45 positive, CD11b negative, CD45R positive, or alternatively, TCRβ negative. Fibroblasts were gated for FSC/SSC, CD45 negative, CD90.1 (congenic marker of cancer cells) negative and CD90.2 positive. Endothelial cells were gated for FSC/SSC, CD45 negative and CD31 positive. Cancer cells were gated for FSC/SSC, CD45 negative and positive for the congenic marker CD90.1 and CD90.2 negative. All the gatings were performed on the viable cell fraction, excluding all the cells positive for the viability dye (eBioscience™ Fixable Viability Dye eFluor™ 506 or eBioscience™ Fixable Viability Dye eFluor™ 450, depending on the panel composition). See Supplementary Figure 5 and 6.

☒ Tick this box to confirm that a figure exemplifying the gating strategy is provided in the Supplementary Information.
