# Supplementary material for: Nucleotide metabolism in cancer cells fuels a UDP-driven macrophage cross-talk, promoting immunosuppression and immunotherapy resistance
Source: Nat Cancer. 2024 Jun 6;5(8):1206–26. doi: 10.1038/s43018-024-00771-8 (PMC11358017; doi:10.1038/s43018-024-00771-8)

Generation of CDA-depleted Panc02, KPC FC1245 and YUMM 1.7 cell lines

Extended Data Figure 2b, 2g and Extended Data Figure 3e

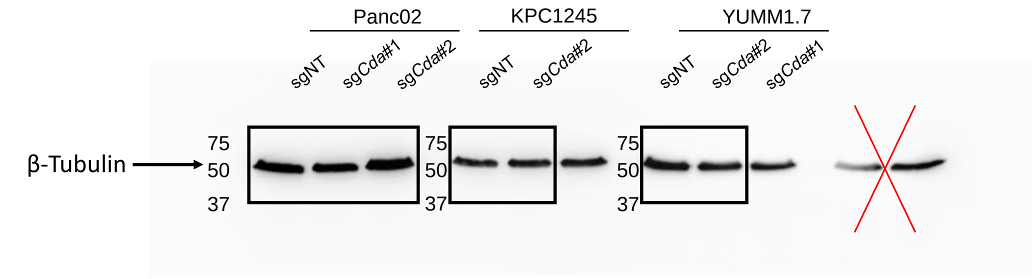

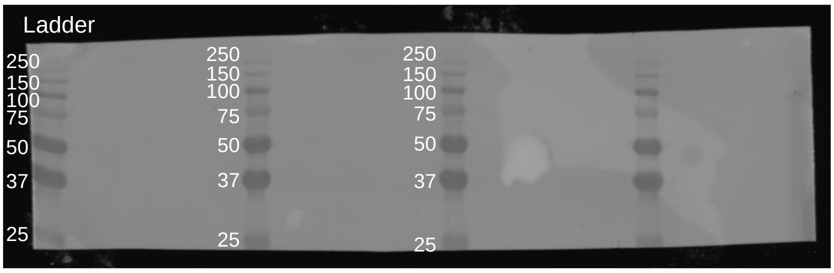

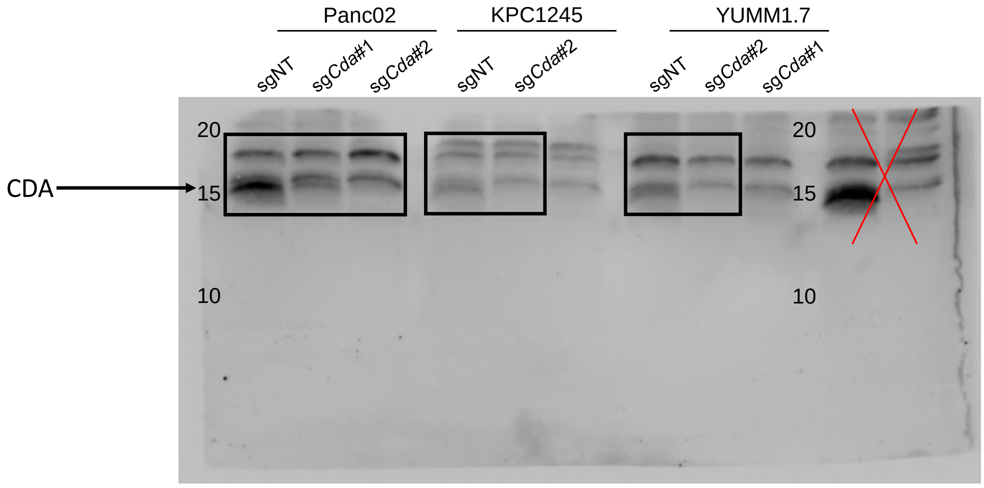

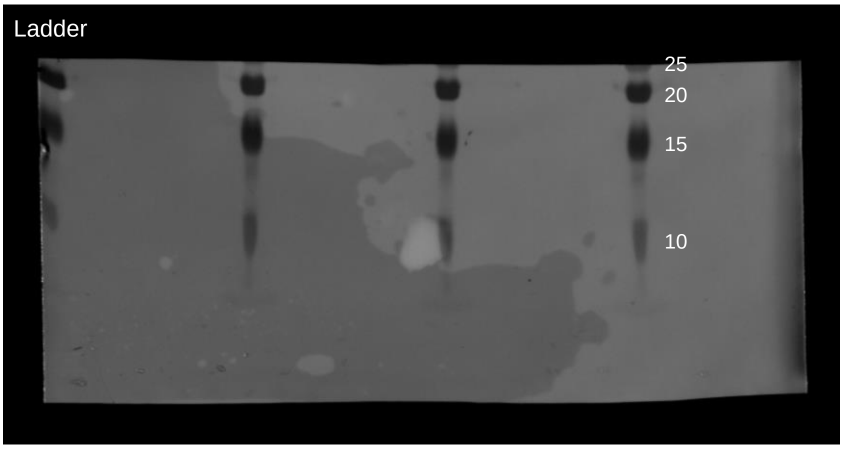

Generation of CDA-depleted KPC FC1199 cell line

Extended Data Figure 2j

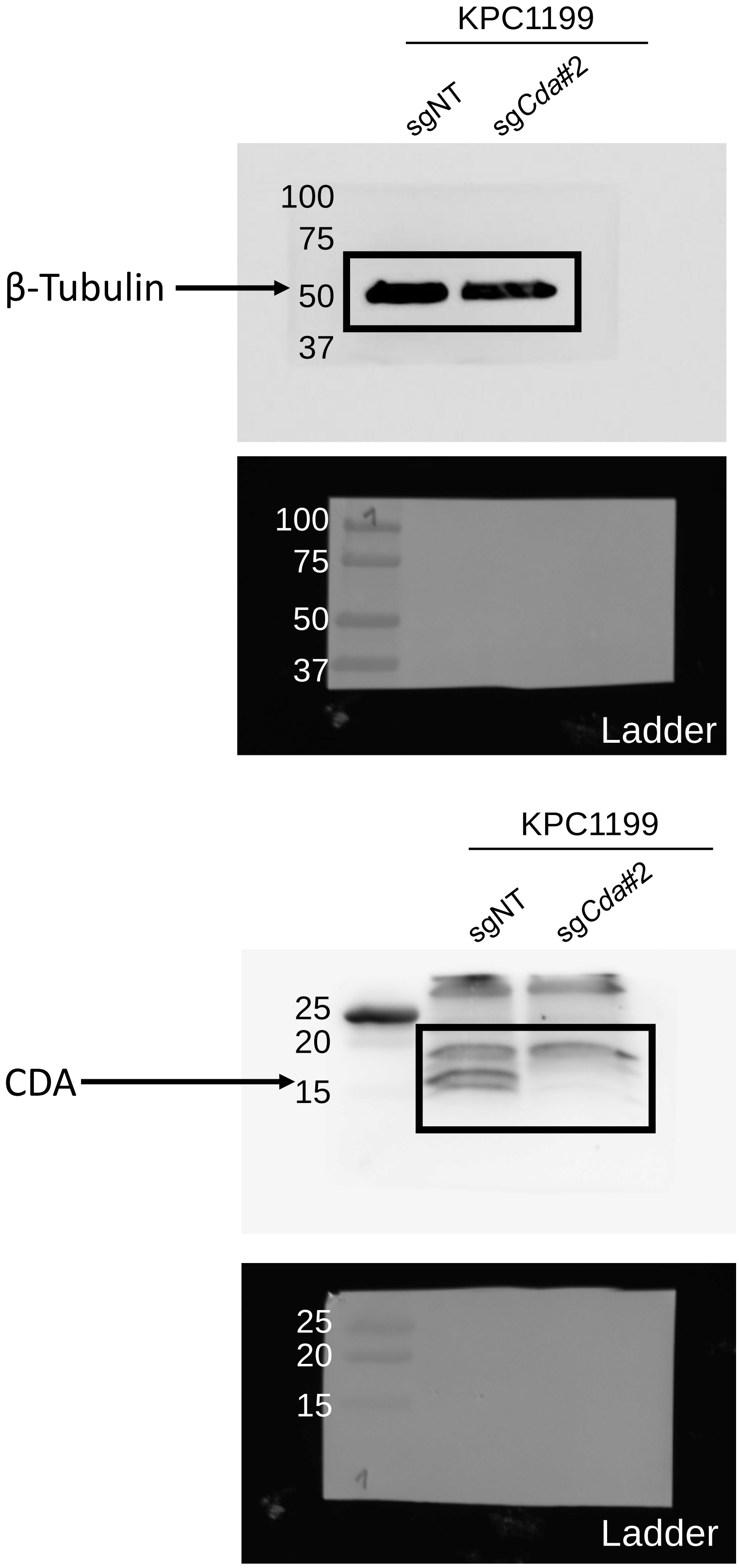

Generation of CDA overexpressing KPC FC1245 cancer cells

Extended Data Figure 2p

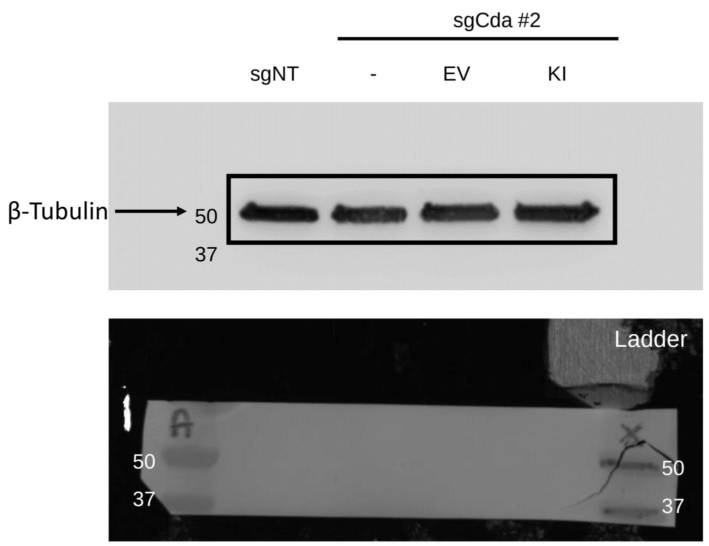

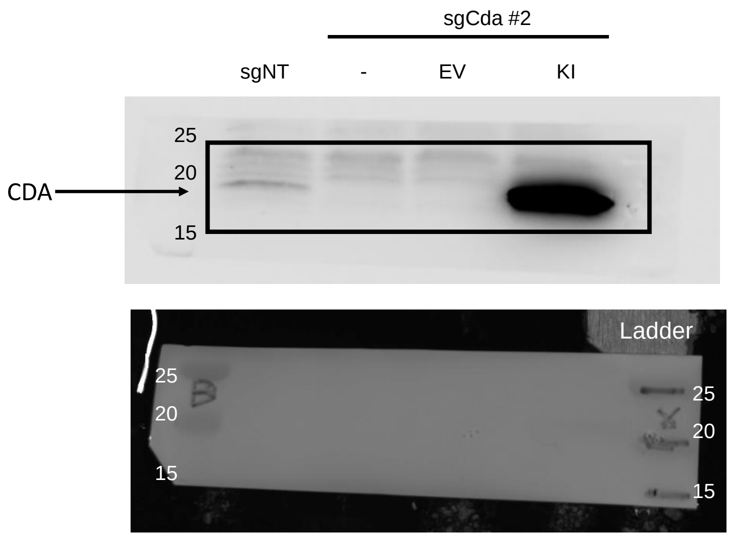

Generation of CDA overexpressing MC38 cancer cells

Extended Data Figure 3i

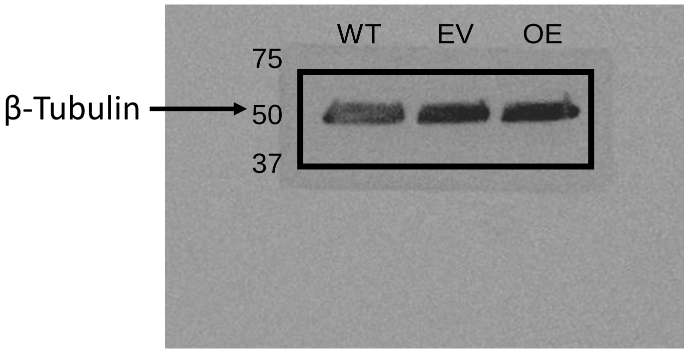

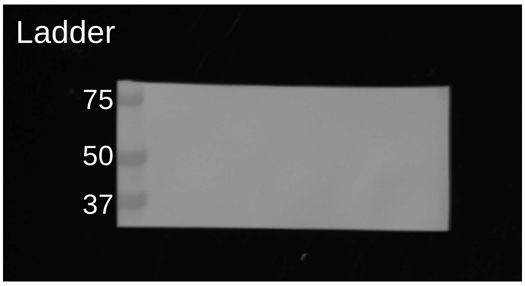

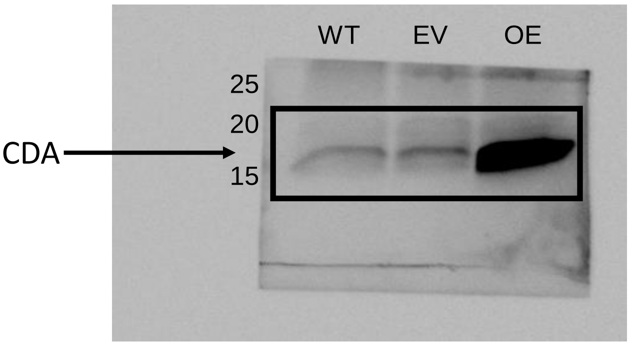

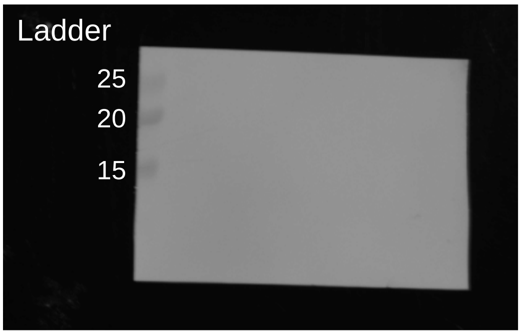

Supplement: Supplementary file 19 — Unprocessed western blots. [file 43018_2024_771_MOESM19_ESM.pdf]
